# Pesticide risk to managed bees during blueberry pollination is primarily driven by off-farm exposures

**DOI:** 10.1038/s41598-022-11156-1

**Published:** 2022-05-03

**Authors:** Kelsey K. Graham, Meghan O. Milbrath, Yajun Zhang, Nicolas Baert, Scott McArt, Rufus Isaacs

**Affiliations:** 1 Department of Entomology, Michigan State University, 202 CIPS, 578 Wilson Road, East Lansing, MI 48824 USA; 2 Department of Entomology, Cornell University, 4129 Comstock Hall, Ithaca, NY 14853 USA; 3 Present Address: Pollinating Insect – Biology, Management, Systematics Research Unit, U.S. Department of Agriculture – Agricultural Research Service, 1410 N 800 E, Logan, UT 84341 USA

**Keywords:** Agroecology, Ecosystem services

## Abstract

When managed bee colonies are brought to farms for crop pollination, they can be exposed to pesticide residues. Quantifying the risk posed by these exposures can indicate which pesticides are of the greatest concern and helps focus efforts to reduce the most harmful exposures. To estimate the risk from pesticides to bees while they are pollinating blueberry fields, we sampled blueberry flowers, foraging bees, pollen collected by returning honey bee and bumble bee foragers at colonies, and wax from honey bee hives in blooming blueberry farms in southwest Michigan. We screened the samples for 261 active ingredients using a modified QuEChERS method. The most abundant pesticides were those applied by blueberry growers during blueberry bloom (e.g., fenbuconazole and methoxyfenozide). However, we also detected highly toxic pesticides not used in this crop during bloom (or other times of the season) including the insecticides chlorpyrifos, clothianidin, avermectin, thiamethoxam, and imidacloprid. Using LD_50_ values for contact and oral exposure to honey bees and bumble bees, we calculated the Risk Quotient (RQ) for each individual pesticide and the average sample RQ for each farm. RQ values were considered in relation to the U.S. Environmental Protection Agency acute contact level of concern (LOC, 0.4), the European Food Safety Authority (EFSA) acute contact LOC (0.2) and the EFSA chronic oral LOC (0.03). Pollen samples were most likely to exceed LOC values, with the percent of samples above EFSA’s chronic oral LOC being 0% for flowers, 3.4% for whole honey bees, 0% for whole bumble bees, 72.4% for honey bee pollen in 2018, 45.4% of honey bee pollen in 2019, 46.7% of bumble bee pollen in 2019, and 3.5% of honey bee wax samples. Average pollen sample RQ values were above the EFSA chronic LOC in 92.9% of farms in 2018 and 42.9% of farms in 2019 for honey bee collected pollen, and 46.7% of farms for bumble bee collected pollen in 2019. Landscape analyses indicated that sample RQ was positively correlated with the abundance of apple and cherry orchards located within the flight range of the bees, though this varied between bee species and landscape scale. There was no correlation with abundance of blueberry production. Our results highlight the need to mitigate pesticide risk to bees across agricultural landscapes, in addition to focusing on the impact of applications on the farms where they are applied.

## Introduction

Managed pollinators are an increasingly important component of profitable commercial agriculture, providing the movement of pollen between anthers and stigmas that allows for full expression of potential crop yields^1,2^. As agriculture has progressively intensified, the suitability of land for wild pollinators has declined, thereby increasing dependence on managed pollinators such as honey bees and bumble bees^3–5^. Growers of pollinator-dependent crops may use synthetic chemical inputs to reduce crop losses from pests, but these chemicals can be hazardous to the pollinators required for crop yields. Balancing these competing needs has been termed Integrated Pest and Pollinator Management (IPPM)^6^, a system that requires information on the costs and benefits of different approaches to pest management and the implications for pollinators and for crop yield^7,8^.

Managed bees are exposed to pesticides through multiple routes. Bees foraging on crop flowers can be exposed by direct sprays, through contact with flowers, and orally through ingested pollen and/or nectar^9^. Bees in the colony can be exposed orally and through contact during the consumption and processing of contaminated water, nectar, and pollen^10^. Finally, bees can be exposed orally to chemicals in wax (when chewing wax, or when chemicals in wax partition into food) as well as through contact with pesticides in the wax matrix of the hive^11,12^. Estimates of pesticide exposure therefore require quantitative measurements from a variety of matrices relevant to bees. Analytical methods for quantifying pesticide residues in biological matrices have become increasingly sensitive, allowing for parts per billion detection^13^. Through widespread adoption of the QuEChERS method^14^, the cost of sample processing has declined and these methods have been optimized for extraction of residues from pollen, flowers, bees, and wax^15,16^. While the majority of pesticide exposure studies in bees have thus far focused on bee collected pollen^17–25^, some have also included other bee relevant matrices, primarily honey bee wax and whole bees^9,26–30^.

To develop integrated pest and pollinator management plans^6^, growers need timely information regarding potential hazards to pollinators from crop-applied pesticides. However, assessing risks to managed bees in commercial agriculture settings is difficult. Colonies of honey bees and bumble bees are dynamic complex systems whose overall health is an emergent property of the balance of the workers, larvae, and pupae that comprise a superorganism^31^. Pesticides can have sub-lethal effects that disrupt this balance^32^ but these are challenging to measure directly. Furthermore, dose estimation for even a single pesticide is complicated by consumption rates and exposure pathways varying for different bee species and ages^33,34^. Additionally, multiple active ingredients in combination may have synergistic effects, further complicating estimates of risk^35–37^. We previously estimated the concentrations of active ingredients in pollen collected by honey bees and bumble bees returning to colonies in blueberry fields during bloom^38^. Eighty active ingredients were found in pollen samples, in different combinations that varied over time (the same farm in 2 years) and space (multiple farms in the same year), highlighting the wide range of pesticides that bees can be exposed to. However, these pesticides vary widely in their toxicity to bees, so it is important to understand which pose the greatest risk.

One method to quantify risk is the hazard quotient (HQ)^24,39^ or risk quotient (RQ)^40^, these equivalent values combine the concentration of an active ingredient and its toxicity (LD_50_) (we will hereafter use RQ). While originally developed for comparing risk of sprays, this has recently been used for in hive matrices, because it accounts for highly toxic ingredients present in low concentrations. Samples with multiple residues can have the RQ values summed to compare the relative level of pesticide risk between sites or over time. RQ values can then be considered in the context of the Environmental Protection Agency (EPA)^40^ and European Food Safety Authority (EFSA)^39^ levels of concern (LOCs) for pesticide risk. Use of a risk quotient is relatively common^18,19,22–26,29^, though previous studies have not always related the calculated RQ to a regulatory agency’s levels of concern. This approach has been used for pesticide risks to honey bees in New York apple orchards^25^, revealing significant levels of risk to some colonies. The study linked pesticide risks to sources outside apple orchards where the bee colonies were located, showing that combining exposure data, toxicity information, and landscape analysis can provide insights into sources of pesticide risk. Our recent analysis of pesticide residues in pollen trapped at the entrances to colonies of honey bees and bumble bees in blueberry fields for pollination^38^ revealed that pesticides applied for disease and insect control were at the highest concentrations, but many of the other pesticides detected were not used in this crop during bloom or at other times of the year. This indicated that bees were collecting some of the contaminated pollen from crops across the landscape, supported by correlations to landscape composition.

In this study, we expanded on the results presented in Graham et al.^38^ by sampling a greater number of relevant bee and plant matrices to develop a more complete view of pesticide risk to managed honey bees and bumble bees during blueberry pollination. This included the collection of whole blueberry flowers, which would include exposure from nectar, pollen, or contact with flower parts such as the corolla. All of these potential routes of exposure are relevant for bees visiting for collection of pollen and/or nectar. We also collected samples of foraging honey bees and bumble bees, bee-collected pollen, and honey bee hive wax in blueberry fields during bloom. Collecting these diverse bee-relevant matrices allowed us to compare these different routes of exposure in relation to pesticide risk (RQ). In this study we analyzed pesticide residues to determine: (1) the residue levels in these different types of bee related matrices; (2) the risk quotients associated with individual detections and whole samples, (3) the degree of consistency in pesticide risk among sites and between years; and (4) the role of farm management and landscape composition on pesticide risks to managed bees.

## Methods

### Sampling in Year 1 (2018): Honey bee pollen collections

In 2018, we sampled pollen from commercial honey bee colonies at 14 highbush blueberry farms in southwest Michigan (Berrien, Van Buren, and Allegan counties) (Fig. S1). Blueberry fields were managed by eight growers, where seven of the fields were managed using conventional pest management (referred to as conventional), three using organic pest management (organic), and four had no chemical pest management at any time of the year (unsprayed) (Table S1). The average distance between sampled fields was 21.6 km ± 11.8 S.E. (min = 1.8 km). Pollen collection is described in detail in Graham et al.^38^ and summarized briefly here. We sampled pollen from colonies delivered by commercial beekeepers in the field margins at each farm. Each farm had different stocking rates of honey bees, with an average of 82 colonies per site (range: 28–260). Three average-sized (mean = 11.72 frames of bees), queen-right colonies were haphazardly selected from those at each site (N = 42 colonies across the 14 sites) before the start of blueberry bloom (week of May 14th, 2018). Each selected colony had a 10-frame superior pollen trap (Mann Lake, Hackensack, MN) installed that was continuously engaged throughout bloom. We collected pollen from each hive twice (dates of sampling are provided in Table S1), though because of some trap failures (e.g., bees finding ways into the hive that avoided the pollen trap), we collected an average of 1.81 ± 0.09 S.E. samples per hive. Samples were brought to the laboratory and stored at − 20 °C until processing. For pesticide residue analysis, we subsampled 5 g from each sample (76 pollen samples collected over the season) with an average weight of 5.02 g ± 0.00 S.E.

Spray records were collected from all fields to understand pesticide applications on the focal farm during bloom at the locations where managed bee colonies were placed (Table S1).

### Sampling in Year 2 (2019): Honey bee pollen collections

In 2019, we again sampled honey bee pollen from 14 sites (Fig. S1, Table S1). Seven of the sites were the same as in 2018 (four conventional fields and three unsprayed fields). In addition to these seven, we sampled at eight fields in the same region for a total of 14 blueberry fields managed by six growers (nine conventional, five unsprayed, and no organic) (Fig. S1, Table S1). The average distance between sites was 16.7 km ± 9.0 (min = 2.0 km). The same sampling methods were used as in 2018, with sampling starting the week of May 6, 2019. We sampled each hive three times in 2019 for an average of 2.31 ± 0.17 samples per hive (reductions again due to trap failures) (sampling dates are provided in Table S1). Again, 5 g of pollen were subsampled from each pollen sample (97 samples in 2019), except for three samples which had less than 5 g of pollen available, in which case smaller amounts were used (small sample weight range = 1.46–3.52 g). The average sample weight was 4.92 g ± 0.06.

Again, spray records were collected from all growers to understand pesticide application during bloom on the focal farms where bees were placed (Table S1).

### Sampling in Year 2 (2019): Bumble bee pollen collections

We placed *Bombus impatiens* QUAD colonies (four colonies per QUAD, Koppert Biological Systems, Howell, MI) in the field margins of each field where honey bee pollen was collected in 2019. We also placed a QUAD at an additional site (conventionally managed) where honey bees were not sampled for a total of 15 sites with bumble bee colonies (total of 60 colonies across 15 sites). Bumble bee colonies were placed in the field margin the same week as honey bee colonies (week of May 6, 2019). We hand-collected pollen from returning bumble bee foragers four times at each colony during blueberry bloom (May 14–June 6, 2019), for a total of 240 samples (sampling dates are provided in Table S1). This involved trapping returning foragers at the colony entrance and removing pollen from their corbiculae (Fig. S2). Each sampling visit lasted 1.5 h with two people monitoring the entrances to the four colonies. Collected pollen was then stored at -20 °C until processing. Due to relatively low bumble bee pollen volumes, we combined all pollen from a site into a single sample for residue analysis (range = 2.02–4.95 g, average 3.29 g ± 0.21).

### Sampling in Year 2 (2019): Flower collections

We collected 10 blueberry flower clusters (approximately 4–10 individual flowers per cluster, Fig. S2) at each site from the field adjacent to the bumble bee colonies at various times during bloom. Samples were collected when restricted entry intervals had expired (as listed on the pesticide product label) and when the weather was suitable for bee foraging (sampling dates listed in Table S1). Clusters were chosen haphazardly when walking up and down the blueberry rows. Using metal scissors, we clipped flower clusters into a labeled Ziploc gallon bag. Scissors were thoroughly cleaned with 70% ethanol between each site. Forty flower samples were collected across 15 farms (average number of samples per farm = 2.7 ± 0.2 S.E.). Prior to residue analysis, the calyx and stems pieces were cut away from the corolla, and only the corolla and internal morphology (stamens and pistil) were included for residue analysis. The average weight of flower samples was 4.8 g ± 0.1.

### Sampling in Year 2 (2019): Bee collections

On the same days as when we collected bumble bee pollen (four times during bloom) we collected honey bees nectaring on blueberry flowers for 30 min or up to 10 bees (there were no instances of sampled bees collecting pollen) (sampling dates provided in Table S1). Collections were done by putting a cyanide kill jar under/around the bee while it was nectaring so that it dropped into the jar (Fig. S2). After the 30 min/10 bee collection period, dead bees were removed from the container and transferred to a labeled collection bag. The cyanide kill jar was then wiped down with 70% ethanol. During the same visits, we also collected approximately 10 foraging bumble bees for 30 min using an aerial net. However, this almost always resulted in fewer than 10 bumble bees, as they were much less common than honey bees who are stocked at high rates for blueberry pollination. Bees were captured when foraging for nectar and pollen and when flying between flowers. The species collected were *Bombus impatiens*, *B. griseocollis*, and *B. bimaculatus*, many of which were queens (9 out of 47), as natural populations of bumble bees in the area would still be in the very early colony founding phase with queens doing the bulk of foraging.

A total of 338 honey bees and 47 bumble bees were collected across all sites. For sampling events with fewer than 10 bees, we created a composite sample of bees (keeping *Apis* and *Bombus* separate) from the same site collected over several days (*Apis*—mean number of samples per site: 1.9 ± 0.2 S.E.; sample weight range: 0.6–1.9 g; number of bees per sample: 5–19. *Bombus*—mean number of samples per site: 0.8 ± 0.0; sample weight range: 0.2–4.4 g; number of bees per sample: 1–9). At three sites, no bumble bees were collected even with several attempts over multiple days. Pollen was removed from the legs and discarded before pesticide residue analysis.

### Sampling in Year 2 (2019): Wax collections

Wax was sampled from honey bee colonies 2 weeks after they were moved by the beekeeper to a honey yard following blueberry pollination (samples taken June 21–29, Table S1). We collected 113 wax samples from hives that were previously at 13 of the blueberry fields included in this project, eight conventional farms and five unsprayed farms. This included samples from 8 to 9 hives per field, and each hive was only sampled once. Following methods from the APHIS National Honey Bee Disease Survey Protocol^41^, we selected a frame from the brood nest with an area of empty drawn comb. We placed the flat end of a clean hive tool directly into the wax, pivoting the tool 90° to cut out a quarter of a circle (with a radius the width of the hive tool). The scraped wax was placed into a labeled 50-mL Falcon tube, and tubes were placed on dry ice for transport and stored at − 20 °C until processing. The samples weighed on average 1.16 g ± 0.05 S.E.

### Residue analyses

All samples (blueberry flowers, honey bees, bumble bees, bee collected pollen, and wax) were shipped overnight on dry ice to Cornell University (Ithaca, NY). Frozen samples were extracted by a modified version of the EN 15662 QuEChERS procedure^42^ and screened for 261 active ingredients (AIs) (including some metabolites and breakdown products) by liquid chromatography mass spectrometry (LC–MS/MS). An internal standard solution (d_4_-imidacloprid 0.07 ng/µL; d_10_-chlorpyrifos 0.2 ng/µL: d_7_-bentazon 0.1 ng/µL; d_5_-atrazine 0.02 ng/µL; d_7_-propamocarb 0.1 ng/µL) was used. Detailed methods can be found in Graham et al.^38^. Retention times and optimized SRM acquisition parameters are provided in Table S1. Limits of detection, quantification, and upper limits of linearity are provided in Table S2.

### Landscape classification

We used ArcGIS v10.2.2 (Environmental Systems Research Institute, Redlands, CA, USA) to quantify the proportion of the surrounding landscape in different land uses at spatial scales based on typical bee foraging distances^43–45^ (500, 1000, and 2000 m). Land was categorized based on the Cropland Data Layer^46^ (CDL; 30 m spatial resolution) from USDA NASS, supplemented with a layer of blueberry fields that was hand digitized and ground-truthed based on National Agriculture Imagery Program 1 m resolution aerial images. The detailed land cover categories (n = 52) were then reclassified into categories of blueberry, cherry, apple, other agriculture, and other, and we determined the percent of these categories across the three scales surrounding each focal field.

### Data analyses

Analyses and data visualization were performed using R version 4.1.1^47^ and GraphPad Prism 7^48^. All models were checked for overdispersion and zero inflation prior to model selection (function: simulateResiduals, package: DHARMa^49^; simulations = 1000), and model selection was performed by comparison of AICc (function: dredge, package: MuMIn^50^). P values were obtained using the Anova function (package: car^51^) and pseudo-R^2^ values were obtained using the rsquared function (package: piecewiseSEM^52^).

#### Residues on blueberry flowers, whole bees, bee collected pollen, and honey bee wax

Pesticide residue amounts were quantified in parts per billion. For detections that were below the limit of quantification but above the limit of detection, the limit of detection was used in data analyses and summaries. We used a one-way ANOVA with Tukey’s post-hoc test to compare the average number of AIs and average sample concentrations in the different sample types (pollen, flowers, whole bees, and wax). To test if there was a significant difference in residue concentrations in samples between conventional and unsprayed farms, we used a linear mixed model with sample concentration (ppb) (sum of all individual AI concentrations in a sample) as the response variable and management type as the factor of interest. Farm was included as a random effect, and concentration data were log transformed to normalize the distribution. To determine if average number of active ingredients were different between farm management types, we used a generalized linear mixed model with number of AIs as the response variable. Again, farm was included as a random effect, and the model used a Poisson distribution with a log link. This model structure was used to compare concentrations and number of active ingredients between flower samples and between honey bee wax samples collected from different farm management types. For pollen and whole bee analyses, we separated the data for each bee species, and used the model structure as above to compare pesticide concentration and number of AIs between farm management types. We then combined the data to compare between species.

#### Calculating risk

We calculated Risk Quotient (RQ)^24^ values to estimate the risk associated with pesticide exposures from each type of sample. This included flowers (using honey bee and bumble bee toxicity data separately), whole honey bees, whole bumble bees, pollen (each bee/year combination), and wax. RQ values were calculated by dividing the concentration of each AI detected in the sample (ppb) by its LD_50_ (µg/bee). This calculation was performed twice for each sample, using oral and contact LD_50_ values. Sample RQs were calculated as the sum of all individual detection RQs in a sample, which assumes that pesticides are additive (though there is evidence for synergism^35–37^).

To relate RQ values to the EPA and EFSA levels of concern for pesticide risk we then related the RQ values to the proportion of LD_50_. This assumes that an average honey bee adult weighs 100 mg that acute contact exposure occurs from contacting a body-weight equivalent of pollen over 2–4 days, and that chronic oral exposure occurs from ingesting 9.4 mg of pollen per day for 10 days, corresponding to an LC_50_ 10 days chronic exposure^39^. These are likely underestimates of exposure for pollen-provisioning nurse bees. Estimates for bumble bees are not possible due to uncertainties around pollen collection and consumption amounts and a wide variation in body size of *B. impatiens* workers. We therefore used honey bee estimates for both honey bee and bumble bee exposure data. The EPA Tier 1 risk quotient is 0.4 for acute contact exposure, and EFSA uses a similar risk quotient of 0.2. EFSA also considers an LC_50_ 10 days chronic oral exposure risk quotient of 0.03. An equivalent LC_50_ 10 days chronic oral exposure risk quotient is not available from the EPA at this time. We present our data in the context of these levels of concern (LOC). While neither EPA or EFSA have listed acute LOCs for oral exposure, we use the contact acute LOCs (RQ > 0.4 and 0.2) with the oral exposure data in addition to the chronic exposure LOC from EFSA (RQ > 0.03) to highlight particularly high risk quotients. However, the relevance of these benchmarks using oral exposures for bee health is not clear.

LD_50_ values for contact and oral toxicity were primarily obtained from the University of Hertfordshire Agricultural Substances Database (PPDB) (http://sitem.herts.ac.uk.proxy1.cl.msu.edu/aeru/ppdb/en/index.htm). If no relevant LD_50_ values were available in the PPDB, then LD_50_ values from published bee toxicity studies were used when available in the literature (Table S3). Honey bee LD_50_ values were used for calculating RQs from honey bee collected pollen, wax, whole honey bees, and flowers (assuming honey bee contact with flowers). *Bombus terrestris* LD_50_ values were used whenever possible for calculating RQs for *B. impatiens* collected pollen, whole bumble bees, and flowers (assuming bumble bee contact with flowers). If no LD_50_ values for *B. terrestris* were available, we used honey bee LD_50_ values. If no LD_50_ values were available for honey bees, the AI was removed from the analysis, resulting in 11 pesticides being excluded from our contact RQ estimates, and 22 excluded in the oral RQ estimates (Table S3).

We compared average sample RQ between sample types (blueberry flowers, whole bees, pollen, and wax) using a linear mixed effects model with sampleID (sample type + bee species + year) as the factor of interest and farm as a random effect. The RQ value from each sample was log transformed to normalize the data distribution prior to analyses. We used a Tukey’s test to compare between sample types (function: glht, package: multcomp^53^).

#### Active ingredient contribution to RQ

To estimate which AIs represented the greatest risks to bees, we determined what percent of the overall RQ each AI represented. The overall contribution of AIs to the RQ considers the RQ value of individual detections and the frequency with which the AI is encountered, with a high contribution percent resulting from an AI with a high RQ (high toxicity and/or high concentration) and a high frequency of detection (Eq. 1).

1 $$\% Contribution \,of\, an \,AI\, to\, overall\, RQ = \frac{{\left[ {sum \,of \,all\, detection \,RQs \,for \,that \,AI} \right]}}{{\left[ {sum\, of\, all\, detection\, RQs} \right]}} \times 100$$

For data visualization, only AIs that contributed at least 5% to the overall RQ were displayed individually. All those that contributed less than 5% were grouped into the “other” category.

We also used pesticide labels to determine the contribution of active ingredients applied to blueberries to the overall RQ values. This included pesticides registered for use on blueberries at any time of the year.

#### Farm management and bee species

To determine if farm management affected sample RQ, we used a linear mixed model (function = lmer) with management type as the factor of interest and site as a random effect for honey bee pollen data, with separate models for 2018 and 2019. The sum of individual detection RQs in each sample was log transformed to normalize the data distribution prior to analyses. The same analyses were done for bumble bee pollen data in 2019 but with a linear model (no random effect) as there was only one value per site. To determine if there was a significant difference in sample RQ between bee species in 2019, we used a linear mixed model as above, but with bee species as the factor of interest and site as the random effect. Again, sample RQ was log transformed. Separate analyses were also done using contact or oral toxicity data. The same model structure was used to test if site management affected sample RQ for wax, whole bees, and flowers.

#### Correlation of RQ among sites

We tested for correlation in average sample RQ (pollen data only) between the same sites in 2018 and 2019 (honey bee pollen data only) and between honey bee pollen and bumble bee pollen at the same sites in 2019. This was done for RQs from contact and oral toxicity. To account for any differences in average RQ between species or years, we rescaled the average sample RQ data within each year/species combination between 0 and 1 to allow for more direct comparisons of the data between sites (Eq. 2).

2 $$x_{rescaled} = \frac{{x - x_{\min } }}{{x_{max} - x_{min} }}$$

#### Landscape analysis

To determine which aspects of landscape composition correlated with average pollen sample RQ, we calculated a correlation matrix (method: Pearson; function: rcorr.adjust, package: Rcmdrmisc^54^) for each bee species and year. *p* values were corrected for multiple inference using Holm’s method. To determine the effects of scale on the relationships, this was done at three landscape radii (500 m, 1000 m, and 2000 m), with each correlation matrix calculated independently.

## Results

### Pesticides in blueberry flowers, whole bees, bee-collected pollen, and honey bee wax

The majority of frequently detected pesticides were those registered for use on blueberries (Table S4). However, in flower samples the herbicide atrazine was found in 93% of samples, despite not being registered for use on blueberry bushes. In whole bees, the most commonly detected pesticides included fenbuconazole and boscalid, both fungicides applied to blueberry fields in bloom. Eight AIs were detected in over 90% of all pollen samples: atrazine, azoxystrobin, boscalid, chlorpyrifos, fluopyram, imidacloprid, metolachlor, and pyraclostrobin. All of these are applied to blueberries during bloom except imidacloprid which is only registered on this crop after bloom, and atrazine and chlorpyrifos which are not registered for use on blueberries. In honey bee wax, three AIs were detected in over 90% of samples: azoxystrobin, methoxyfenozide, and coumaphos. Methoxyfenozide is an insect growth regulator (IGR) applied to blueberry bushes during bloom for fruitworm control. Coumaphos is a miticide applied to colonies by beekeepers to reduce varroa mite infestations.

The different sample types had significantly different average number of AIs per sample (R^2^ = 0.72, F_3377_ = 324.3, *p* < 0.001), with all being significantly different from the other sample types (Tukey’s *p* < 0.05) (Table 1). Pollen samples had the highest average number of AIs (22.0 ± 0.3 S.E.), followed by wax samples (14.7 ± 0.4), flowers (7.8 ± 0.5) and whole bee samples (4.9 ± 0.4) (Table 1). The different sample types also had significantly different average pesticide concentrations (R^2^ = 0.06, F3377 = 7.5, *p* < 0.001). However, in the post-hoc analysis only whole bees had a significantly higher average sample concentration (1672.6 ± 418.4) compared to all the other sample types (Tukey’s *p* < 0.05) (Wax: 598.0 ± 182.3; Pollen: 485.7 ± 57.5; Flowers: 483.3 ± 99.1) (Table 1).

**Table 1:** Summary table of pesticide detections from pollen collected from honey bees or bumble bees, honey bee wax, whole bees collected from blueberry flowers, and blueberry flowers on blueberry farms in southwest Michigan.

Material	Year	Farm management	# of AIs	AIs per sample (mean ±)	Pesticide concentration (mean ppb ±)
Blueberry flowers, n = 40	2019	Unsprayed	19	5.1 ± 0.9^B^	3.3 ± 1.3^b^
		Conventional	21	8.9 ± 0.4^A^	688.9 ± 122.6^a^
Honey bees (whole bees), n = 29	2019	Unsprayed	10	3.0 ± 1.1^B^	219.0 ± 151.2^b^
		Conventional	16	5.6 ± 0.3^A^	2579.6 ± 687.0^a^
Bumble bees (whole bees), n = 13	2019	Unsprayed	9	2.8 ± 0.9^B^	541.2 ± 132.2^a^
		Conventional	15	5.4 ± 1.1^A^	680.8 ± 259.8^a^
Honey bee pollen*, n = 76	2018	Unsprayed	48	21.6 ± 0.5^B^	85.8 ± 11.8^b^
		Organic	46	26.0 ± 0.5^A^	180.8 ± 29.6^ab^
		Conventional	54	21.2 ± 1.1^B^	286.2 ± 34.1^a^
Honey bee pollen*, n = 97	2019	Unsprayed	55	20.5 ± 0.5^A^	287.3 ± 81.7^b^
		Conventional	61	20.6 ± 0.7^A^	770.0 ± 139.3^a^
Bumble bee pollen*, n = 15	2019	Unsprayed	29	17.8 ± 1.5^A^	330.3 ± 116.8^b^
		Conventional	31	19.1 ± 0.5^A^	1708.2 ± 226.6^a^
Honey bee wax, n = 113	2019	Unsprayed	44	14.2 ± 0.8^A^	710.0 ± 412.2^a^
		Conventional	49	15.0 ± 0.5)^A^	525.6 ± 141.3^a^

Number of active ingredients (AIs) detected includes all pesticides detected within a farm type (unsprayed, organic, or conventional) in each year. Superscript letters indicate significant differences within a year/bee combination.

*Pollen data from Graham et al.^38^.

Blueberry flower samples and whole bees from conventional farms had significantly more active ingredients (Flowers: 8.9 ± 0.4 S.E.; Whole honey bees: 5.6 ± 0.3; Whole bumble bees: 5.4 ± 1.1) compared to those collected from unsprayed farms (Flowers: 5.1 ± 0.9; Whole honey bees: 3.0 ± 1.1; Whole bumble bees: 2.8 ± 0.9) (*p* < 0.05, Table S5; Table 1). There was no significiant difference in average number of AIs in pollen from conventional (HB 2018: 21.2 ± 1.1, HB 2019: 20.6 ± 0.7, BB 2019: 19.1 ± 0.5) or unsprayed (HB 2018: 21.6 ± 0.5, HB 2019: 20.5 ± 0.5, BB 2019: 17.8 ± 1.5) farms across both years and in both bee species, although samples from organic farms in 2018 did have significantly higher average number of AIs per sample (HB 2018: 26.0 ± 0.5)^38^ (*p* > 0.05, Tables S5, 1). There was also no significant difference in average number of AIs between wax samples from conventional (15.0 ± 0.5) and unsprayed farms (14.2 ± 0.8) (*p* > 0.05, Table S5; Table 1). Blueberry flowers and whole honey bees collected from conventional farms also had significantly higher pesticide concentrations (Flowers: 688.9 ppb ± 122.6 S.E.; Whole honey bees: 2579.6 ppb ± 687.0) than those from unsprayed farms (Flowers: 3.3 ppb ± 1.3; Whole honey bees: 218.9 ppb ± 151.2) (*p* < 0.05, Table S5; Table 1). Across both years and for both bee species, pollen collected from conventional farms also had significantly higher average concentrations of pesticides (HB 2018: 286.2 ± 34.1, HB 2019: 770.0 ± 139.3, BB 2019: 1708.2 ± 226.6) compared to samples from unsprayed farms (HB 2018: 85.8 ± 11.8, HB 2019: 287.3 ± 81.7, BB 2019: 330.3 ± 116.8)^38^ (*p* < 0.05, Tables S5, 1). This pattern was not seen in whole bumble bees or honey bee wax sampled from conventional (Whole bumble bees: 680.8 ppb ± 259.8; Wax: 525.6 ppb ± 141.3) compared to unsprayed farms (Whole bumble bees: 541.2 ppb ± 132.2; Wax: 709.9 ppb ± 412.2) (*p* > 0.05, Table S5; Table 1).

### Comparison of pesticide risk among sample types

**Figure 1:**
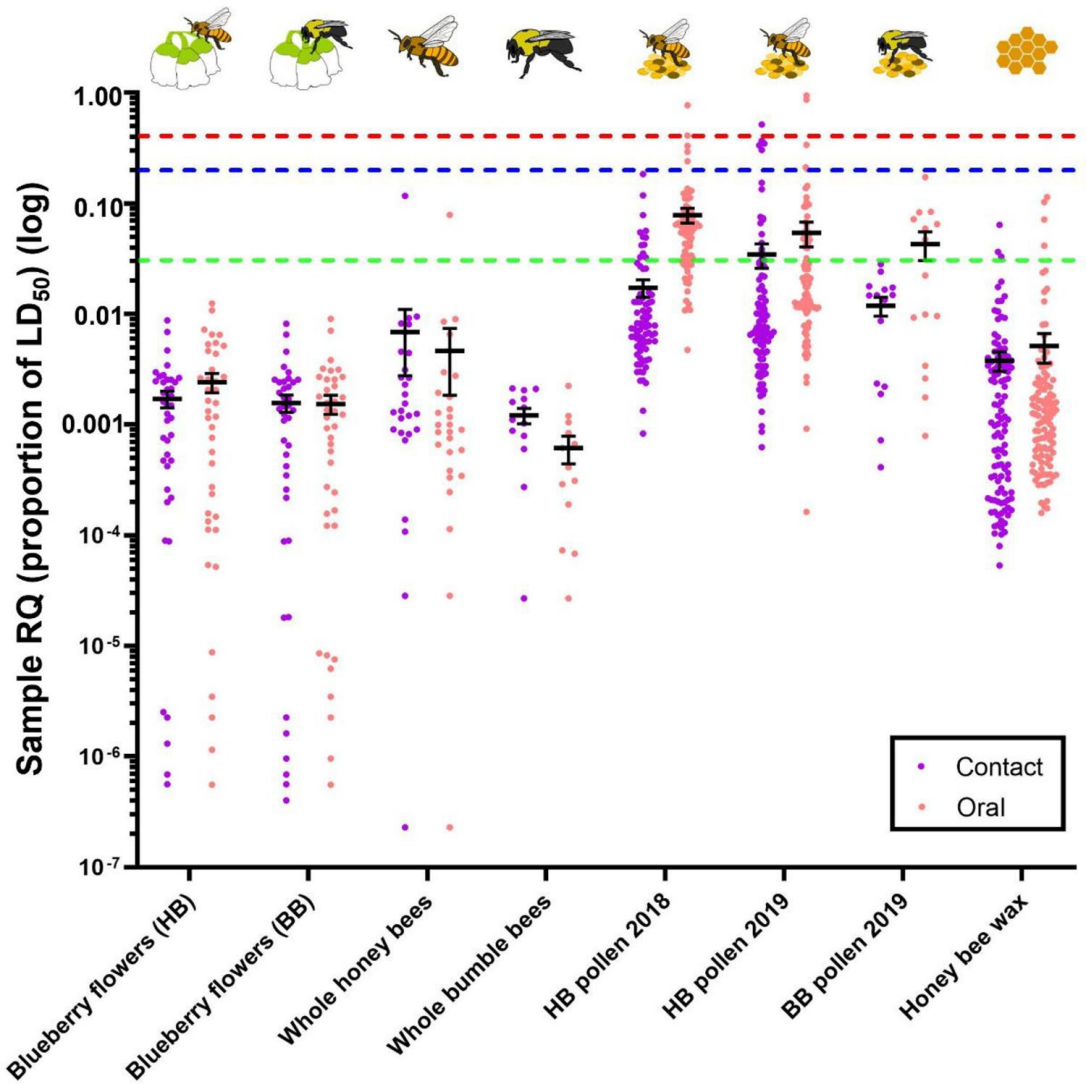
Average sample risk quotient by sample type. Risk quotient (RQ) was calculated with contact (purple) and oral (pink) LD_50_ values. Toxicity data for *Apis mellifera* and *Bombus terrestris* were used depending on the sample type. Individual sample RQs are represented by the dots. Horizontal black lines are the mean, and the error bars represent standard error of the mean. RQ is displayed in relation to the EPA and EFSA levels of concern (proportion of the LD50). The green dashed line is the EFSA level of concern for chronic exposure (relevant for oral toxicity). The blue dashed line is the EFSA level of concern for acute exposure, and the red dashed line is the EPA level of concern for acute exposure. Graph created in GraphPad Prism 9^48^.

Average sample RQ was significantly different among sample types (Contact Tox.: R^2^m = 0.34, R^2^c = 0.46; *X*^2^ = 240.5, df = 7, *p* < 0.001; Oral Tox.: R^2^m = 0.57, R^2^c = 0.63; *X*^2^ = 549.9, df = 7, *p* < 0.001) (Fig. 1). When using contact toxicity data, the 2 years of honey bee pollen samples and the bumble bee pollen samples from 2019 had similar average RQ (presented as the proportion of LD_50_) (HB pollen 2018: 0.017 ± 0.003 S.E.; HB pollen 2019: 0.035 ± 0.009; BB pollen 2019: 0.012 ± 0.002), and average RQ of these pollen samples was significantly higher than all other sample types (honey bee whole bees: 0.007 ± 0.004; honey bee wax: 0.004 ± 0.001; blueberry flowers (HB tox.): 0.002 ± 0.000; blueberry flowers (BB tox.): 0.002 ± 0.000; bumble bee whole bees: 0.001 ± 0.000) (Tukey’s < 0.05; Fig. 1). Whole honey bees and honey bee wax samples also had significantly higher sample RQs compared to blueberry flowers (Tukey’s: *p* < 0.05). When using oral toxicity data, the results were similar, though honey bee collected pollen in 2018 (0.079 ± 0.012) has significantly higher average sample RQ than honey bee pollen in 2019 (0.054 ± 0.014) (Tukey’s: *p* < 0.05) (Fig. 1).

Flower samples and whole honey bees collected from conventional farms had significantly higher average sample RQs than those collected from unsprayed farms (*p* < 0.05; Table S5). However, for whole bumble bees, pollen samples, and wax samples, there was no significant difference between conventional and unsprayed farms (*p* > 0.05; Table S5).

### Pesticide contributions to risk

**Figure 2:**
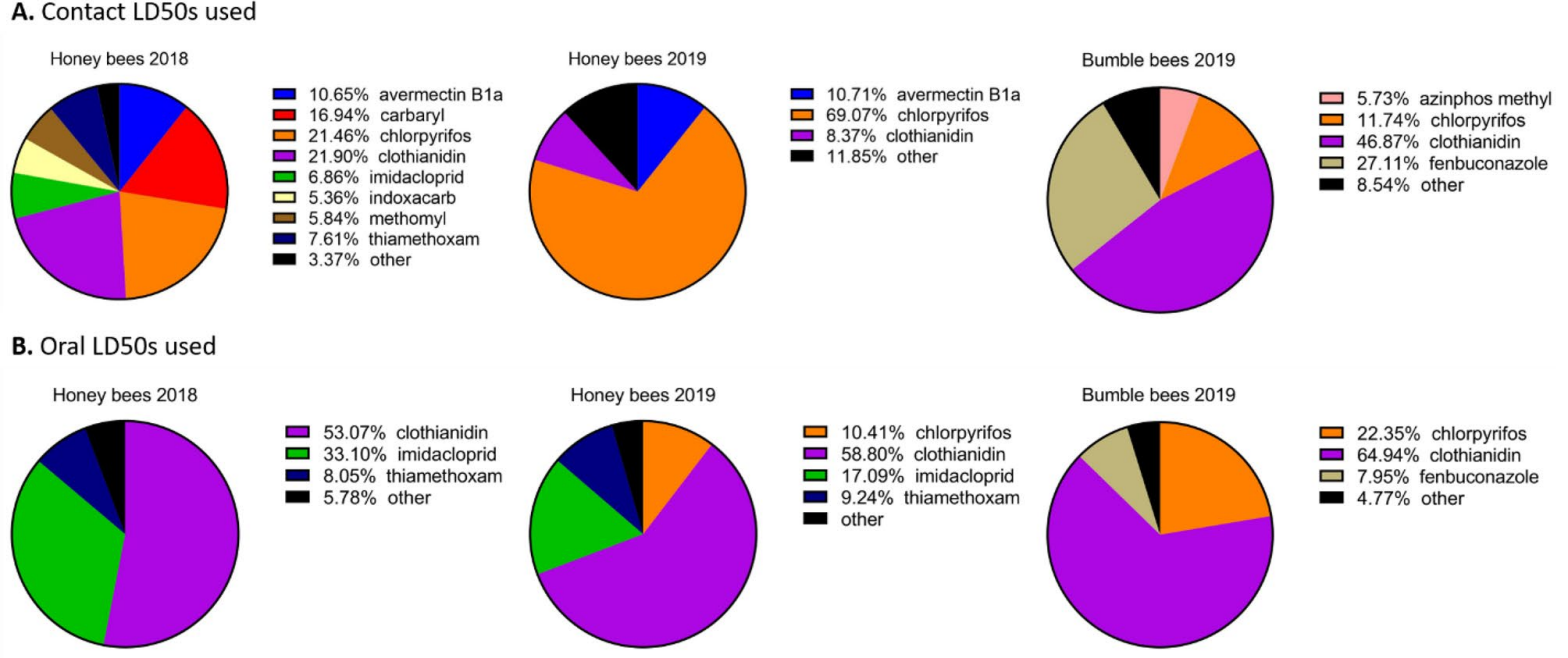
Contribution of individual active ingredients to the pollen risk quotient calculations for each bee species and year (2018/2019). This was determined both for RQs calculated with contact LD_50_ values (**A**) and oral LD_50_ values (**B**). Toxicity data for *Apis mellifera* and *Bombus terrestris* were used depending on the pollen source. Graph created in GraphPad Prism 9^48^.

The contribution of pesticides with widely different toxicities and concentrations to the overall pollen RQ varied by bee species and year (Figs. 2, S3). Contributions to RQ for flower samples were similar using honey bee or bumble bee toxicity data, with fenbuconazole contributing the most to the RQ (Honey bees: 63.6%, Bumble bees: 69.2%) (Fig. S3). For whole honey bee samples, carbaryl (63.8%) contributed the most to the overall RQ, while for whole bumble bees, carbendazim (43.2%) and fenbuconazole (33.9%) contributed the most (Fig. S3). For honey bee wax, chlorpyrifos (60.7%) contributed most to the overall RQ (Fig. S3).

We also determined the contribution of pesticides registered for use on blueberries (for applications any time of year) to the overall RQ of the different sample types. For blueberry flower samples, when using honey bee or bumble bee toxicity data, pesticides registered for use on blueberries attributed the most to the overall RQs (*Apis* toxicity: 89.1%; *Bombus* toxicity: 92.5%). This was also true for both whole honey bees (98.0%) and whole bumble bees (56.8%). However, for wax, pesticides not registered for use on blueberries at any time of year contributed the most to the RQ (87.3%).

For honey bee collected pollen, when contact toxicity data were used, chlorpyrifos, clothianidin, and carbaryl contributed most to RQ, though this varied by year (Fig. 2). Using oral toxicity data, clothianidin contributed most to RQ, followed by imidacloprid. For bumble bee collected pollen, when using oral toxicity data, clothianidin and fenbuconazole contributed most to RQ, compared with clothianidin and chlorpyrifos when using oral toxicity data (Fig. 2).

**Figure 3:**
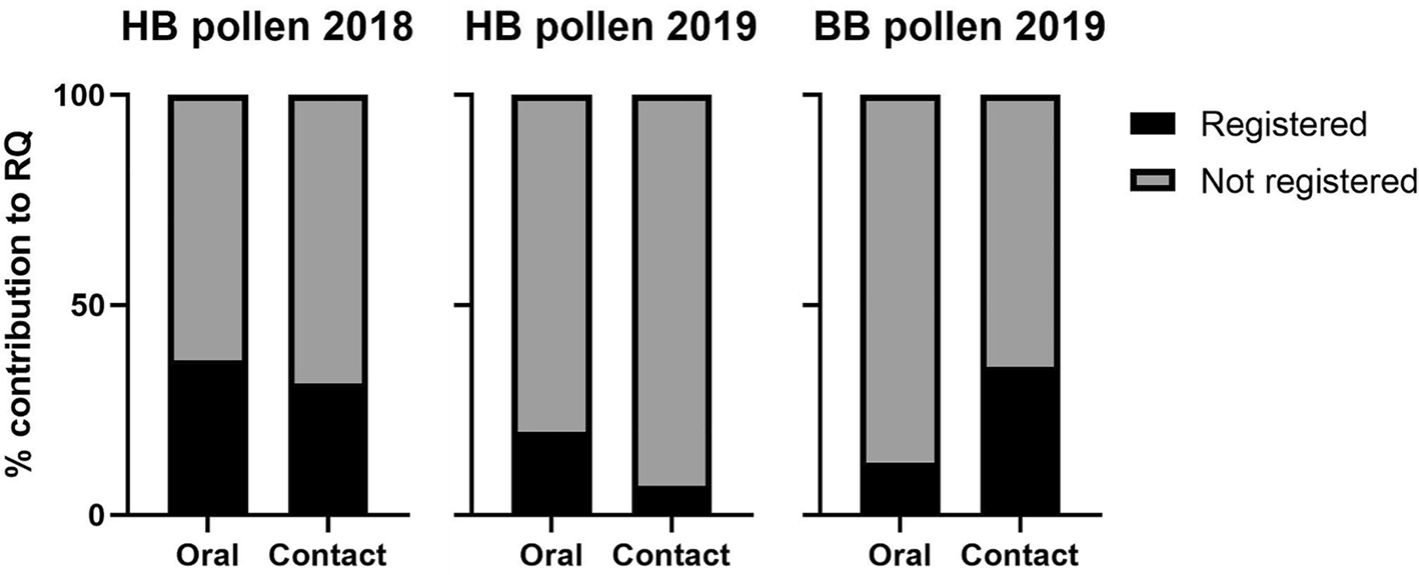
Contribution of pesticide active ingredients to pollen risk quotient values based on whether they are registered for use on blueberries, or not. A product was considered registered for use on blueberries if the label indicated it is permitted to be applied to blueberry bushes at any time of the year. RQs were calculated with contact LD_50_ values and oral LD_50_ values. Toxicity data for *Apis mellifera* and *Bombus terrestris* were used depending on which species collected the pollen (HB-honey bees, *Apis mellifera*, BB-bumble bees, *Bombus impatiens*). Graph created in GraphPad Prism 9^48^.

For all pollen samples (both years and species), and when using both oral and contact toxicity, the majority of the RQ came from pesticides not registered for use on blueberries at any time of the year (Fig. 3). For honey bee pollen collected in 2018, 68.7% of the RQ_contact_ and 63.2% of the RQ_oral_ were from products not registered for use on blueberries. For honey bee pollen collected in 2019, 93.0% of the RQ_contact_ and 80.2% of the RQ_oral_ were from products not registered for use on blueberries. For bumble bee pollen collected in 2019, 64.6% of the RQ_contact_ and 87.4% of the RQ_oral_ were from products not registered for use on blueberries.

### Risk assessment

Using contact toxicity data, no single pesticide detections in flower samples reached the EPA (0.4, or 40% of LD_50_) or EFSA (0.2, or 20% of LD_50_) acute risk levels of concern (LOC), for either honey bee or bumble bee toxicity. No whole bee samples reached these LOCs either. No pesticide detections in pollen samples reached the 0.4 EPA LOC, though five detections were above the 0.2 EFSA LOC. These included chlorpyrifos and avermectin B1a detections (Table 2). All these detections were from honey bee collected pollen in 2019. No wax samples reached these LOCs.

**Table 2:** Percent of samples with active ingredient detections above the EPA (0.4) and EFSA (0.2) acute risk thresholds. For a chronic risk threshold (oral toxicity only), the EFSA uses 0.03.

	Material	Year	Active ingredient, pesticide type (I = insecticide)	% of samples above EPA acute threshold (%)	% of samples above EFSA acute threshold (%)	% of samples above EFSA chronic threshold (oral tox. only) (%)	Highest conc. detected (ppb)	LD_50_ (ug per bee)	Prop. of LD_50_ for highest detection
Contact	Honey bee pollen, n = 97	2019	chlorpyrifos, I	0	4.1		213.5	0.059	0.36
			avermectin B1a, I	0	1.0		7.2	0.002	0.36
Oral	Honey bee pollen, n = 76	2018	clothianidin, I	1.3	3.9	31.6	27.3	0.004	0.68
			imidacloprid, I	0	1.3	26.3	8.7	0.0037	0.24
			thiamethoxam, I	0	0	5.3	4.2	0.005	0.08
	Honey bee pollen, n = 97	2019	clothianidin, I	2.1	2.1	22.7	35.7	0.004	0.89
			thiamethoxam, I	0	1.0	4.1	14.7	0.005	0.30
			imidacloprid, I	0	0	4.1	2.0	0.004	0.05
			chlorpyrifos, I	0	0	5.2	213.5	0.25	0.09
			avermectin B1a, I	0	0	1.0	7.18	0.009	0.08
			carbaryl, I	0	0	1.0	93.2	0.21	0.04
	Bumble bee pollen, n = 15	2019	clothianidin, I	0	0	46.7	3.2	0.004*	0.08
			chlorpyrifos, I	0	0	6.7	272.7	0.23	0.12

Both contact and oral LD_50_ values were used to calculate risk and are presented separately. N is the number of samples. Sample types without detections above thresholds are excluded. Asterisks (*) marks where honey bee toxicity data was used for bumble bee collected pollen.

When using oral toxicity data, again, no detections in flower samples reached the EPA (0.4) or EFSA (0.2) acute exposure LOC, or the EFSA chronic exposure LOC (0.03, or 3% of LD_50_) when using either honey bee or bumble bee toxicity values. The highest risk detections were from the fungicide fenbuconazole (highest: 444.7 ppb; RQ for HBs and BBs: 0.009). No whole bee samples reached the EPA or EFSA acute risk levels. Detections in honey bee collected pollen were above the EPA acute LOC (RQ > 0.4), all of which were from clothianidin and all from one farm (Farm 13, in 2018 and 2019). Additional detections were above the EFSA acute exposure LOC (RQ > 0.2) and included the neonicotinoid insecticides clothianidin, thiamethoxam, and imidacloprid (Table 2). Many more detections were above the EFSA chronic exposure LOC (RQ > 0.03), and included detections of clothianidin, imidacloprid, thiamethoxam, chlorpyrifos, avermectin B1a, and carbaryl (Table 2). For bumble bee collected pollen, no detections were above the EPA or EFSA acute exposure LOCs, though there were detections above the EFSA chronic exposure LOC, which included detections of clothianidin and chlorpyrifos (Table 2).

For acute contact exposure, no flower, whole bee, or wax samples (sum of individual detection RQs in a sample) were above the EPA or EFSA LOCs. However, when using oral toxicity data, 3.4% of whole honey bee sample and 3.5% of wax samples were above the EFSA chronic exposure LOC (Fig. 1). For bee collected pollen samples, when using contact toxicity data, 1.0% of honey bee pollen samples from 2019 were above the EPA acute LOC (RQ > 0.4), while 5.2% were above the EFSA acute LOC (RQ > 0.2) (Fig. 1). When using oral toxicity data, 2.6% of honey bee pollen samples in 2018 and 2.1% in 2019 were above the EPA acute LOC (RQ > 0.4), and all these samples were from the same farm (Farm 13). In 2018, 6.6% of honey bee pollen samples and 5.2% in 2019 were above the EFSA acute LOC (RQ > 0.2). Additionally, 72.4% of honey bee pollen samples in 2018 and 45.4% in 2019 were above the EFSA chronic LOC (RQ > 0.03), while 46.7% of bumble bee pollen samples exceeded this level (Fig. 1).

### Comparisons among farms

**Figure 4:**
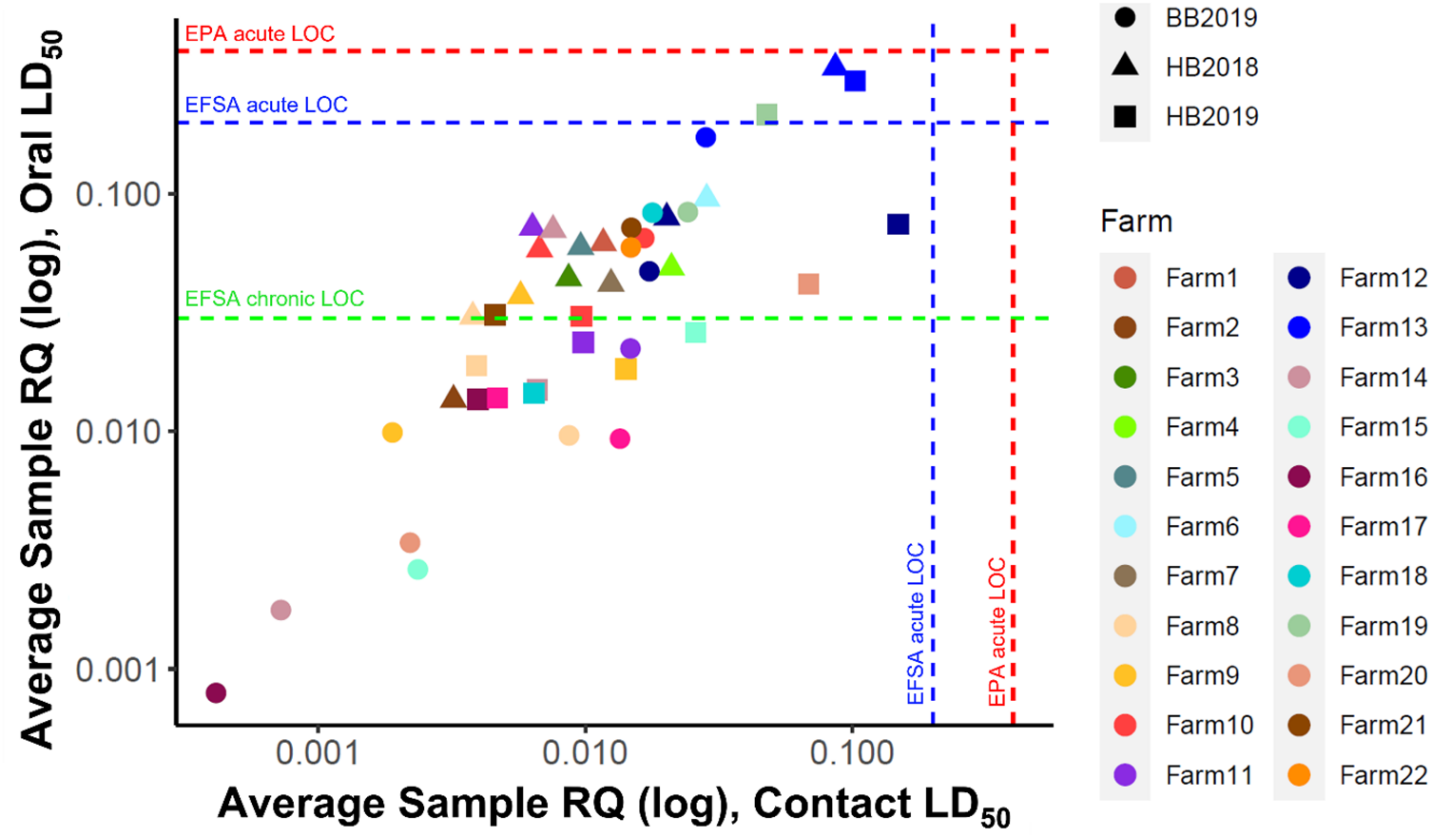
Average pollen sample risk quotient at each farm where pollen was collected from honey bees in 2018 (triangles) and 2019 (squares) and bumble bees in 2019 (circles). Contact (x-axis) and oral (y-axis) LD_50_ values were used to calculate risk quotient using *Apis mellifera* toxicity values for honey bee pollen, and *Bombus terrestris* values were used whenever possible for bumble bee pollen. RQ is displayed in relation to the EPA and EFSA levels of concern (proportion of the LD50). The green dashed line is the EFSA level of concern (LOC) for chronic oral exposure (0.03). The blue dashed line is the EFSA level of concern for acute exposure (0.2), and the red dashed line is the EPA level of concern for acute exposure (0.4). However, EPA and EFSA do not use acute LOCs for oral exposure. These are included on the y-axis for illustrative purposes only. Graph created in R version 4.1.1^47^ with the package ggplot2^110^.

No farms had an average sample RQ_contact_ above the EPA or EFSA acute contact LOCs (RQ > 0.4 or 0.2) (Fig. 4). However, when considering oral toxicity, one farm in 2018 and two farms in 2019 had average honey bee pollen sample RQs_oral_ above the EFSA acute LOC (RQ > 0.2). Additionally, in 2018, 92.9% of farms had average sample RQs_oral_ above the EFSA chronic LOC (RQ > 0.03) for honey bee pollen samples. In 2019, 42.9% of farms for honey bee pollen and 46.7% of farms for bumble bee pollen had average sample RQs_oral_ above the EFSA chronic LOC.

Relative risks from contact exposure to pesticides in pollen samples were not consistent over time at the same farms, with no significant correlation in average sample RQs_contact_ between years (Pearson’s r = 0.6, *p* = 0.15). There was also no correlation of risk from pollen collected from honey bees and pollen collected by bumble bees at the same sites in 2019 (Pearson’s r = 0.4, *p* = 0.15). However, relative risks from pollen through oral exposures (average sample RQs_oral_) were highly positively correlated across years (Pearson’s *r* = 0.98, *p* < 0.001) and between species at the same farms (Pearson’s *r* = 0.81, *p* < 0.001) (Fig. S4).

### Active ingredients causing high pollen sample RQ

Two farms had average pollen sample RQs_oral_ above the EFSA acute LOC (Farms 13 and 19; Fig. 4), and surprisingly both were unsprayed farms. For Farm 13, high sample RQ_oral_ for honey bee pollen in 2018 and 2019 was driven by detections of clothianidin (highest detection in 2018: 27.3 ppb, RQ_oral_: 0.68; highest in 2019: 35.7 ppb, RQ_oral_: 0.89). For Farm 19 (only sampled in 2019), high average honey bee pollen sample RQ_oral_ was driven by detections of thiamethoxam (highest: 14.7 ppb, RQ_oral_: 0.29). For bumble bee pollen, no farms had average sample RQ_oral_ above the acute LOCs, but Farm 13 also had the highest average sample RQ_oral_ from detections of chlorpyrifos (272.7 ppb; RQ_oral_: 0.12) and clothianidin (2.1 ppb; RQ_oral_: 0.05).

### Correlation between landscape features and pollen RQ

In both years and for both species, area of apple and cherry orchards in the surrounding landscape was positively correlated with pollen sample RQ (Table S6). For apple, this was significant for honey bee pollen collected in 2018 at the 1000 m (RQ_contact_: Pearson’s *r* = 0.81, *p* = 0.006; RQ_oral_: Pearson’s *r* = 0.78, *p* = 0.014) and 2000 m landscape scales (RQ_contact_: Pearson’s *r* = 0.89, *p* < 0.001; RQ_oral_: Pearson’s *r* = 0.85, *p* = 0.002). It was also significant for honey bee collected pollen in 2019 at the 2000 m landscape scale (RQ_oral_: Pearson’s *r* = 0.75, *p* = 0.033), and for bumble bee collected pollen in 2019 at the 500 m (RQ_oral_: Pearson’s *r* = 0.77, *p* = 0.022) and 2000 m landscape scales (RQ_oral_: Pearson’s *r* = 0.73, *p* = 0.025). There was also a significant positive correlation between area of cherry orchards and RQ for honey bee collected pollen in 2019 at the 500 m scale (RQ_contact_: Pearson’s *r* = 0.83, *p* = 0.004). Farm 13 had the greatest surrounding acreage of apple (2000 m: 6.65% of surrounding area) and cherry (2000 m: 1.84%) compared to other farms (apple range: 0.02–2.55%; cherry range: 0.04–1.32%), so to determine whether the correlation held for other farms, we repeated the analyses with this farm removed. The correlation coefficient was much lower, and only significant for cherry acreage for honey bee collected pollen in 2019 at the 500 m landscape scale (RQ_contact_: Pearson’s *r* = 0.91, *p* = 0.004) (Table S6). The proportion of surrounding landscape in blueberry fields was not correlated with sample RQ, for any of the spatial scales tested.

## Discussion

Our results highlight that pesticide risks to bees are primarily from pesticides that are not applied on the farms where bees are placed for crop pollination and these exposures are primarily through bee collected pollen. We found that pollen brought back to colonies had the most active ingredients per sample, followed by wax, flowers, and whole bees. Pollen also had higher average pesticide risk compared to the other sample types, more pesticide detections above the EPA and EFSA levels of concern, and these hazardous detections were found at a greater frequency. The higher pesticide diversity and risk in pollen reflects that bees collect pollen from diverse habitats, including other crops where they can contact high-risk pesticides. This highlights the potential for high-risk pesticide exposure to managed bee colonies situated in regions of diverse crop production, where some crops are in bloom and others have completed bloom thereby allowing applications of insecticides that can cause high-risk bee exposures.

As expected, sprayed blueberry fields had a greater number of residues in whole bee and blueberry flower samples collected from these farms than unsprayed farms. However, this effect of farm management was not found in pollen (honey bee or bumble bee) or wax samples, likely because samples collected directly from the farm (flowers and whole bees) are more representative of exposure in that field, whereas pollen is collected from the broad landscape, with blueberry pollen representing just a small subset of pollens that are brought back to the colony^38^. Wax is a lipophilic matrix that can collect pesticide residues over time, and honey bees may have been brought to multiple crops and been exposed to a diversity of pesticides before this study. Residues in wax are relatively stable across the season, due to the stability of pesticides in this matrix and low wax replacement rates^26,29^.

All sample types had higher pesticide concentrations when collected from conventional farms than unsprayed farms except for whole bumble bees and honey bee wax. High concentrations were primarily driven by pesticides used in blueberry pest management. The lack of a farm management effect on pesticide concentrations in whole bumble bee samples was unexpected, as bumble bees have previously been shown to have relatively high site fidelity^55^, though recent work has estimated that bumble bee patch fidelity is lower than honey bees^56^. It is also possible that sample sizes were too low, or that bumble bees interact with the flowers in ways that reduce exposure compared to honey bees. For instance, bumble bees collect proportionally more blueberry pollen compared to honey bees^38^, as bumble bees are able to extract the pollen from the poricidal anthers of blueberry flowers using buzz pollination^57^. Honey bees cannot perform this behavior and more often are visiting blueberry flowers to collect nectar, reaching their head and tongues deep into the corolla (Fig. S2). We have also commonly observed honey bees walking across flower clusters while collecting nectar, while bumble bees tend to fly between flowers during pollen collection (pers. obs.). These differences in behavior could explain the observed differences in residues in whole bee samples.

Even though pesticide concentrations were significantly higher in samples collected on conventional farms, this did not always translate to higher risk. Farm management only had a significant effect on average sample RQ for blueberry flowers and whole honey bees. For all other sample types (whole bumble bees, pollen samples, and wax) there was no effect of farm management on average sample RQ. Surprisingly, pollen and wax samples from unsprayed farms had similar risk to those from conventional farms, suggesting that the riskiest exposures detected in pollen and wax are from outside the focal blueberry fields, with pollen being the primarily source of risk to the colony. As expected, exposures and pesticide risk in flowers and whole honey bee samples are more representative of management in the focal field.

Chronic exposure to pesticides has the potential to impact the health of colonies after they leave farms for pollination, such as reducing honey production^58,59^. Reductions in honey crop can then translate to significant profit losses for beekeepers^60^. These potential impacts have relevancy to our results where over 90% of honey bee pollen samples were above the EFSA chronic LOC in 2018, and over 40% of pollen samples reached this threshold in 2019. Some active ingredients in particular drove high sample risk scores. Active ingredients with detections above the EFSA acute LOC (RQ_oral_ > 0.2) included avermectin B1a, clothianidin, imidacloprid, thiamethoxam, and chlorpyrifos. These are all insecticides not applied in blueberry fields during bloom. Fenbuconazole, a fungicide applied to blueberry bushes during bloom, also contributed to bumble bee pollen RQ, though no individual detections were above the acute or chronic LOCs. Because of their contributions to sample RQ, we discuss each individually below.

Fenbuconazole was detected at high concentrations across several sample substrates (blueberry flowers: 444.7 ppb; whole honey bees: 426.5 ppb; bumble bee collected pollen: 636.6 ppb), and at high frequencies of detection (flowers: 75% of samples; whole honey bees: 72%; bumble bee pollen: 100%). Consequently, it was a substantial contributor to sample RQ in the sampled matrices despite a relatively high LD_50_ (honey bee contact = 5.5 μg bee^−1^, oral = 5.2 μg bee^−1^). This triazole fungicide is used to control diseases such as anthracnose and mummy berry, and for disease control in apple, cherry, and other crops. It is typically applied to blueberry bushes during bloom and while alone this fungicide is not highly toxic to bees, it acts synergistically with other pesticides, increasing their risk. For example, it enhances neonicotinoid activity making imidacloprid and acetamiprid up to five times more toxic^61^, and can increase the lethality of pyrethroids synergistically through inhibition of cytochrome P450s^62^. This interaction has also been shown for in-hive acaracides, including coumaphos^62^, which was found in 100% of our wax samples, at up to 728.2 ppb. Triazole fungicides can also lower the amount of pollen collected by honey bees from cranberry after application^63^.

The ability of fungicides to synergize the effects of other detected pesticides highlights a limitation of the RQ as an estimate of risk in agricultural systems, because it does not account for interactions between multiple ingredients. Of the 383 samples we analyzed, all but five whole bee samples had more than one pesticide detected. There is growing evidence for synergistic effects of combined pesticide exposures^35,61,64–67^, indicating a need to integrate this into risk assessments^68^. Average pollen sample RQ_oral_ values were above the EFSA chronic LOC using the additive approach, so the RQ values reported here are likely an underestimate of the risk to managed bees.

Our results demonstrate an additional limitation of the RQ for quantifying risk to bees: it relies on toxicity to adult bees and does not account for sub-lethal effects, even those that could result in colony loss. Exposure of larvae to toxic pesticide residues can create the risk of cascading negative effects on the colony dynamics as foraging worker bees decline and a negative feedback loop can develop^69^. For this reason, there is an increasing effort to understand how pesticides and their mixtures might affect developing bees^70^, and to develop larva-specific LD_50_ values^71^. Sublethal impacts can be anticipated for many other AIs identified in our study. For instance, there is evidence that exposure to the fungicide Pristine, which is a combination of pyraclostrobin and boscalid, can inhibit mitochondrial function^72^, impair olfactory learning^73^, and reduce honey bee worker lifespan and colony population size^74^. This fungicide is used in blueberry fields during bloom to combat diseases and was detected in over 90% of bee collected pollen, over 75% of wax, over 50% of whole bees, and over 30% of flowers. High concentrations were also common across the different sample types (Table S2), including particularly high concentrations in whole honey bee samples (boscalid: 8794.9 ppb; pyraclostrobin: 2208.1 ppb). To fully understand the risks associated with pesticide exposure, measurements of sublethal impacts should be included with traditional toxicity studies.

Avermectin B1a has been shown to be highly toxic to honey bees (LD_50_contact: 0.002 bee^−1^, LD_50_oral: 0.009 bee^−1^). It was found in honey bee pollen at one farm during both years of our study, and its detection in 2019 was above the EFSA acute contact LOC (RQ_contact_ > 0.2). This was also a meaningful contributor to RQ for honey bee pollen collected in 2018 and 2019 when using contact toxicity. This insecticide/acaricide is used on various fruits and hops and other crops in the region (trade name: Agri-mek), including for control of plum curculio in apples as a mix with thiamethoxam (trade name: Agri-flex), but it is not registered for use on blueberries. Avermectin B1a is also used as a veterinary parasiticide in the control of nematodes and arthropods affecting livestock, and a possible route of exposure is through contaminated soils and surface water^75^. The primary crops around the site where it was found included apples and corn/soy, though the most abundant habitat type was natural area (63.08% at 2 km radius). Use of avermectin is restricted to before and after bloom in bee-attractive crops, and in apples its use is most common at petal fall, which could align in timing for when honey bees were at the nearby site for blueberry pollination. Avermectin B1a has also been detected in honey bee collected pollen during apple pollination in New York^25^. The detections of avermectin B1a highlight the importance of accounting for activities on other nearby farms and applications on other crops. They also show why pesticide exposure and risk studies should sample multiple sites to understand the range of potential risks to bees.

Three neonicotinoid insecticides, clothianidin, imidacloprid, and thiamethoxam, were found in pollen at levels above the EFSA acute LOC (RQ_oral_ > 0.2). These insecticides are used to control various sucking and chewing insects in a variety of crops, most commonly as seed treatments in corn and soybeans. Clothianidin is not registered for use on blueberries, thiamethoxam has limited use in blueberry farms in this region, and imidacloprid is used in some conventional blueberry farms in the region. Due to their high toxicity, all are restricted to use before or after bloom in bee attractive crops.

Clothianidin was only detected in pollen samples, including 82% of honey bee pollen samples in 2018, and 68% and 60% of honey bee and bumble bee pollen samples in 2019, respectively. The highest detection was in honey bee pollen from 2019, at 35.7 ppb (RQ_contact_ = 0.081 and RQ_oral_ = 0.893). Clothianidin was the only active ingredient with detections above the EPA acute LOC (RQ_oral_ > 0.4) (1.3% of honey bee pollen samples with detections > 0.4 in 2018 and 2.1% of honey bee pollen samples in 2019). Considering the EFSA chronic LOC (RQ_oral_ > 0.03) for clothianidin, we found that 31.6% of honey bee pollen samples in 2018, 22.7% of honey bee pollen samples in 2019, and 46.7% of bumble bee pollen samples in 2019 had detections above this level. Clothianidin was the primary contributor to RQ for honey bee pollen samples in 2018 (contact and oral toxicity), honey bee pollen samples in 2019 (oral toxicity only), and bumble bee pollen in 2019 (contact and oral toxicity). This pesticide can have sublethal effects on honey bees such as alterations in gene expression (e.g. down-regulation of members of the royal jelly proteins, and genes of toxicologically relevant pathways^76^), changes in dance communication^77^, memory impairment^78^, alterations in hygienic and foraging behavior^79^, and reductions in adult body weight^80^.

Imidacloprid was also detected in over 90% of pollen samples, and in 6.18% of wax samples. For honey bee pollen samples, 26.3% in 2018 and 4.1% in 2019 had detections above the EFSA chronic LOC (RQ_oral_ > 0.03). Imidacloprid was also a meaningful contributor to overall RQ for honey bee pollen collected in 2018 (contact and oral toxicity) and honey bee pollen in 2019 (oral toxicity only). Imidacloprid exposure has also been shown to impede homing and foraging activity in honey bees^81–83^, result in higher rates of queen failure and lower winter colony survival^84^, lower adult bee populations in the hive and reduce their ability to regulate temperature^85^, alter gene expression^86,87^, and have negative effects on neural development and neural cell damage^88,89^.

Thiamethoxam was also primarily detected in honey bee pollen (46% of samples in 2018 and 13% of samples in 2019), but only in 0.88% of wax samples. In honey bee collected pollen, 5.3% and 4.1% of samples had thiamethoxam detections above the EFSA chronic LOC (RQ_oral_ > 0.03) in 2018 and 2019, respectively. Thiamethoxam was a meaningful contributor to overall RQ in honey bee pollen collected in 2018 (contact and oral toxicity) and 2019 (oral toxicity only). Sublethal impacts on foraging^90,91^, gene expression^76,92,93^, organ development^94^ and increases in viral loads after exposure^95^ have all been documented in honey bees following exposure to thiamethoxam.

The source of these neonicotinoids could include bees foraging on treated crops or collecting from off-target sources such as weeds growing in crop fields. Post-bloom applications in apple and cherry orchards could also align with the timing of blueberry bloom, and these are systemic insecticides that can be transported into pollen and nectar following application^96,97^. Previous studies in Michigan also showed that collection of pollen from weeds associated with agricultural fields is correlated with an increase in thiamethoxam and clothianidin exposure for honey bees^98^. Use of mowing or pre-emergent herbicides to remove of weeds that provide a pollen/nectar source for bees near treated crops could help with reducing this risk. However, given the uncertainty of exposure sources, broad adoption of IPPM strategies including scouting, degree-day models, and use of reduced risk insecticides could reduce these exposures by reducing the need for applications.

Chlorpyrifos is not registered for use in blueberry fields, yet was found in 89% of pollen samples, 35% of wax samples, 15% of flower samples, and 3% of whole honey bees. It was the primary contributor to RQ for honey bee pollen samples in 2019 (contact toxicity), and a substantial contributor for honey bee pollen in 2018 (contact toxicity) and bumble bee pollen in 2019 (contact and oral toxicity). It was also the primary contributor to honey bee wax RQ_contact_. For honey bee pollen samples in 2019, 4.1% of samples had chlorpyrifos detections above the EFSA acute LOC (RQ_contact_ > 0.2). Additionally, 5.2% of honey bee pollen samples and 6.7% of bumble bee pollen samples in 2019 were above the EFSA chronic LOC (RQ_oral_ > 0.03). Chlorpyrifos is registered for trunk applications to control insects in vineyards and orchards, as well in corn and other field crops in the region. It has high contact toxicity to honey bees (LD_50_contact: 0.059 bee^−1^), with lower toxicity to bumble bees (LD_50_contact: 1.58 bee^−1^). However, oral toxicity is slightly higher in bumble bees (LD_50_oral: 0.23 bee^−1^) compared to honey bees (LD_50_oral: 0.25 bee^−1^). Chlorpyrifos can also have sublethal effects on honey bee olfactory-mediated memory^99^ and can cause larval mortality^70,71^. There is also some evidence of synergistic toxicity of chlorpyrifos and tetraconazole, lambda-cyhalothrin, and bifenthrin^66^. These pyrethroids were not screened for in the samples, though are commonly applied to vegetables, fruits, and field crops. Tetraconazole was screened for, but not detected in any samples.

Chlorpyrifos is prevalent across agricultural landscapes in the United States due to its high rate of usage across diverse cropping systems^24,100,101^. This has recently been banned on food crops by the U.S. Environmental Protection Agency^102^, which could reduce future risk to pollinators. Given the prevalence of chlorpyrifos in samples, this imminent ban could provide substantial reductions in risk for managed pollinators if exposure is primarily from current applications and not legacy residues.

The differences in risk values found for honey bees and bumble bees demonstrate the importance of species-specific data for estimation of RQ. The most widely available toxicity values for all types of pesticides are the contact and oral LD_50_ values for adult honey bees. While these provide a proxy for the relative risk for other bees, species vary widely in their sensitivity^61,103^, so including more bee species in toxicity studies will help further clarify these differences. The LOCs considered in this study were also developed for honey bees, so the relevance to bumble bees is less clear. This is especially true for chronic oral LOCs, as typical rates of pollen consumption by bumble bees are much less certain compared to honey bees^104–106^. The relevance of these thresholds for other substrate types (wax, flowers, whole bees) is also not known so although we used these thresholds to compare estimated risk between bee species and between substrate types, there is significant uncertainty in their suitability for non-*Apis* bees and for other substrate types.

One of the greatest utilities of calculating RQ values is for comparison to other published studies^19,20,24–26,29,101,107^. Chlorpyrifos is detected frequently in pollen samples leading to high detection RQs^19,20,24,26,29,101^, and thiamethoxam^24,25,101^, clothianidin^101,107^, and imidacloprid^19,20,101,107^ were also found at concentrations that led to high RQs in other studies. Additional insecticides were detected at low levels in this study despite causing high RQs in pollen samples in other studies including phosmet^19,24^ and carbaryl^24,25,101^. Their low contribution to risk in this system likely reflects the different use pattern across landscapes.

The results of this study point toward various potential risk mitigation strategies. Adoption of integrated disease management and selection of alternative fungicide options could reduce risk from fungicides labeled for use during bloom. For example, leaf wetness monitors have been used in blueberry fields in Michigan to predict anthracnose risk, a common fungal disease controlled by fenbuconazole^108^. Degree-day models can predict optimal periods for protection against fruitworms using methoxyfenozide, and *Bacillus thuringiensis* is an alternative with much lower risk to bees^109^. However, while risk mitigation actions on the farm where bees are placed will be important, our data suggest that changes at other farms within the flight distance of foraging bees will be needed too. We found correlations between abundance of other crops in the landscape and risk to bees, even though the proportion of land in those crops was relatively small. Additionally, most of the pesticides that posed the greatest risk to bees were not from pesticides sprayed on the farms where bees were located. Therefore, IPPM programs must consider the impacts to local bees outside of the crop bloom. More research is needed to identify exposure routes, as this will be critical for developing management strategies that can reduce exposure to the most hazardous pesticides collected by managed bee colonies, and their risk when pollinating in these farm settings.

## Supplementary Information

Supplementary Information.
