# Supplementary material for: Pesticide risk to managed bees during blueberry pollination is primarily driven by off-farm exposures
Source: Sci Rep. 2022 May 3;12:7189. doi: 10.1038/s41598-022-11156-1 (PMC9065077; doi:10.1038/s41598-022-11156-1)
Supplement: Supplementary file 1 — Supplementary Information. [file 41598_2022_11156_MOESM1_ESM.docx]

**SUPPLEMENTAL:** Pesticide risk to managed bees during blueberry pollination is primarily driven by off-farm exposures.

Kelsey K. Graham^1,3*^, Meghan O. Milbrath^1^, Yajun Zhang^1^, Nicolas Baert^2^, Scott McArt^2^, and Rufus Isaacs^1^

1. Department of Entomology, Michigan State University, 202 CIPS, 578 Wilson Road, East Lansing, MI 48824
2. Department of Entomology, Cornell University, 4129 Comstock Hall, Ithaca, NY 14853
3. Current Affiliation: U.S. Department of Agriculture – Agricultural Research Service, Pollinating Insect – Biology, Management, Systematics Research Unit, 1410 N. 800 E., Logan, UT 84341

***Corresponding author:** Kelsey K. Graham, [kelsey.katherine.graham@gmail.com](mailto:kelsey.katherine.graham@gmail.com), +1 435.797.3879

| **Table S1.** Spray records for applications made during bloom for all conventional and organic farms where sampling occurred in 2018 and 2019. When samples were collected is indicated in the last three columns. Only honey bee pollen was collected in 2018. Greyed cells indicate no samples were taken. Please note that applications were made in different sections of the farm on different days, but individual fields were not always noted on the records provided. Therefore, the same products may be applied on different days in different areas of the farm. Repeat applications that would be off-label were not made. | | | | | | | | | | |
| --- | --- | --- | --- | --- | --- | --- | --- | --- | --- | --- |
| **Farm** | **Production type** | **Year** | **Product applied** | **Class** | **Active ingredient** | **Date of application** | **Application rate (per acre)** | **Collection of honey bee pollen** | **Collection of bumble bee pollen, bumble bees, honey bees, & blueberry flowers** | **Collection of honey bee wax – *these collections were done in honey yards. Not on farms.*** |
| Farm1 | Conventional | 2018 | Luna Tranquility | fungicide | fluopyram, pyrimethanil | 21-May-18 | 16 fl oz | 29-May-18, 4-Jun-18 |  |  |
|  |  |  | Aframe | fungicide | azoxystrobin | 29-May-18 | 12.8 fl oz |  |  |  |
|  |  |  | Dipel | insecticide | Bacillus thuringiensis | 29-May-18 | 1 lb |  |  |  |
| Farm3 | Organic | 2018 | Magna-Bon CS2005 | fungicide | copper sulfate pentahydrate | 17-May-18 | 24 fl oz | 29-May-18, 6-Jun-18 |  |  |
|  |  |  | Avenger Ag Burndown | herbicide | d-Limonene | 17-May-18 | 5.6 pints |  |  |  |
|  |  |  | Timorex Gold | fungicide | tea tree oil | 24-May-18 | 14 fl oz |  |  |  |
|  |  |  | Axxe | herbicide | ammonium nonanoate | 28-May-18 | 2 gallon |  |  |  |
|  |  |  | Magna-Bon CS2005 | fungicide | copper sulfate pentahydrate | 4-Jun-18 | 24 fl oz |  |  |  |
|  |  |  | Dipel | insecticide | Bacillus thuringiensis | 4-Jun-18 | 1 lb |  |  |  |
| Farm4 | Conventional | 2018 | Callisto | herbicide | mesotrione | 30-Apr-18 | 6 oz | 29-May-18, 4-Jun-18 |  |  |
|  |  |  | Diuron 80 df | herbicide | diuron | 30-Apr-18 | 2 lb |  |  |  |
|  |  |  | Sinbar WDG | herbicide | terbacil | 30-Apr-18 | 1.5 lb |  |  |  |
|  |  |  | Gramoxone | herbicide | paraquat dichloride | 30-Apr-18 | 1 quarts |  |  |  |
|  |  |  | Ziram 76df | fungicide | ziram | 5-May-18 | 3 lb |  |  |  |
|  |  |  | Indar 2F | fungicide | fenbuconazole | 5-May-18 | 6 oz |  |  |  |
|  |  |  | Ziram 76df | fungicide | ziram | 15-May-18 | 3 lb |  |  |  |
|  |  |  | Indar 2F | fungicide | fenbuconazole | 15-May-18 | 6 oz |  |  |  |
|  |  |  | Pristine | fungicide | boscalid, pyraclostrobin | 22-May-18 | 18 oz |  |  |  |
|  |  |  | Abound flowable | fungicide | azoxystrobin | 31-May-18 | 10 oz |  |  |  |
|  |  |  | Intrepid 2F | insecticide | methoxyfenozide | 31-May-18 | 10 oz |  |  |  |
|  |  |  | Quash | fungicide | metconazole | 1-Jun-18 | 2.5 oz |  |  |  |
|  |  |  | Lannate LV | insecticide | methomyl | 1-Jun-18 | 3 pints |  |  |  |
| Farm5 | Organic | 2018 | Magna-Bon CS2005 | fungicide | copper sulfate pentahydrate | 16-May-18 | 24 fl oz | 29-May-18, 6-Jun-18 |  |  |
|  |  |  | Avenger Ag Burndown | herbicide | d-Limonene | 17-May-18 | 5.6 pints |  |  |  |
|  |  |  | Timorex Gold | fungicide | tea tree oil | 24-May-18 | 14 fl oz |  |  |  |
|  |  |  | Axxe | herbicide | ammonium nonanoate | 24-May-18 | 2 gallon |  |  |  |
|  |  |  | Magna-Bon CS2005 | fungicide | copper sulfate pentahydrate | 4-Jun-18 | 24 fl oz |  |  |  |
|  |  |  | Dipel | insecticide | Bacillus thuringiensis | 4-Jun-18 | 1 lb |  |  |  |
| Farm6 | Organic | 2018 | Magna-Bon CS2005 | fungicide | copper sulfate pentahydrate | 17-May-18 | 24 fl oz | 29-May-18, 6-Jun-18 |  |  |
|  |  |  | Avenger Ag Burndown | herbicide | d-Limonene | 17-May-18 | 5.6 pints |  |  |  |
|  |  |  | Axxe | herbicide | ammonium nonanoate | 19-May-18 | 2 gallon |  |  |  |
|  |  |  | Timorex Gold | fungicide | tea tree oil | 25-May-18 | 14 fl oz |  |  |  |
| Farm7 | Conventional | 2018 | Tilt | fungicide | propiconazole | 15-May-18 | 6 oz | 29-May-18, 6-Jun-18 |  |  |
|  |  |  | Ziram 76df | fungicide | ziram | 15-May-18 | 3 lb |  |  |  |
|  |  |  | Dual Magnum | herbicide | s-metolachlor | 22-May-18 | 1 pints |  |  |  |
|  |  |  | Vaquero | herbicide | clethodim | 22-May-18 | 8 fl oz |  |  |  |
|  |  |  | Quash | fungicide | metconazole | 23-May-18 | 2.5 oz |  |  |  |
|  |  |  | Aframe | fungicide | azoxystrobin | 1-Jun-18 | 12.8 fl oz |  |  |  |
|  |  |  | Dipel | insecticide | Bacillus thuringiensis | 1-Jun-18 | 1 lb |  |  |  |
| Farm8 | Conventional | 2018 | Sinbar WDG | herbicide | terbacil | 2-May-18 | 1.5 lb | 29-May-18, 5-Jun-18 |  |  |
|  |  |  | Diuron 80 df | herbicide | diuron | 2-May-18 | 3 lb |  |  |  |
|  |  |  | Callisto | herbicide | mesotrione | 2-May-18 | 6 fl oz |  |  |  |
|  |  |  | Indar 2F | fungicide | fenbuconazole | 7-May-18 | 6 fl oz |  |  |  |
|  |  |  | Captan Gold 80wdg | fungicide | captan | 7-May-18 | 2 lb |  |  |  |
|  |  |  | Indar 2F | fungicide | fenbuconazole | 16-May-18 | 6 fl oz |  |  |  |
|  |  |  | Captan Gold 80wdg | fungicide | captan | 16-May-18 | 2 lb |  |  |  |
|  |  |  | Pristine | fungicide | boscalid, pyraclostrobin | 23-May-18 | 20 fl oz |  |  |  |
|  |  | 2019 | Magna-Bon CS2005 | fungicide | copper sulfate pentahydrate | 24-Apr-19 | 8 oz | 24-May-19, 11-Jun-19 | 14-May-19, 23-May-19, 3-Jun-19, 6-Jun-19 | 27-Jun-19 |
|  |  |  | Indar 2F | fungicide | fenbuconazole | 10-May-19 | 6 oz |  |  |  |
|  |  |  | Indar 2F | fungicide | fenbuconazole | 26-May-19 | 6 oz |  |  |  |
| Farm10 | Conventional | 2018 | Quash | fungicide | metconazole | 17-May-18 | 1.5 fl oz | 29-May-18, 1-Jun-18 |  |  |
|  |  |  | Regalia | fungicide | Reynoutria sachalinensis | 28-May-18 | 1.5 quarts |  |  |  |
|  |  |  | Dipel | insecticide | Bacillus thuringiensis | 28-May-18 | 1 lb |  |  |  |
|  |  | 2019 | Alion | herbicide | indaziflam | 21-Mar-19 | 5 oz | 27-May-19, 30-May-19, 7-Jun-19 | 16-May-19, 22-May-19, 28-May-19, 4-Jun-19 | 26-Jun-19 |
|  |  |  | Magna-Bon CS2005 | fungicide | copper sulfate pentahydrate | 22-Apr-19 | 1.5 pt |  |  |  |
|  |  |  | Bravo Weather-Stik | fungicide | chlorothalonil | 3-May-19 | 2 qt |  |  |  |
|  |  |  | Propi-Star ec | fungicide | propiconazole | 3-May-19 | 6 oz |  |  |  |
|  |  |  | Indar 2F | fungicide | fenbuconazole | 14-May-19 | 6 oz |  |  |  |
|  |  |  | Luna Tranquility | fungicide | fluopyram, pyrimethanil | 26-May-19 | 24 oz |  |  |  |
|  |  |  | Proline 480 sc | fungicide | prothioconazole | 3-Jun-19 | 5.7 oz |  |  |  |
|  |  |  | Intrepid 2F | insecticide | methoxyfenozide | 3-Jun-19 | 14 oz |  |  |  |
| Farm11 | Conventional | 2018 | Quash | fungicide | metconazole | 17-May-18 | 1.5 fl oz | 29-May-18, 1-Jun-18 |  |  |
|  |  |  | Regalia | fungicide | Reynoutria sachalinensis | 28-May-18 | 1.5 quarts |  |  |  |
|  |  |  | Dipel | insecticide | Bacillus thuringiensis | 28-May-18 | 1 lb |  |  |  |
|  |  | 2019 | Magna-Bon CS2005 | fungicide | copper sulfate pentahydrate | 22-Apr-19 | 1.5 pt | 27-May-19, 30-May-19, 6-Jun-19 | 15-May-19, 18-May-19, 28-May-19, 5-Jun-19 | 24-Jun-19 |
|  |  |  | Aim EC | herbicide | carfentrazone-ethyl | 26-Apr-19 | 2 oz |  |  |  |
|  |  |  | Karmex df | herbicide | diuron | 26-Apr-19 | 4 lb |  |  |  |
|  |  |  | Callisto | herbicide | mesotrione | 26-Apr-19 | 6 oz |  |  |  |
|  |  |  | Bravo Weather-Stik | fungicide | chlorothalonil | 3-May-19 | 2 qt |  |  |  |
|  |  |  | Propi-Star ec | fungicide | propiconazole | 3-May-19 | 6 oz |  |  |  |
|  |  |  | Indar 2F | fungicide | fenbuconazole | 14-May-19 | 6 oz |  |  |  |
|  |  |  | Luna Tranquility | fungicide | fluopyram, pyrimethanil | 26-May-19 | 24 oz |  |  |  |
|  |  |  | Proline 480 sc | fungicide | prothioconazole | 4-Jun-19 | 5.7 oz |  |  |  |
|  |  |  | Intrepid 2F | insecticide | methoxyfenozide | 4-Jun-19 | 14 oz |  |  |  |
| Farm12 | Conventional | 2018 | Quash | fungicide | metconazole | 17-May-18 | 1.5 fl oz | 29-May-18, 1-Jun-18 |  |  |
|  |  |  | Regalia | fungicide | Reynoutria sachalinensis | 28-May-18 | 1.5 quarts |  |  |  |
|  |  |  | Dipel | insecticide | Bacillus thuringiensis | 28-May-18 | 1 lb |  |  |  |
|  |  | 2019 | Bravo Weather-Stik | fungicide | chlorothalonil | 3-May-19 | 2 qt | 27-May-19, 30-May-19, 6-Jun-19 | 15-May-19, 18-May-19, 28-May-19, 4-Jun-19 | 21-Jun-19 |
|  |  |  | Propi-Star ec | fungicide | propiconazole | 3-May-19 | 6 oz |  |  |  |
|  |  |  | Indar 2F | fungicide | fenbuconazole | 14-May-19 | 6 oz |  |  |  |
|  |  |  | Luna Tranquility | fungicide | fluopyram, pyrimethanil | 26-May-19 | 24 oz |  |  |  |
|  |  |  | Proline 480 sc | fungicide | prothioconazole | 3-Jun-19 | 5.7 oz |  |  |  |
|  |  |  | Intrepid 2F | insecticide | methoxyfenozide | 3-Jun-19 | 14 oz |  |  |  |
| Farm15 | Conventional | 2019 | Magna-Bon CS2005 | fungicide | copper sulfate pentahydrate | 25-Apr-19 | 8 oz | 27-May-19, 30-Jun-19, 10-Jun-19 | 15-May-19, 24-May-19, 2-Jun-19, 6-Jun-19 | 25-Jun-19, 26-Jun-19 |
|  |  |  | Indar 2F | fungicide | fenbuconazole | 28-Apr-19 | 6 oz |  |  |  |
|  |  |  | Indar 2F | fungicide | fenbuconazole | 5-May-19 | 6 oz |  |  |  |
|  |  |  | Pristine | fungicide | boscalid, pyraclostrobin | 30-May-19 | 20 oz |  |  |  |
| Farm17 | Conventional | 2019 | Magna-Bon CS2005 | fungicide | copper sulfate pentahydrate | 25-Apr-19 | 8 oz | 24-May-19, 30-May-19, 10-Jun-19 | 14-May-19, 23-May-19, 2-Jun-19, 6-Jun-19 | 26-Jun-19, 27-Jun-19 |
|  |  |  | Indar 2F | fungicide | fenbuconazole | 14-May-19 | 6 oz |  |  |  |
|  |  |  | Pristine | fungicide | boscalid, pyraclostrobin | 30-May-19 | 20 oz |  |  |  |
| Farm18 | Conventional | 2019 | Magna-Bon CS2005 | fungicide | copper sulfate pentahydrate | 21-Apr-19 | 8 oz | 30-May-19, 12-Jun-19 | 15-May-19, 23-May-19, 31-May-19, 4-Jun-19 |  |
|  |  |  | Indar 2F | fungicide | fenbuconazole | 5-May-19 | 6 oz |  |  |  |
|  |  |  | Pristine | fungicide | boscalid, pyraclostrobin | 26-May-19 | 20 oz |  |  |  |
|  |  |  | Intrepid 2F | insecticide | methoxyfenozide | 8-Jun-19 | 12 oz |  |  |  |
| Farm20 | Conventional | 2019 | Magna-Bon CS2005 | fungicide | copper sulfate pentahydrate | 24-Apr-19 | 8 oz | 24-May-19, 30-May-19, 10-Jun-19 | 14-May-19, 22-May-19, 2-Jun-19, 6-Jun-19 | 24-Jun-19, 25-Jun-19, 26-Jun-19, 27-Jun-19 |
|  |  |  | Indar 2F | fungicide | fenbuconazole | 14-May-19 | 6 oz |  |  |  |
|  |  |  | Pristine | fungicide | boscalid, pyraclostrobin | 30-May-19 | 20 oz |  |  |  |
| Farm21 | Conventional | 2019 | Magna-Bon CS2005 | fungicide | copper sulfate pentahydrate | 21-Apr-19 | 8 oz | 24-May-19, 30-May-19, 11-Jun-19 | 14-May-19, 23-May-19, 31-May-19, 4-Jun-19 | 24-Jun-19, 25-Jun-19 |
|  |  |  | Indar 2F | fungicide | fenbuconazole | 6-May-19 | 6 oz |  |  |  |
|  |  |  | Pristine | fungicide | boscalid, pyraclostrobin | 29-May-19 | 20 oz |  |  |  |
|  |  |  | Intrepid 2F | insecticide | methoxyfenozide | 11-Jun-19 | 12 oz |  |  |  |
| Farm22 | Conventional | 2019 | Magna-Bon CS2005 | fungicide | copper sulfate pentahydrate | 25-Apr-19 | 8 oz |  | 15-May-19, 27-May-19, 2-Jun-19, 6-Jun-19 |  |
|  |  |  | Indar 2F | fungicide | fenbuconazole | 11-May-19 | 6 oz |  |  |  |
|  |  |  | Pristine | fungicide | boscalid, pyraclostrobin | 29-May-19 | 20 oz |  |  |  |

**Table S2.** Retention times and optimized SRM acquisition parameters for pesticides and internal standards (RT: Retention time, CE: Collision Energy).

| **Compound** | **RT (min)** | **Polarity** | **Precursor (m/z)** | **RF Lens (V)** | **Quantifying ion (m/z)** | **CE 1 (V)** | **Confirmation ion (m/z)** | **CE 2 (V)** |
| --- | --- | --- | --- | --- | --- | --- | --- | --- |
| d_7_-Propamocarb | 3.25 | Positive | 196.2 | 97 | 103.1 | 18 | 151.2 | 14 |
| d_4_-Imidacloprid | 5.05 | Positive | 260.1 | 114 | 213.1 | 16 | 179.1 | 19 |
| d_7_-Bentazone | 6.42 | Negative | 246.0 | 134 | 132.1 | 26 | 182.2 | 20 |
| d_5_-Atrazine | 7.42 | Positive | 221.0 | 113 | 179.1 | 18 | 101.0 | 25 |
| d_10_-Chlorpyrifos | 9.66 | Positive | 359.9 | 123 | 199.0 | 199 | 98.9 | 31 |
|  |  |  |  |  |  |  |  |  |
| Chlormequat chloride | 0.73 | Positive | 122.0 | 113 | 58.0 | 27 | 63.0 | 21 |
| Mepiquat chloride | 0.79 | Positive | 114.1 | 128 | 98.0 | 26 | 58.0 | 25 |
| Methamidophos | 1.79 | Positive | 141.9 | 100 | 94.0 | 14 | 125.0 | 14 |
| Cyromazine | 2.40 | Positive | 167.0 | 133 | 85.1 | 19 | 125.1 | 18 |
| Acephate | 2.74 | Positive | 184.0 | 65 | 143.0 | 10 | 94.8 | 23 |
| Omethoate | 3.24 | Positive | 214.0 | 115 | 182.8 | 10 | 124.9 | 18 |
| Propamocarb | 3.28 | Positive | 189.1 | 98 | 102.0 | 17 | 74.1 | 25 |
| Aminocarb | 3.32 | Positive | 209.1 | 124 | 137.1 | 24 | 152.1 | 14 |
| Formetanate hydrochloride | 3.35 | Positive | 222.1 | 144 | 165.1 | 15 | 120 | 27 |
| Butocarboxim sulfoxide | 3.47 | Positive | 207.0 | 94 | 132.0 | 10 | 88.0 | 10 |
| Pymetrozine | 3.57 | Positive | 218.0 | 150 | 105.0 | 20 | 78.1 | 39 |
| Dinotefuran | 3.59 | Positive | 203.0 | 98 | 113.1 | 10 | 129.1 | 12 |
| Butoxycarboxim | 3.65 | Positive | 223.0 | 124 | 166.1 | 15 | 46.1 | 26 |
| Aldicarb sulfone | 3.71 | Positive | 223.1 | 117 | 148.0 | 10 | 86.1 | 16 |
| Oxamyl | 3.90 | Positive | 237.0 | 73 | 72.1 | 10 | 90.0 | 10 |
| Methomyl | 4.13 | Positive | 163.1 | 71 | 87.9 | 10 | 106.1 | 10 |
| Demeton-S-methylsulfone | 4.25 | Positive | 263.0 | 150 | 169.0 | 16 | 108.9 | 28 |
| Thiamethoxam | 4.39 | Positive | 292.0 | 121 | 211.1 | 12 | 181.0 | 22 |
| Carbendazim | 4.42 | Positive | 192.0 | 103 | 160.1 | 18 | 132.0 | 30 |
| Mexacarbate | 4.50 | Positive | 223.1 | 136 | 151.1 | 24 | 166.1 | 15 |
| Monocrotophos | 4.54 | Positive | 224.0 | 112 | 127.0 | 16 | 192.9 | 10 |
| Ethiofencarb sulfone | 4.73 | Positive | 258.0 | 119 | 107.0 | 16 | 201.0 | 10 |
| Dicrotophos | 4.81 | Positive | 238.0 | 127 | 127.0 | 18 | 192.5 | 10 |
| Pirimicarb-desmethyl | 4.84 | Positive | 225.2 | 135 | 168.0 | 15 | 72.0 | 21 |
| Ethiofencarb sulfoxide | 4.91 | Positive | 242.0 | 106 | 107.0 | 18 | 185.0 | 10 |
| Trichlorfon | 4.94 | Positive | 256.9 | 131 | 108.9 | 18 | 79.0 | 30 |
| Clothianidin | 5.03 | Positive | 250.0 | 104 | 169.0 | 13 | 131.9 | 17 |
| Imidacloprid | 5.06 | Positive | 256.0 | 131 | 209.0 | 16 | 175.1 | 19 |
| Fenuron | 5.13 | Positive | 164.8 | 116 | 72.1 | 15 | 46.1 | 15 |
| Thiabendazole | 5.13 | Positive | 202.0 | 208 | 175.0 | 26 | 131.0 | 33 |
| Flumetsulam | 5.17 | Positive | 326.0 | 192 | 129.0 | 26 | 262.1 | 19 |
| Dimethoate | 5.19 | Positive | 229.8 | 106 | 198.8 | 10 | 124.9 | 22 |
| 3-Hydroxy-carbofuran | 5.21 | Positive | 238.1 | 119 | 181.0 | 10 | 163.1 | 16 |
| Vamidothion | 5.25 | Positive | 288.0 | 120 | 146.0 | 14 | 118.1 | 23 |
| Fuberidazole | 5.26 | Positive | 185.1 | 152 | 157.1 | 22 | 129.0 | 35 |
| Mevinphos | 5.27/5.83 | Positive | 225.0 | 97 | 127.0 | 17 | 192.9 | 10 |
| Metamitron | 5.29 | Positive | 203.0 | 170 | 174.8 | 17 | 104.0 | 23 |
| Methiocarb sulfoxide | 5.29 | Positive | 242.0 | 134 | 185.0 | 14 | 122.1 | 29 |
| Chloridazon | 5.41 | Positive | 222.0 | 152 | 104.0 | 23 | 92.0 | 26 |
| Acetamiprid | 5.55 | Positive | 223.0 | 118 | 126.0 | 21 | 90.0 | 34 |
| Methiocarb sulfone | 5.62 | Positive | 258.0 | 119 | 122.0 | 19 | 201.0 | 10 |
| Schradan | 5.69 | Positive | 287.1 | 144 | 242.1 | 14 | 135.1 | 26 |
| Ethirimol | 5.89 | Positive | 209.8 | 192 | 140.1 | 22 | 98.0 | 27 |
| Florasulam | 5.96 | Positive | 360.0 | 179 | 129.0 | 25 | 108.9 | 53 |
| Pirimicarb | 5.97 | Positive | 239.1 | 147 | 182.1 | 16 | 72.0 | 21 |
| Thiacloprid | 5.98 | Positive | 253.0 | 162 | 126.0 | 21 | 90.0 | 36 |
| Metoxuron | 6.21 | Positive | 229.0 | 145 | 72.1 | 18 | 156.0 | 26 |
| Formothion | 6.23 | Positive | 258.0 | 80 | 199.0 | 10 | 124.9 | 22 |
| Imazethapyr | 6.29 | Positive | 290.1 | 189 | 177.0 | 27 | 248.1 | 19 |
| Carbetamide | 6.33 | Positive | 237.1 | 102 | 192.0 | 10 | 120.0 | 16 |
| Metolcarb | 6.34 | Positive | 166.0 | 83 | 108.9 | 10 | 94.0 | 31 |
| Oxadixyl | 6.35 | Positive | 279.1 | 120 | 219.1 | 10 | 132.1 | 31 |
| Tricyclazole | 6.45 | Positive | 190.0 | 178 | 163.0 | 23 | 136.0 | 29 |
| Bentazone | 6.46 | Negative | 238.9 | 169 | 132.0 | 26 | 197.0 | 21 |
| Cyanazine | 6.46 | Positive | 241.1 | 164 | 214.1 | 18 | 103.9 | 29 |
| Azamethiphos | 6.56 | Positive | 324.9 | 166 | 182.9 | 16 | 111.9 | 34 |
| Bromacil | 6.60 | Positive | 261.0 | 112 | 204.9 | 14 | 187.8 | 28 |
| Propoxur | 6.60 | Positive | 210.0 | 87 | 111.0 | 14 | 168.1 | 10 |
| Thiophanate-methyl | 6.62 | Positive | 343.0 | 161 | 151.0 | 20 | 93.0 | 46 |
| Bendiocarb | 6.67 | Positive | 224.0 | 104 | 167.1 | 10 | 108.9 | 18 |
| Carbofuran | 6.68 | Positive | 222.0 | 111 | 165.1 | 12 | 123.0 | 22 |
| Ofurace | 6.76 | Positive | 282.1 | 145 | 254.1 | 12 | 160.1 | 24 |
| Malaoxon | 6.79 | Positive | 315.0 | 133 | 98.9 | 23 | 269.0 | 10 |
| Imazaquin | 6.81 | Positive | 312.1 | 195 | 267.1 | 21 | 199.0 | 28 |
| Thidiazuron | 6.83 | Positive | 220.6 | 119 | 101.9 | 16 | 127.9 | 17 |
| Pyroxsulam | 6.83 | Positive | 435.0 | 262 | 195.1 | 26 | 258.0 | 22 |
| Simetryn | 6.83 | Positive | 214.1 | 161 | 124.1 | 20 | 96.0 | 25 |
| Desmetryn | 6.85 | Positive | 214.1 | 161 | 172.0 | 18 | 82.0 | 30 |
| Ancymidol | 6.86 | Positive | 257.1 | 157 | 135.0 | 25 | 81.1 | 25 |
| Hexazinone | 6.89 | Positive | 253.1 | 141 | 171.1 | 16 | 71.1 | 31 |
| Tebuthiuron | 6.90 | Positive | 229.0 | 145 | 172.1 | 18 | 116.0 | 27 |
| Metosulam | 6.99 | Positive | 418.0 | 233 | 174.9 | 27 | 140.0 | 50 |
| Prometon | 7.08 | Positive | 226.1 | 156 | 184.1 | 19 | 142.1 | 23 |
| Carbaryl | 7.08 | Positive | 202.0 | 95 | 145.1 | 10 | 127.0 | 29 |
| Fenthion sulfoxide | 7.09 | Positive | 295.0 | 187 | 280.0 | 19 | 108.9 | 32 |
| Ethiofencarb | 7.09 | Positive | 226.1 | 105 | 107.0 | 17 | 164.1 | 10 |
| Cyantraniliprole | 7.10 | Positive | 475.0 | 158 | 285.9 | 11 | 444.0 | 18 |
| Terbumeton | 7.20 | Positive | 226.2 | 153 | 170.0 | 17 | 142.1 | 23 |
| Monolinuron | 7.20 | Positive | 215.0 | 131 | 126.0 | 18 | 148.0 | 15 |
| Fosthiazate | 7.21 | Positive | 284.0 | 118 | 103.9 | 21 | 228.0 | 10 |
| Fluometuron | 7.24 | Positive | 233.0 | 145 | 72.0 | 19 | 46 | 18 |
| 2,4-D | 7.27 | Negative | 218.9 | 101 | 160.9 | 13 | 125.0 | 26 |
| Bromoxynil | 7.29 | Negative | 275.8 | 194 | 80.9 | 31 | 78.9 | 30 |
| DNOC | 7.30 | Negative | 197.0 | 147 | 180.0 | 19 | 137.0 | 18 |
| Ethoxyquin | 7.36 | Positive | 218.1 | 183 | 160.1 | 33 | 148.1 | 22 |
| Benodanil | 7.36 | Positive | 323.8 | 180 | 231.0 | 23 | 202.9 | 35 |
| Imazalil | 7.37 | Positive | 297.0 | 170 | 156.0 | 23 | 200.9 | 18 |
| Isoprocarb | 7.38 | Positive | 194.1 | 105 | 95.0 | 15 | 137.1 | 10 |
| Flutriafol | 7.42 | Positive | 302.0 | 144 | 70.0 | 19 | 123.0 | 28 |
| Chlorotoluron | 7.43 | Positive | 213.0 | 141 | 72.1 | 18 | 46.0 | 16 |
| Atrazine | 7.44 | Positive | 216.1 | 167 | 174.0 | 18 | 103.9 | 28 |
| Metobromuron | 7.47 | Positive | 258.9 | 114 | 148.0 | 15 | 169.9 | 19 |
| Metazachlor | 7.48 | Positive | 278.0 | 111 | 210.1 | 10 | 134.1 | 22 |
| Lenacil | 7.50 | Positive | 235.1 | 109 | 153.1 | 16 | 136.0 | 32 |
| Isocarbophos | 7.53 | Positive | 307.0 | 73 | 231.0 | 16 | 273.0 | 10 |
| Metalxyl | 7.54 | Positive | 280.1 | 98 | 220.0 | 14 | 192.2 | 18 |
| Griseofulvin | 7.54 | Positive | 353.2 | 188 | 285.0 | 18 | 165.1 | 20 |
| Methoprotryne | 7.59 | Positive | 272.1 | 175 | 240.2 | 19 | 198.0 | 23 |
| Isoproturon | 7.59 | Positive | 207.1 | 143 | 72.1 | 19 | 165.1 | 14 |
| Fensulfothion | 7.64 | Positive | 309.0 | 180 | 280.9 | 15 | 253.0 | 18 |
| Heptenophos | 7.69 | Positive | 251.0 | 123 | 127.0 | 17 | 124.9 | 13 |
| Desmedipham | 7.71 | Positive | 301.3 | 133 | 182.0 | 10 | 136.0 | 20 |
| Forchlorfenuron | 7.71 | Positive | 248.0 | 134 | 129.0 | 18 | 93.0 | 33 |
| Dodemorph | 7.73 | Positive | 282.2 | 188 | 116.1 | 21 | 98.0 | 27 |
| Cycluron | 7.73 | Positive | 199.1 | 137 | 72.1 | 22 | 69.1 | 21 |
| Chlorantraniliprole | 7.75 | Positive | 481.9 | 182 | 283.9 | 12 | 450.8 | 18 |
| Methabenzthiazuron | 7.75 | Positive | 222.0 | 118 | 165.1 | 17 | 150.0 | 33 |
| Diuron | 7.77 | Positive | 233.0 | 145 | 72.1 | 19 | 46 | 18 |
| Ioxynil | 7.79 | Negative | 369.7 | 204 | 126.8 | 35 | 214.9 | 32 |
| Azaconazole | 7.82 | Positive | 299.9 | 162 | 159.0 | 28 | 231.0 | 17 |
| Phenmedipham | 7.82 | Positive | 301.1 | 145 | 168.0 | 10 | 136.0 | 20 |
| Dimefuron | 7.83 | Positive | 339.0 | 220 | 167.0 | 22 | 72.1 | 26 |
| Benoxacor | 7.84 | Positive | 260.0 | 173 | 149.1 | 18 | 134.0 | 29 |
| Clomazone | 7.91 | Positive | 240.0 | 134 | 125.0 | 21 | 89.0 | 47 |
| Diethofencarb | 7.92 | Positive | 268.0 | 114 | 226.1 | 10 | 124.0 | 32 |
| Azinphos-methyl | 7.93 | Positive | 317.9 | 103 | 132.0 | 15 | 125.0 | 17 |
| Fenobucarb | 7.93 | Positive | 208.1 | 110 | 95.0 | 15 | 152.0 | 10 |
| Ethofumesate | 7.98 | Positive | 287.1 | 159 | 121.0 | 16 | 259.1 | 10 |
| Fluazifop | 8.01 | Positive | 328.0 | 174 | 282.0 | 19 | 254.0 | 26 |
| Azoxystrobin | 8.03 | Positive | 404.1 | 175 | 372.0 | 14 | 344.1 | 25 |
| Propazine | 8.03 | Positive | 230.1 | 177 | 146.1 | 23 | 188.1 | 18 |
| Pyrimethanil | 8.03 | Positive | 200.1 | 184 | 107.0 | 25 | 168.1 | 30 |
| Nuarimol | 8.04 | Positive | 315.1 | 177 | 252.1 | 22 | 243.0 | 25 |
| Ethiprole | 8.04 | Positive | 396.9 | 189 | 350.9 | 21 | 255.0 | 36 |
| Fenamidone | 8.05 | Positive | 312.1 | 151 | 236.1 | 15 | 92.0 | 25 |
| Halofenozide | 8.07 | Positive | 331.0 | 99 | 275.1 | 10 | 105.0 | 18 |
| Dimethenamid | 8.09 | Positive | 276.1 | 135 | 244.1 | 14 | 168.1 | 24 |
| Prometryn | 8.11 | Positive | 242.2 | 149 | 158.0 | 24 | 200.0 | 19 |
| Methiocarb | 8.13 | Positive | 226.1 | 105 | 169.1 | 10 | 121.0 | 19 |
| Spiroxamine | 8.18 | Positive | 298.2 | 167 | 144.2 | 20 | 100.0 | 30 |
| Crotoxyphos | 8.18 | Positive | 332.0 | 100 | 210.9 | 10 | 127.0 | 25 |
| Mandipropamid | 8.18 | Positive | 412.1 | 186 | 328.1 | 15 | 356.1 | 10 |
| Terbuthylazine | 8.19 | Positive | 230.1 | 140 | 174.0 | 17 | 132.0 | 25 |
| Boscalid | 8.20 | Positive | 343.0 | 174 | 307.0 | 21 | 272.0 | 30 |
| Isoxaben | 8.20 | Positive | 333.2 | 167 | 164.9 | 19 | 150.0 | 39 |
| Promecarb | 8.21 | Positive | 208.1 | 107 | 109.0 | 16 | 151.1 | 10 |
| Paclobutrazol | 8.22 | Positive | 294.0 | 151 | 70.1 | 21 | 125.0 | 38 |
| Terbutryn | 8.23 | Positive | 242.1 | 158 | 186.0 | 19 | 91.0 | 28 |
| Fluopicolide | 8.23 | Positive | 382.9 | 193 | 172.9 | 23 | 144.9 | 48 |
| Propyzamide | 8.26 | Positive | 256.0 | 110 | 190.0 | 14 | 173.0 | 29 |
| Mepronil | 8.27 | Positive | 270.1 | 159 | 118.9 | 24 | 228.1 | 15 |
| Fluxapyroxad | 8.28 | Positive | 382.0 | 140 | 362.1 | 13 | 342.1 | 20 |
| Fludioxonil | 8.28 | Negative | 247.0 | 149 | 180.0 | 28 | 126.1 | 31 |
| Isoprothiolane | 8.32 | Positive | 291.1 | 116 | 231.0 | 10 | 188.8 | 22 |
| Methoxyfenozide | 8.32 | Positive | 369.2 | 113 | 149.1 | 17 | 313.1 | 10 |
| Dimethomorph | 8.33 | Positive | 388.1 | 225 | 301.0 | 21 | 165.1 | 32 |
| Triadimefon | 8.34 | Positive | 294.0 | 138 | 197.0 | 16 | 141.0 | 22 |
| Propetamphos | 8.34 | Positive | 282.0 | 106 | 138.0 | 17 | 156.0 | 10 |
| Myclobutanil | 8.39 | Positive | 289.0 | 121 | 70.1 | 18 | 124.9 | 33 |
| Fluorochloridone | 8.40 | Positive | 312.0 | 132 | 292.0 | 21 | 145.0 | 48 |
| Butafenacil | 8.42 | Positive | 492.1 | 170 | 331.0 | 24 | 349.0 | 15 |
| Cumyluron | 8.43 | Positive | 303.1 | 137 | 184.9 | 13 | 125.0 | 33 |
| Fluopyram | 8.43 | Positive | 397.0 | 202 | 208.0 | 22 | 173.0 | 29 |
| Iprovalicarb | 8.44 | Positive | 321.2 | 129 | 119.1 | 19 | 116.1 | 20 |
| Fenhexamid | 8.45 | Positive | 302.0 | 166 | 97.0 | 23 | 55.0 | 35 |
| Bifenazate | 8.46 | Positive | 301.1 | 119 | 198.0 | 10 | 170.1 | 19 |
| Fluoxastrobin | 8.49 | Positive | 459.1 | 219 | 427.0 | 17 | 188.0 | 35 |
| Triazophos | 8.49 | Positive | 314.0 | 164 | 162.1 | 19 | 119.0 | 34 |
| Bromuconazole | 8.49/8.90 | Positive | 377.8 | 185 | 159.0 | 30 | 161.0 | 31 |
| Mefenacet | 8.49 | Positive | 299.0 | 132 | 148.1 | 14 | 120.0 | 25 |
| Spirotetramat | 8.50 | Positive | 374.1 | 185 | 302.1 | 17 | 330.2 | 15 |
| Bupirimate | 8.50 | Positive | 317.1 | 204 | 166.1 | 21 | 272.1 | 20 |
| Fluquinoconazole | 8.50 | Positive | 375.9 | 130 | 349.0 | 19 | 307.1 | 26 |
| Flufenacet | 8.51 | Positive | 364.0 | 126 | 194.0 | 10 | 152.1 | 19 |
| Tepraloxydim | 8.52 | Positive | 342.1 | 153 | 250.2 | 13 | 166.1 | 21 |
| Simeconazole | 8.53 | Positive | 294.1 | 150 | 70.1 | 20 | 135.1 | 21 |
| Chloroxuron | 8.54 | Positive | 291.0 | 178 | 72.1 | 21 | 46.0 | 19 |
| Tetraconazole | 8.56 | Positive | 372.0 | 187 | 158.9 | 30 | 123.0 | 55 |
| Dimethametryn | 8.58 | Positive | 256.1 | 180 | 185.9 | 21 | 96.0 | 30 |
| Trietazine | 8.58 | Positive | 230.1 | 178 | 132.0 | 22 | 104.0 | 29 |
| Cyazofamid | 8.63 | Positive | 325.0 | 122 | 107.9 | 14 | 217.0 | 18 |
| Napropamide | 8.65 | Positive | 272.0 | 149 | 171.1 | 19 | 199.0 | 13 |
| Alachlor | 8.64 | Positive | 270.1 | 112 | 238.1 | 10 | 162.2 | 20 |
| Metolachlor | 8.64 | Positive | 284.1 | 143 | 252.1 | 15 | 176.1 | 26 |
| Fipronil | 8.65 | Negative | 434.9 | 138 | 330.0 | 15 | 250.0 | 26 |
| Epoxiconazole | 8.68 | Positive | 330.0 | 149 | 121.0 | 21 | 100.9 | 44 |
| Fenbuconazole | 8.70 | Positive | 337.1 | 188 | 125.0 | 31 | 70.1 | 21 |
| Fenamiphos | 8.72 | Positive | 304.1 | 180 | 217.0 | 18 | 201.9 | 35 |
| Haloxyfop | 8.72 | Positive | 361.8 | 166 | 316.0 | 18 | 91.0 | 30 |
| Picoxystrobin | 8.72 | Positive | 368.1 | 111 | 145.1 | 21 | 205.0 | 10 |
| Tebufenozide | 8.73 | Positive | 353.2 | 110 | 297.1 | 10 | 133.1 | 19 |
| Triadimenol | 8.73 | Positive | 297.0 | 101 | 133.0 | 14 | 105.0 | 39 |
| Flubendiamide | 8.74 | Positive | 683.0 | 195 | 407.9 | 10 | 274.0 | 30 |
| Rotenone | 8.74 | Positive | 395.1 | 231 | 213.1 | 23 | 192.1 | 24 |
| Fenoxycarb | 8.75 | Positive | 302.0 | 152 | 88.0 | 19 | 116.0 | 10 |
| Flusilazole | 8.76 | Positive | 316.1 | 192 | 247.1 | 18 | 165.1 | 27 |
| Carfentrazone-ethyl | 8.76 | Positive | 412.0 | 217 | 346.0 | 23 | 365.9 | 18 |
| Diflubenzuron | 8.76 | Positive | 311.0 | 131 | 158.0 | 13 | 141.1 | 32 |
| Dimoxystrobin | 8.77 | Positive | 327.2 | 108 | 204.9 | 10 | 115.9 | 22 |
| Phenthoate | 8.78 | Positive | 320.8 | 119 | 247.1 | 12 | 135.0 | 20 |
| Isoxadifen-ethyl | 8.79 | Positive | 296.1 | 146 | 232.1 | 17 | 263.2 | 10 |
| Kresoxim-methyl | 8.79 | Positive | 314.1 | 116 | 267.1 | 10 | 222.1 | 13 |
| Neburon | 8.80 | Positive | 275.0 | 171 | 88.1 | 16 | 57.0 | 21 |
| Sulfotep | 8.81 | Positive | 323.0 | 145 | 171.0 | 14 | 114.9 | 29 |
| Penthiopyrad | 8.84 | Positive | 360.1 | 149 | 276.0 | 14 | 256.1 | 20 |
| Fipronil sulfone | 8.86 | Negative | 450.8 | 152 | 415.0 | 15 | 282.0 | 26 |
| Tebuconazole | 8.87 | Positive | 308.0 | 162 | 70.1 | 23 | 125.0 | 38 |
| Cyprodynil | 8.88 | Positive | 226.1 | 153 | 93.0 | 34 | 108.1 | 26 |
| Anilofos | 8.89 | Positive | 368.0 | 173 | 198.9 | 14 | 124.9 | 31 |
| Carpropamid | 8.90 | Positive | 334.0 | 126 | 139.0 | 20 | 195.9 | 13 |
| Etrimfos | 8.90 | Positive | 293.0 | 178 | 265.1 | 17 | 124.9 | 26 |
| Chlorfenvinphos | 8.93 | Positive | 359.0 | 159 | 155.1 | 13 | 169.9 | 39 |
| Penconazole | 8.93 | Positive | 284.1 | 134 | 159.0 | 30 | 70.1 | 18 |
| Zoxamide | 8.95 | Positive | 336.0 | 167 | 186.9 | 22 | 159.0 | 39 |
| Benzoylprop-ethyl | 8.95 | Positive | 366.0 | 134 | 105.0 | 16 | 77.1 | 48 |
| Fenthion | 8.96 | Positive | 279.0 | 133 | 169.0 | 16 | 247.0 | 13 |
| Cyflufenamid | 8.99 | Positive | 413.0 | 161 | 295.0 | 15 | 241.1 | 23 |
| Propiconazole | 8.99 | Positive | 342.1 | 69 | 159.0 | 30 | 122.9 | 55 |
| Pirimiphos-methyl | 9.00 | Positive | 306.1 | 199 | 164.1 | 22 | 108.0 | 31 |
| Coumaphos | 9.01 | Positive | 362.9 | 169 | 227.0 | 26 | 306.8 | 18 |
| Hexaconazole | 9.01 | Positive | 314.0 | 151 | 70.0 | 21 | 159.0 | 32 |
| Metconazole | 9.03 | Positive | 320.1 | 170 | 70.1 | 24 | 125.0 | 38 |
| Phoxim | 9.04 | Positive | 299.0 | 95 | 129.0 | 11 | 77.0 | 30 |
| Pyraclostrobin | 9.04 | Positive | 387.8 | 159 | 194.0 | 12 | 163.1 | 24 |
| Benzoximate | 9.06 | Positive | 364.1 | 105 | 199.0 | 10 | 105.0 | 24 |
| Prochloraz | 9.07 | Positive | 376.0 | 125 | 307.9 | 10 | 266.0 | 17 |
| Spinosad (Spinosyn A) | 9.09 | Positive | 732.4 | 299 | 142.1 | 29 | 98.1 | 45 |
| Metrafenone | 9.10 | Positive | 409.0 | 169 | 209.1 | 14 | 227.0 | 21 |
| Pencycuron | 9.12 | Positive | 329.1 | 190 | 125.0 | 40 | 218.1 | 16 |
| Haloxyfop-methyl | 9.15 | Positive | 376.0 | 180 | 315.8 | 17 | 91.0 | 31 |
| Thiobencarb | 9.15 | Positive | 258.0 | 118 | 125.0 | 20 | 89.0 | 49 |
| Indoxacarb | 9.16 | Positive | 528.0 | 233 | 203.0 | 38 | 150.0 | 24 |
| Diniconazole | 9.16 | Positive | 326.0 | 178 | 70.0 | 26 | 159.1 | 31 |
| Trifloxystrobin | 9.18 | Positive | 409.0 | 179 | 186.0 | 18 | 145.0 | 44 |
| Piperophos | 9.19 | Positive | 354.1 | 175 | 171.0 | 22 | 255.0 | 14 |
| Difenoconazole | 9.24 | Positive | 406.0 | 214 | 251.0 | 26 | 337.0 | 18 |
| Dithiopyr | 9.24 | Positive | 402.0 | 167 | 354.0 | 18 | 272.0 | 29 |
| Cycloate | 9.25 | Positive | 216.0 | 126 | 154.1 | 12 | 83.1 | 16 |
| Hexaflumuron | 9.28 | Negative | 458.8 | 142 | 438.9 | 10 | 175.0 | 36 |
| Clethodim | 9.27 | Positive | 360.1 | 140 | 164.1 | 18 | 268.1 | 12 |
| Prosulfocarb | 9.34 | Positive | 252.1 | 139 | 91.0 | 22 | 128.1 | 13 |
| Triflumizole | 9.34 | Positive | 346.0 | 113 | 278.1 | 10 | 73.1 | 17 |
| Furathiocarb | 9.38 | Positive | 383.1 | 176 | 194.9 | 18 | 252.1 | 13 |
| Quizalofop-ethyl | 9.39 | Positive | 373.0 | 221 | 299.0 | 19 | 271.1 | 26 |
| Buprofezin | 9.40 | Positive | 306.1 | 129 | 201.1 | 12 | 116.0 | 16 |
| Profenophos | 9.41 | Positive | 374.9 | 171 | 304.8 | 19 | 346.9 | 13 |
| Tetramethrin | 9.41 | Positive | 332.2 | 139 | 164.1 | 24 | 135.1 | 18 |
| Sethoxydim | 9.43 | Positive | 328.2 | 153 | 178.0 | 19 | 282.1 | 12 |
| Fluazinam | 9.44 | Negative | 462.8 | 194 | 415.9 | 19 | 398.0 | 16 |
| Tebufenpyrad | 9.44 | Positive | 334.2 | 206 | 117.0 | 36 | 145.0 | 27 |
| Esprocarb | 9.47 | Positive | 266.2 | 139 | 91.0 | 24 | 71.1 | 15 |
| Piperonyl butoxide | 9.51 | Positive | 356.3 | 118 | 177.1 | 10 | 119.0 | 33 |
| Tolfenpyrad | 9.58 | Positive | 384.1 | 188 | 197.0 | 25 | 196.0 | 19 |
| Imibenconazole | 9.54 | Positive | 411.0 | 209 | 125.0 | 31 | 171.0 | 10 |
| Hexythiazox | 9.63 | Positive | 353.1 | 123 | 228.0 | 15 | 168.0 | 25 |
| Tralkoxydim | 9.64 | Positive | 330.2 | 159 | 284.2 | 13 | 138.1 | 20 |
| Chlorpyrifos | 9.66 | Positive | 349.9 | 126 | 197.9 | 19 | 321.7 | 11 |
| Spiromesifen | 9.68 | Positive | 371.1 | 132 | 273.2 | 10 | 255.2 | 23 |
| Flufenoxuron | 9.69 | Positive | 489.2 | 142 | 158.1 | 18 | 140.9 | 41 |
| Sulprofos | 9.69 | Positive | 323.0 | 118 | 218.9 | 16 | 247.0 | 12 |
| Etoxazole | 9.74 | Positive | 360.1 | 201 | 141.0 | 31 | 304.0 | 18 |
| Quinoxyfen | 9.81 | Positive | 308.0 | 234 | 196.9 | 32 | 162.0 | 45 |
| Chlorfluazuron | 9.82 | Positive | 541.8 | 223 | 384.9 | 21 | 158.0 | 20 |
| Difenacoum | 9.84 | Positive | 445.1 | 236 | 179.0 | 31 | 257.2 | 20 |
| Amitraz | 9.87 | Positive | 294.1 | 103 | 163.1 | 14 | 122.1 | 29 |
| Fenpyroximate | 9.88 | Positive | 422.2 | 197 | 366.2 | 16 | 214.1 | 30 |
| Avermectin B1a | 9.95 | Positive | 890.5 | 225 | 305.1 | 25 | 567.2 | 14 |
| Resmethrin | 10.03 | Positive | 339.1 | 158 | 171.1 | 15 | 128.0 | 41 |
| Brodifacoum | 10.12 | Positive | 523.1 | 289 | 335.0 | 22 | 178.0 | 34 |
| Fenazaquin | 10.24 | Positive | 307.1 | 159 | 161.2 | 17 | 57.1 | 23 |
| Etofenprox | 10.25 | Positive | 394.0 | 133 | 177.1 | 15 | 359.2 | 10 |

**Table S3.** Limits of Detection (LOD), Limits of Quantification (LOQ) and Upper Limits of Linearity (ULOL) for each sample type (information for pollen samples is available in (Graham et al. 2021).

|  | **Whole bees** | | | **Blueberry flowers** | | | **Wax** | | |
| --- | --- | --- | --- | --- | --- | --- | --- | --- | --- |
| **Compound** | **LOD (ng/sample)** | **LOQ (ng/sample)** | **ULOL (ng/sample)** | **LOD (ppb for 5 g of sample)** | **LOQ (ppb for 5 g of sample)** | **ULOL (ppb for 5 g of sample)** | **LOD (ng/sample)** | **LOQ (ng/sample)** | **ULOL (ng/sample)** |
| 2,4-D | 1400.0 | 4200.0 | 1750 | 140.00 | 420.00 | 1400 | 1000.0 | 3000.0 | 10000 |
| Carbofuran-3-hydroxy | 7.0 | 21.0 | 1750 |  |  |  |  |  |  |
| Acephate | 7.0 | 21.0 | 7000 | 1.40 | 4.20 | 1400 | 10.0 | 30.0 | 10000 |
| Acetamiprid | 0.8 | 2.5 | 1750 | 0.08 | 0.25 | 350 | 0.6 | 1.8 | 2500 |
| Alachlor | 3.5 | 10.5 | 7000 | 0.70 | 2.10 | 1400 | 5.0 | 15.0 | 10000 |
| Aldicarb sulfone | 21.0 | 63.0 | 7000 | 1.40 | 4.20 | 1400 | 10.0 | 30.0 | 10000 |
| Aminocarb | 0.7 | 2.1 | 7000 | 0.08 | 0.25 | 1400 | 0.6 | 1.8 | 10000 |
| Amitraz | 1.4 | 4.2 | 1750 | 0.28 | 0.84 | 1400 | 2.0 | 6.0 | 10000 |
| Ancymidol | 7.0 | 21.0 | 1750 | 1.40 | 4.20 | 350 | 10.0 | 30.0 | 2500 |
| Anilofos | 3.5 | 10.5 | 7000 | 0.28 | 0.84 | 1400 | 2.0 | 6.0 | 10000 |
| Atrazine | 3.5 | 10.5 | 7000 | 0.21 | 0.63 | 1400 | 1.5 | 4.5 | 10000 |
| Avermectin B1a | 56.0 | 168.0 | 1750 | 1.40 | 4.20 | 1400 | 10.0 | 30.0 | 10000 |
| Azaconazole | 1.8 | 5.3 | 7000 | 0.08 | 0.25 | 350 | 0.6 | 1.8 | 2500 |
| Azamethiphos | 1.8 | 5.3 | 1750 | 0.08 | 0.25 | 350 | 0.6 | 1.8 | 2500 |
| Azinphos-methyl | 14.0 | 42.0 | 7000 | 1.40 | 4.20 | 1400 | 10.0 | 30.0 | 10000 |
| Azoxystrobin | 1.8 | 5.3 | 1750 | 0.08 | 0.25 | 350 | 0.6 | 1.8 | 2500 |
| Bendiocarb | 7.0 | 21.0 | 1750 | 0.42 | 1.26 | 350 | 3.0 | 9.0 | 2500 |
| Benodanil | 1.4 | 4.2 | 7000 | 0.08 | 0.25 | 1400 | 0.6 | 1.8 | 10000 |
| Benoxacor | 7.0 | 21.0 | 7000 | 0.42 | 1.26 | 1400 | 3.0 | 9.0 | 10000 |
| Bentazone | 7.0 | 21.0 | 438 | 0.70 | 2.10 | 350 | 5.0 | 15.0 | 2500 |
| Benzoximate | 1.8 | 5.3 | 1750 | 0.28 | 0.84 | 350 | 2.0 | 6.0 | 2500 |
| Benzoylprop-ethyl | 1.8 | 5.3 | 7000 | 0.14 | 0.42 | 1400 | 1.0 | 3.0 | 10000 |
| Bifenazate | 1.4 | 4.2 | 1750 | 0.14 | 0.42 | 350 | 1.0 | 3.0 | 2500 |
| Boscalid | 2.8 | 8.4 | 1750 | 0.14 | 0.42 | 350 | 1.0 | 3.0 | 2500 |
| Brodifacoum | 14.0 | 42.0 | 1750 | 0.42 | 1.26 | 1400 | 3.0 | 9.0 | 10000 |
| Bromacil | 14.0 | 42.0 | 1750 | 0.42 | 1.26 | 350 | 3.0 | 9.0 | 2500 |
| Bromoxynil | 14.0 | 420.0 | 7000 | 4.20 | 12.60 | 350 | 30.0 | 90.0 | 2500 |
| Bromuconazole I | 7.0 | 21.0 | 7000 | 0.28 | 0.84 | 350 | 2.0 | 6.0 | 2500 |
| Bromuconazole II |  |  |  | 0.70 | 2.10 | 1400 | 5.0 | 15.0 | 10000 |
| Bupirimate | 2.1 | 6.3 | 7000 | 0.14 | 0.42 | 350 | 1.0 | 3.0 | 2500 |
| Buprofezin | 0.7 | 2.1 | 1750 | 0.08 | 0.25 | 350 | 0.6 | 1.8 | 2500 |
| Butafenacil | 1.4 | 4.2 | 7000 | 0.08 | 0.25 | 1400 | 0.6 | 1.8 | 10000 |
| Butocarboxim sulfoxide | 28.0 | 84.0 | 7000 | 1.40 | 4.20 | 1400 | 10.0 | 30.0 | 10000 |
| Butoxycarboxim | 21.0 | 63.0 | 7000 | 1.40 | 4.20 | 1400 | 10.0 | 30.0 | 10000 |
| Carbaryl | 10.5 | 31.5 | 7000 | 0.28 | 0.84 | 1400 | 2.0 | 6.0 | 10000 |
| Carbendazim | 7.0 | 21.0 | 7000 | 7.00 | 21.00 | 1400 | 50.0 | 150.0 | 10000 |
| Carbetamide | 3.5 | 10.5 | 1750 | 0.14 | 0.42 | 350 | 1.0 | 3.0 | 2500 |
| Carbofuran | 0.4 | 1.1 | 1750 | 0.03 | 0.08 | 350 | 0.2 | 0.6 | 2500 |
| Carbofuran-3-hydroxy |  |  |  | 0.28 | 0.84 | 350 | 2.0 | 6.0 | 2500 |
| Carfentrazone-ethyl | 14.0 | 42.0 | 7000 | 0.70 | 2.10 | 1400 | 5.0 | 15.0 | 10000 |
| Carpropamid | 1.4 | 4.2 | 7000 | 0.28 | 0.84 | 350 | 2.0 | 6.0 | 2500 |
| Chlorantraniliprole | 5.6 | 16.8 | 1750 | 0.14 | 0.42 | 1400 | 1.0 | 3.0 | 10000 |
| Chlorfenvinphos | 1.8 | 5.3 | 1750 | 0.14 | 0.42 | 350 | 1.0 | 3.0 | 2500 |
| Chlorfluazuron | 14.0 | 42.0 | 7000 | 1.40 | 4.20 | 1400 | 10.0 | 30.0 | 10000 |
| Chloridazon | 1.8 | 5.3 | 1750 | 0.14 | 0.42 | 350 | 1.0 | 3.0 | 2500 |
| Chlormequat | 7.0 | 21.0 | 1750 | 0.21 | 0.63 | 350 | 1.5 | 4.5 | 2500 |
| Chlorotoluron | 7.0 | 21.0 | 7000 | 0.28 | 0.84 | 1400 | 2.0 | 6.0 | 10000 |
| Chloroxuron | 7.0 | 21.0 | 7000 | 0.70 | 2.10 | 1400 | 5.0 | 15.0 | 10000 |
| Chlorpyrifos | 1.4 | 4.2 | 7000 | 0.28 | 0.84 | 350 | 2.0 | 6.0 | 2500 |
| Clethodim | 2.8 | 8.4 | 7000 | 0.14 | 0.42 | 1400 | 1.0 | 3.0 | 10000 |
| Clomazone | 1.8 | 5.3 | 7000 | 0.21 | 0.63 | 1400 | 1.5 | 4.5 | 10000 |
| Clothianidin | 5.6 | 16.8 | 1750 | 0.28 | 0.84 | 350 | 2.0 | 6.0 | 2500 |
| Coumaphos | 0.7 | 2.1 | 1750 | 0.14 | 0.42 | 350 | 1.0 | 3.0 | 2500 |
| Crotoxyphos | 0.7 | 2.1 | 7000 | 0.14 | 0.42 | 350 | 1.0 | 3.0 | 2500 |
| Cumyluron | 1.4 | 4.2 | 7000 | 0.08 | 0.25 | 1400 | 0.6 | 1.8 | 10000 |
| Cyanazine | 14.0 | 42.0 | 7000 | 0.42 | 1.26 | 1400 | 3.0 | 9.0 | 10000 |
| Cyantraniliprole | 14.0 | 42.0 | 1750 | 0.28 | 0.84 | 1400 | 3.0 | 9.0 | 2500 |
| Cyazofamid | 7.0 | 21.0 | 1750 | 0.28 | 0.84 | 350 | 2.0 | 6.0 | 2500 |
| Cycloate | 3.5 | 10.5 | 7000 | 0.70 | 2.10 | 1400 | 5.0 | 15.0 | 10000 |
| Cycluron | 2.1 | 6.3 | 7000 | 0.14 | 0.42 | 1400 | 1.0 | 3.0 | 10000 |
| Cyflufenamid | 1.4 | 4.2 | 7000 | 0.08 | 0.25 | 1400 | 0.6 | 1.8 | 10000 |
| Cyprodinil | 1.8 | 5.3 | 1750 | 0.28 | 0.84 | 350 | 2.0 | 6.0 | 2500 |
| Cyromazine | 7.0 | 21.0 | 7000 | 1.40 | 4.20 | 1400 | 10.0 | 30.0 | 10000 |
| Demeton-S-methylsulfone | 1.8 | 5.3 | 7000 | 0.14 | 0.42 | 1400 | 1.0 | 3.0 | 10000 |
| Desmedipham | 28.0 | 84.0 | 7000 | 0.42 | 1.26 | 1400 | 3.0 | 9.0 | 10000 |
| Desmetryn | 1.8 | 5.3 | 1750 | 0.14 | 0.42 | 350 | 1.0 | 3.0 | 2500 |
| Dicrotophos | 5.6 | 16.8 | 7000 | 0.14 | 0.42 | 1400 | 1.0 | 3.0 | 10000 |
| Diethofencarb | 3.5 | 10.5 | 7000 | 0.14 | 0.42 | 350 | 1.0 | 3.0 | 2500 |
| Difenacoum | 1.8 | 5.3 | 7000 | 0.14 | 0.42 | 1400 | 1.0 | 3.0 | 10000 |
| Difenoconazole | 2.1 | 6.3 | 1750 | 0.14 | 0.42 | 1400 | 1.0 | 3.0 | 10000 |
| Diflubenzuron | 2.8 | 8.4 | 1750 | 0.42 | 1.26 | 350 | 3.0 | 9.0 | 2500 |
| Dimefuron | 7.0 | 21.0 | 7000 | 0.28 | 0.84 | 350 | 2.0 | 6.0 | 2500 |
| Dimethametryn | 1.4 | 4.2 | 1750 | 0.28 | 0.84 | 350 | 2.0 | 6.0 | 2500 |
| Dimethenamid | 1.4 | 4.2 | 7000 | 0.28 | 0.84 | 1400 | 2.0 | 6.0 | 10000 |
| Dimethoate | 1.8 | 5.3 | 1750 | 0.28 | 0.84 | 350 | 2.0 | 6.0 | 2500 |
| Dimethomorph | 2.1 | 6.3 | 1750 | 0.28 | 0.84 | 350 | 2.0 | 6.0 | 2500 |
| Dimoxystrobin | 0.4 | 1.3 | 7000 | 0.08 | 0.25 | 1400 | 0.6 | 1.8 | 10000 |
| Diniconazole | 14.0 | 42.0 | 7000 | 1.40 | 4.20 | 1400 | 10.0 | 30.0 | 10000 |
| Dinotefuran | 7.0 | 21.0 | 7000 | 0.42 | 1.26 | 1400 | 3.0 | 9.0 | 10000 |
| Dithiopyr | 14.0 | 42.0 | 1750 | 1.40 | 4.20 | 350 | 10.0 | 30.0 | 2500 |
| Diuron | 3.5 | 10.5 | 7000 | 0.28 | 0.84 | 1400 | 2.0 | 6.0 | 10000 |
| DNOC | 14.0 | 42.0 | 7000 | 2.80 | 8.40 | 1400 | 20.0 | 60.0 | 10000 |
| Dodemorph | 1.8 | 5.3 | 1750 | 0.14 | 0.42 | 350 | 1.0 | 3.0 | 2500 |
| Epoxiconazole | 1.8 | 5.3 | 7000 | 0.28 | 0.84 | 1400 | 2.0 | 6.0 | 10000 |
| Esprocarb | 1.4 | 4.2 | 1750 | 0.14 | 0.42 | 350 | 1.0 | 3.0 | 2500 |
| Ethiofencarb | 70.0 | 210.0 | 1750 | 4.20 | 12.60 | 350 | 30.0 | 90.0 | 2500 |
| Ethiofencarb sulfone | 21.0 | 63.0 | 7000 | 0.70 | 2.10 | 1400 | 5.0 | 15.0 | 10000 |
| Ethiofencarb sulfoxide | 1.8 | 5.3 | 7000 | 0.21 | 0.63 | 1400 | 1.5 | 4.5 | 10000 |
| Ethiprole | 1.8 | 5.3 | 7000 | 0.28 | 0.84 | 350 | 2.0 | 6.0 | 2500 |
| Ethirimol | 1.8 | 5.3 | 1750 | 0.14 | 0.42 | 350 | 1.0 | 3.0 | 2500 |
| Ethofumesate | 28.0 | 84.0 | 1750 | 2.80 | 8.40 | 350 | 20.0 | 60.0 | 2500 |
| Ethoxyquin | 7.0 | 21.0 | 1750 | 1.40 | 4.20 | 350 | 10.0 | 30.0 | 2500 |
| Etofenprox | 0.4 | 1.3 | 1750 | 0.08 | 0.25 | 350 | 0.6 | 1.8 | 2500 |
| Etoxazole | 0.4 | 1.3 | 7000 | 0.08 | 0.25 | 1400 | 0.6 | 1.8 | 10000 |
| Etrimfos | 2.1 | 6.3 | 7000 | 0.28 | 0.84 | 350 | 2.0 | 6.0 | 2500 |
| Fenamidone | 3.5 | 10.5 | 1750 | 0.14 | 0.42 | 350 | 1.0 | 3.0 | 2500 |
| Fenamiphos | 0.7 | 2.1 | 7000 | 0.14 | 0.42 | 1400 | 1.0 | 3.0 | 10000 |
| Fenazaquin | 0.7 | 2.1 | 7000 | 0.14 | 0.42 | 1400 | 1.0 | 3.0 | 10000 |
| Fenbuconazole | 3.5 | 10.5 | 7000 | 0.28 | 0.84 | 1400 | 2.0 | 6.0 | 10000 |
| Fenhexamid | 7.0 | 21.0 | 7000 | 1.40 | 4.20 | 350 | 10.0 | 30.0 | 2500 |
| Fenobucarb | 3.5 | 10.5 | 7000 | 0.14 | 0.42 | 350 | 1.0 | 3.0 | 2500 |
| Fenoxycarb | 1.4 | 4.2 | 1750 | 0.14 | 0.42 | 350 | 1.0 | 3.0 | 2500 |
| Fenpyroximate | 1.4 | 4.2 | 1750 | 0.08 | 0.25 | 1400 | 0.6 | 1.8 | 10000 |
| Fensulfothion | 1.8 | 5.3 | 7000 | 0.08 | 0.25 | 1400 | 0.6 | 1.8 | 10000 |
| Fenthion | 7.0 | 21.0 | 7000 | 0.84 | 2.52 | 1400 | 6.0 | 18.0 | 10000 |
| Fenthion sulfoxide | 1.8 | 5.3 | 7000 | 0.14 | 0.42 | 1400 | 1.0 | 3.0 | 10000 |
| Fenuron | 7.0 | 21.0 | 1750 | 0.42 | 1.26 | 350 | 3.0 | 9.0 | 2500 |
| Fipronil | 4.2 | 12.6 | 438 | 0.84 | 2.52 | 88 | 6.0 | 18.0 | 625 |
| Fipronil sulfone | 0.7 | 2.1 | 1750 | 0.28 | 0.84 | 350 | 2.0 | 6.0 | 2500 |
| Florasulam | 70.0 | 210.0 | 1750 | 1.40 | 4.20 | 1400 | 10.0 | 30.0 | 10000 |
| Fluazifop | 14.0 | 42.0 | 1750 | 0.42 | 1.26 | 350 | 3.0 | 9.0 | 2500 |
| Fluazinam | 2.1 | 6.3 | 7000 | 0.42 | 1.26 | 1400 | 3.0 | 9.0 | 10000 |
| Flubendiamide | 70.0 | 210.0 | 7000 | 1.40 | 4.20 | 1400 | 10.0 | 30.0 | 10000 |
| Fludioxonil | 3.5 | 10.5 | 1750 | 1.40 | 4.20 | 350 | 10.0 | 30.0 | 2500 |
| Flufenacet | 0.7 | 2.1 | 1750 | 0.07 | 0.21 | 350 | 0.5 | 1.5 | 2500 |
| Flufenoxuron | 2.8 | 8.4 | 1750 | 0.14 | 0.42 | 1400 | 1.0 | 3.0 | 10000 |
| Flumetsulam | 28.0 | 84.0 | 1750 | 1.40 | 4.20 | 1400 | 10.0 | 30.0 | 10000 |
| Fluometuron | 7.0 | 21.0 | 1750 | 0.28 | 0.84 | 1400 | 2.0 | 6.0 | 10000 |
| Fluopicolide | 1.8 | 5.3 | 7000 | 0.14 | 0.42 | 1400 | 1.0 | 3.0 | 10000 |
| Fluopyram | 0.4 | 1.3 | 7000 | 0.08 | 0.25 | 1400 | 0.6 | 1.8 | 10000 |
| Fluorochloridone | 7.0 | 21.0 | 1750 | 1.40 | 4.20 | 350 | 10.0 | 30.0 | 2500 |
| Fluoxastrobin | 1.8 | 5.3 | 7000 | 0.14 | 0.42 | 350 | 1.0 | 3.0 | 2500 |
| Fluquinoconazole | 7.0 | 21.0 | 1750 | 0.28 | 0.84 | 1400 | 2.0 | 6.0 | 10000 |
| Flusilazole | 0.7 | 2.1 | 7000 | 0.14 | 0.42 | 1400 | 1.0 | 3.0 | 10000 |
| Flutriafol | 7.0 | 21.0 | 7000 | 0.56 | 1.68 | 1400 | 4.0 | 12.0 | 10000 |
| Fluxapyroxad | 0.7 | 2.1 | 7000 | 0.08 | 0.25 | 350 | 1.0 | 3.0 | 2500 |
| Forchlorfenuron | 2.1 | 6.3 | 7000 | 0.14 | 0.42 | 1400 | 1.0 | 3.0 | 10000 |
| Formetanate hydrochloride | 7.0 | 21.0 | 7000 | 0.42 | 1.26 | 1400 | 3.0 | 9.0 | 10000 |
| Formothion | 70.0 | 210.0 | 7000 | 1.40 | 4.20 | 350 | 10.0 | 30.0 | 2500 |
| Fosthiazate | 0.7 | 2.1 | 7000 | 0.14 | 0.42 | 1400 | 1.0 | 3.0 | 10000 |
| Fuberidazole | 3.5 | 10.5 | 1750 | 0.28 | 0.84 | 1400 | 2.0 | 6.0 | 10000 |
| Furathiocarb | 2.8 | 8.4 | 7000 | 0.28 | 0.84 | 1400 | 2.0 | 6.0 | 10000 |
| Griseofulvin | 1.4 | 4.2 | 7000 | 0.14 | 0.42 | 1400 | 1.0 | 3.0 | 10000 |
| Halofenozide | 3.5 | 10.5 | 7000 | 0.28 | 0.84 | 350 | 2.0 | 6.0 | 2500 |
| Haloxyfop | 10.5 | 31.5 | 1750 | 1.40 | 4.20 | 350 | 10.0 | 30.0 | 2500 |
| Haloxyfop-methyl | 0.6 | 1.7 | 1750 | 0.11 | 0.34 | 350 | 0.8 | 2.4 | 2500 |
| Heptenophos | 5.6 | 16.8 | 1750 | 0.42 | 1.26 | 350 | 3.0 | 9.0 | 2500 |
| Hexaconazole | 10.5 | 31.5 | 1750 | 1.40 | 4.20 | 1400 | 10.0 | 30.0 | 10000 |
| Hexaflumuron | 14.0 | 42.0 | 7000 | 2.80 | 8.40 | 1400 | 20.0 | 60.0 | 10000 |
| Hexazinone | 3.5 | 10.5 | 1750 | 0.14 | 0.42 | 350 | 1.0 | 3.0 | 2500 |
| Hexythiazox | 1.4 | 4.2 | 1750 | 0.28 | 0.84 | 1400 | 2.0 | 6.0 | 10000 |
| Imazalil | 3.5 | 10.5 | 1750 | 0.14 | 0.42 | 350 | 1.0 | 3.0 | 2500 |
| Imazaquin | 2.8 | 8.4 | 1750 | 0.28 | 0.84 | 350 | 2.0 | 6.0 | 2500 |
| Imazethapyr | 3.5 | 10.5 | 1750 | 0.14 | 0.42 | 350 | 1.0 | 3.0 | 2500 |
| Imibenconazole | 5.6 | 16.8 | 1750 | 0.56 | 1.68 | 1400 | 4.0 | 12.0 | 10000 |
| Imidacloprid | 3.5 | 10.5 | 1750 | 0.14 | 0.42 | 350 | 1.0 | 3.0 | 2500 |
| Indoxacarb | 14.0 | 42.0 | 1750 | 1.40 | 4.20 | 1400 | 10.0 | 30.0 | 10000 |
| Ioxynil | 7.0 | 21.0 | 7000 | 1.40 | 4.20 | 350 | 10.0 | 30.0 | 2500 |
| Iprovalicarb | 1.8 | 5.3 | 1750 | 1.40 | 4.20 | 1400 | 10.0 | 30.0 | 10000 |
| Isocarbophos | 7.0 | 21.0 | 7000 | 0.28 | 0.84 | 1400 | 2.0 | 6.0 | 10000 |
| Isoprocarb | 4.2 | 12.6 | 7000 | 0.28 | 0.84 | 1400 | 2.0 | 6.0 | 10000 |
| Isoprothiolane | 0.7 | 2.1 | 7000 | 0.08 | 0.25 | 1400 | 0.6 | 1.8 | 10000 |
| Isoproturon | 2.1 | 6.3 | 7000 | 0.28 | 0.84 | 1400 | 2.0 | 6.0 | 10000 |
| Isoxaben | 0.7 | 2.1 | 1750 | 0.08 | 0.25 | 350 | 0.6 | 1.8 | 2500 |
| Isoxadifen-ethyl | 1.8 | 5.3 | 1750 | 0.14 | 0.42 | 350 | 1.0 | 3.0 | 2500 |
| Kresoxim-methyl | 3.5 | 10.5 | 7000 | 0.28 | 0.84 | 1400 | 2.0 | 6.0 | 10000 |
| Lenacil | 7.0 | 21.0 | 7000 | 0.28 | 0.84 | 1400 | 2.0 | 6.0 | 10000 |
| Malaoxon | 0.8 | 2.5 | 1750 | 0.08 | 0.25 | 350 | 0.6 | 1.8 | 2500 |
| Mandipropamid | 7.0 | 21.0 | 7000 | 0.28 | 0.84 | 350 | 2.0 | 6.0 | 2500 |
| Mefenacet | 0.7 | 2.1 | 7000 | 0.07 | 0.21 | 1400 | 0.5 | 1.5 | 10000 |
| Mepiquat | 1.8 | 5.3 | 7000 | 0.14 | 0.42 | 350 | 1.0 | 3.0 | 2500 |
| Mepronil | 0.7 | 2.1 | 7000 | 0.08 | 0.25 | 350 | 0.6 | 1.8 | 2500 |
| Metalaxyl | 0.6 | 1.7 | 7000 | 0.08 | 0.25 | 1400 | 0.6 | 1.8 | 10000 |
| Metamitron | 21.0 | 63.0 | 1750 | 1.40 | 4.20 | 350 | 10.0 | 30.0 | 2500 |
| Metazachlor | 0.7 | 2.1 | 7000 | 0.08 | 0.25 | 350 | 0.6 | 1.8 | 2500 |
| Metconazole | 8.4 | 25.2 | 7000 | 0.84 | 1.68 | 350 | 6.0 | 12.0 | 2500 |
| Methabenzthiazuron | 0.8 | 2.5 | 7000 | 0.06 | 0.17 | 1400 | 0.4 | 1.2 | 10000 |
| Methamidophos | 7.0 | 21.0 | 1750 | 0.35 | 1.05 | 1400 | 2.5 | 7.5 | 10000 |
| Methiocarb | 3.5 | 10.5 | 7000 | 0.42 | 1.26 | 1400 | 3.0 | 9.0 | 10000 |
| Methiocarb sulfone | 7.0 | 21.0 | 7000 | 0.28 | 0.84 | 1400 | 2.0 | 6.0 | 10000 |
| Methiocarb sulfoxide | 1.8 | 5.3 | 1750 | 0.14 | 0.42 | 350 | 1.0 | 3.0 | 2500 |
| Methomyl | 3.5 | 10.5 | 7000 | 0.14 | 0.42 | 1400 | 1.0 | 3.0 | 10000 |
| Methoprotryne | 0.6 | 1.7 | 1750 | 0.07 | 0.21 | 350 | 0.5 | 1.5 | 2500 |
| Methoxyfenozide | 0.7 | 2.1 | 7000 | 0.14 | 0.42 | 1400 | 1.0 | 3.0 | 10000 |
| Metobromuron | 1.8 | 5.3 | 7000 | 0.14 | 0.42 | 1400 | 1.0 | 3.0 | 10000 |
| Metolachlor | 0.4 | 1.3 | 7000 | 0.08 | 0.25 | 1400 | 0.6 | 1.8 | 10000 |
| Metolcarb | 7.0 | 21.0 | 1750 | 0.28 | 0.84 | 350 | 2.0 | 6.0 | 2500 |
| Metosulam | 350.0 | 1050.0 | 1750 | 5.60 | 16.80 | 350 | 40.0 | 120.0 | 2500 |
| Metoxuron | 10.5 | 31.5 | 7000 | 0.28 | 0.84 | 1400 | 2.0 | 6.0 | 10000 |
| Metrafenone | 1.4 | 4.2 | 7000 | 0.28 | 0.84 | 350 | 2.0 | 6.0 | 2500 |
| Mevinphos I | 7.0 | 21.0 | 1750 | 1.40 | 4.20 | 350 | 10.0 | 30.0 | 2500 |
| Mevinphos II |  |  |  | 0.42 | 1.26 | 350 | 3.0 | 9.0 | 2500 |
| Mexacarbate | 0.4 | 1.1 | 7000 | 0.02 | 0.06 | 1400 | 0.2 | 0.5 | 10000 |
| Monocrotophos | 4.2 | 12.6 | 7000 | 0.28 | 0.84 | 1400 | 2.0 | 6.0 | 10000 |
| Monolinuron | 2.1 | 6.3 | 7000 | 0.14 | 0.42 | 1400 | 1.0 | 3.0 | 10000 |
| Myclobutanil | 3.5 | 10.5 | 1750 | 0.14 | 0.42 | 350 | 1.0 | 3.0 | 2500 |
| Napropamide | 0.7 | 2.1 | 7000 | 0.08 | 0.25 | 350 | 0.6 | 1.8 | 2500 |
| Neburon | 2.1 | 6.3 | 7000 | 0.28 | 0.84 | 1400 | 2.0 | 6.0 | 10000 |
| Nuarimol | 21.0 | 63.0 | 7000 | 1.40 | 4.20 | 350 | 10.0 | 30.0 | 2500 |
| Ofurace | 1.8 | 5.3 | 1750 | 0.14 | 0.42 | 350 | 1.0 | 3.0 | 2500 |
| Omethoate | 1.8 | 5.3 | 7000 | 0.14 | 0.42 | 1400 | 1.0 | 3.0 | 10000 |
| Oxadixyl | 3.5 | 10.5 | 1750 | 0.14 | 0.42 | 350 | 1.0 | 3.0 | 2500 |
| Oxamyl | 1.8 | 5.3 | 7000 | 0.14 | 0.42 | 1400 | 1.0 | 3.0 | 10000 |
| Paclobutrazole | 7.0 | 21.0 | 7000 | 0.28 | 0.84 | 1400 | 2.0 | 6.0 | 10000 |
| Penconazole | 1.8 | 5.3 | 7000 | 0.28 | 0.84 | 1400 | 2.0 | 6.0 | 10000 |
| Pencycuron | 1.4 | 4.2 | 7000 | 0.14 | 0.42 | 350 | 1.0 | 3.0 | 2500 |
| Penthiopyrad | 0.7 | 2.1 | 7000 | 0.14 | 0.42 | 1400 | 1.0 | 3.0 | 10000 |
| Phenmedipham | 70.0 | 210.0 | 7000 | 4.20 | 12.60 | 1400 | 30.0 | 90.0 | 10000 |
| Phenthoate | 3.5 | 10.5 | 1750 | 0.28 | 0.84 | 350 | 2.0 | 6.0 | 2500 |
| Phoxim | 0.7 | 2.1 | 1750 | 0.14 | 0.42 | 350 | 1.0 | 3.0 | 2500 |
| Picoxystrobin | 0.4 | 1.3 | 7000 | 0.08 | 0.25 | 350 | 0.6 | 1.8 | 2500 |
| Piperonyl butoxide | 0.4 | 1.3 | 7000 | 0.08 | 0.25 | 1400 | 0.6 | 1.8 | 10000 |
| Piperophos | 0.6 | 1.7 | 7000 | 0.08 | 0.25 | 1400 | 0.6 | 1.8 | 10000 |
| Pirimicarb | 7.0 | 21.0 | 1750 | 0.14 | 0.42 | 350 | 1.0 | 3.0 | 2500 |
| Pirimicarb desmethyl | 1.8 | 5.3 | 7000 | 0.08 | 0.25 | 1400 | 0.6 | 1.8 | 10000 |
| Pirimiphos-methyl | 0.7 | 2.1 | 7000 | 0.14 | 0.42 | 1400 | 1.0 | 3.0 | 10000 |
| Prochloraz | 3.5 | 10.5 | 1750 | 0.35 | 1.05 | 350 | 2.5 | 7.5 | 2500 |
| Profenophos | 0.7 | 2.1 | 1750 | 0.14 | 0.42 | 350 | 1.0 | 3.0 | 2500 |
| Promecarb | 1.4 | 4.2 | 1750 | 0.14 | 0.42 | 350 | 1.0 | 3.0 | 2500 |
| Prometon | 0.4 | 1.3 | 1750 | 0.08 | 0.25 | 350 | 0.6 | 1.8 | 2500 |
| Prometryn | 0.4 | 1.1 | 1750 | 0.07 | 0.21 | 350 | 0.5 | 1.5 | 2500 |
| Propamocarb | 1.8 | 5.3 | 7000 | 0.08 | 0.25 | 1400 | 0.6 | 1.8 | 10000 |
| Propazine | 0.7 | 2.1 | 7000 | 0.08 | 0.25 | 1400 | 0.6 | 1.8 | 10000 |
| Propetamphos | 14.0 | 42.0 | 7000 | 1.40 | 4.20 | 1400 | 10.0 | 30.0 | 10000 |
| Propiconazole | 3.5 | 10.5 | 1750 | 0.70 | 2.10 | 1400 | 5.0 | 15.0 | 2500 |
| Propoxur | 1.8 | 5.3 | 7000 | 0.28 | 0.84 | 350 | 2.0 | 6.0 | 2500 |
| Propyzamide | 2.1 | 6.3 | 7000 | 0.14 | 0.42 | 350 | 1.0 | 3.0 | 2500 |
| Prosulfocarb | 0.7 | 2.1 | 7000 | 0.08 | 0.25 | 350 | 0.6 | 1.8 | 2500 |
| Pymetrozine | 7.0 | 21.0 | 7000 | 0.42 | 1.26 | 350 | 3.0 | 9.0 | 2500 |
| Pyraclostrobin | 1.4 | 4.2 | 7000 | 0.14 | 0.42 | 1400 | 1.0 | 3.0 | 2500 |
| Pyrimethanil | 2.8 | 8.4 | 1750 | 0.28 | 0.84 | 350 | 2.0 | 6.0 | 2500 |
| Pyroxsulam | 21.0 | 63.0 | 1750 | 0.42 | 1.26 | 350 | 3.0 | 9.0 | 2500 |
| Quinoxyfen | 14.0 | 42.0 | 7000 | 2.80 | 8.40 | 1400 | 20.0 | 60.0 | 10000 |
| Quizalofop-ethyl | 2.1 | 6.3 | 1750 | 0.28 | 0.84 | 350 | 2.0 | 6.0 | 2500 |
| Resmethrin | 3.5 | 10.5 | 1750 | 0.35 | 1.05 | 1400 | 2.5 | 7.5 | 10000 |
| Rotenone | 5.6 | 16.8 | 7000 | 0.28 | 0.84 | 1400 | 2.0 | 6.0 | 10000 |
| Schradan | 0.8 | 2.5 | 1750 | 0.07 | 0.21 | 1400 | 0.5 | 1.5 | 10000 |
| Sethoxydim | 1.4 | 4.2 | 7000 | 0.14 | 0.42 | 350 | 1.0 | 3.0 | 2500 |
| Simeconazole | 2.8 | 8.4 | 7000 | 0.56 | 1.68 | 1400 | 4.0 | 12.0 | 10000 |
| Simetryn | 1.4 | 4.2 | 1750 | 0.14 | 0.42 | 350 | 1.0 | 3.0 | 2500 |
| Spinosyn A | 3.5 | 10.5 | 1750 | 0.35 | 1.05 | 1400 | 2.5 | 7.5 | 10000 |
| Spiromesifen | 21.0 | 63.0 | 1750 | 2.80 | 8.40 | 1400 | 20.0 | 60.0 | 10000 |
| Spirotetramat | 2.1 | 6.3 | 7000 | 0.14 | 0.42 | 1400 | 1.0 | 3.0 | 10000 |
| Spiroxamine | 0.7 | 2.1 | 1750 | 0.14 | 0.42 | 350 | 1.0 | 3.0 | 2500 |
| Sulfotep | 0.7 | 2.1 | 7000 | 0.14 | 0.42 | 1400 | 1.0 | 3.0 | 10000 |
| Sulprofos | 3.5 | 10.5 | 7000 | 0.28 | 0.84 | 1400 | 2.0 | 6.0 | 10000 |
| Tebuconazole | 7.0 | 21.0 | 1750 | 0.56 | 1.68 | 1400 | 4.0 | 12.0 | 10000 |
| Tebufenozide | 1.4 | 4.2 | 1750 | 0.28 | 0.84 | 350 | 2.0 | 6.0 | 2500 |
| Tebufenpyrad | 0.7 | 2.1 | 7000 | 0.14 | 0.42 | 350 | 1.0 | 3.0 | 2500 |
| Tebuthiuron | 0.8 | 2.5 | 1750 | 0.07 | 0.21 | 350 | 0.5 | 1.5 | 2500 |
| Teflubenzuron | 14.0 | 42.0 | 7000 | 2.80 | 8.40 | 1400 | 20.0 | 60.0 | 10000 |
| Tepraloxydim | 14.0 | 42.0 | 7000 | 2.80 | 8.40 | 1400 | 20.0 | 60.0 | 10000 |
| Terbumeton | 7.0 | 21.0 | 1750 | 0.28 | 0.84 | 350 | 2.0 | 6.0 | 2500 |
| Terbuthylazin | 3.5 | 10.5 | 1750 | 0.42 | 1.26 | 350 | 3.0 | 9.0 | 2500 |
| Terbutryn | 1.8 | 5.3 | 7000 | 0.28 | 0.84 | 350 | 2.0 | 6.0 | 2500 |
| Tetraconazole | 4.2 | 12.6 | 7000 | 0.28 | 0.84 | 1400 | 2.0 | 6.0 | 10000 |
| Tetramethrin | 3.5 | 10.5 | 1750 | 0.28 | 0.84 | 350 | 2.0 | 6.0 | 2500 |
| Thiabendazole | 1.4 | 4.2 | 7000 | 0.14 | 0.42 | 1400 | 1.0 | 3.0 | 10000 |
| Thiacloprid | 3.5 | 10.5 | 1750 | 0.14 | 0.42 | 350 | 1.0 | 3.0 | 2500 |
| Thiamethoxam | 1.8 | 5.3 | 1750 | 0.28 | 0.84 | 350 | 2.0 | 6.0 | 2500 |
| Thidiazuron | 28.0 | 84.0 | 1750 | 1.40 | 4.20 | 350 | 10.0 | 30.0 | 2500 |
| Thiobencarb | 1.4 | 4.2 | 1750 | 0.28 | 0.84 | 350 | 2.0 | 6.0 | 2500 |
| Thiophanate-methyl | 7.0 | 21.0 | 1750 | 0.14 | 0.42 | 350 | 1.0 | 3.0 | 2500 |
| Tralkoxydim | 2.8 | 8.4 | 1750 | 5.60 | 16.80 | 350 | 40.0 | 120.0 | 2500 |
| Triadimefon | 1.8 | 5.3 | 7000 | 0.08 | 0.25 | 1400 | 0.6 | 1.8 | 10000 |
| Triadimenol | 1.8 | 5.3 | 1750 | 0.35 | 1.05 | 350 | 2.5 | 7.5 | 2500 |
| Triazophos | 0.4 | 1.3 | 1750 | 0.08 | 0.25 | 350 | 0.6 | 1.8 | 2500 |
| Trichlorfon | 7.0 | 21.0 | 1750 | 0.42 | 1.26 | 1400 | 3.0 | 9.0 | 10000 |
| Tricyclazole | 0.7 | 2.1 | 1750 | 0.03 | 0.08 | 350 | 0.2 | 0.6 | 2500 |
| Trietazine | 1.4 | 4.2 | 7000 | 0.28 | 0.84 | 1400 | 2.0 | 6.0 | 10000 |
| Trifloxystrobin | 0.7 | 2.1 | 1750 | 0.08 | 0.25 | 350 | 0.6 | 1.8 | 2500 |
| Triflumizole | 7.0 | 21.0 | 7000 | 0.08 | 0.25 | 350 | 0.6 | 1.8 | 2500 |
| Vamidothion | 1.8 | 5.3 | 1750 | 0.14 | 0.42 | 350 | 1.0 | 3.0 | 2500 |
| Zoxamide | 1.8 | 5.3 | 7000 | 0.28 | 0.84 | 1400 | 2.0 | 6.0 | 10000 |

**Table S4.** Toxicity data used to calculate RQ values. Values taken from PPDB except where noted. Italicized AIs indicate data deficiencies.

|  |  | ***Apis mellifera* toxicity** | | ***Bombus terrestris* toxicity** | |  |
| --- | --- | --- | --- | --- | --- | --- |
| **Active Ingredient** | **Type** | **contact LD_50_ (μg bee⁻¹)** | **oral LD_50_ (μg bee⁻¹)** | **contact LD_50_ (μg bee⁻¹)** | **oral LD_50_ (μg bee⁻¹)** | **Source other than PPDB** |
| acetamiprid | insecticide | 8.09 | 14.53 | 100 | 22.2 |  |
| amitraz | miticide | 50 |  |  |  |  |
| ancymidol | herbicide | 100 |  |  |  |  |
| atrazine | herbicide | 100 | 100 |  |  |  |
| avermectin.b1a | insecticide | 0.002 | 0.009 |  |  |  |
| *azaconazole* | fungicide | *no data* |  |  |  |  |
| azinphos.methyl | insecticide | 0.42 |  |  |  |  |
| azoxystrobin | fungicide | 200 | 25 |  |  |  |
| benoxacor | herbicide |  | 100 |  |  |  |
| *benzoximate* | miticide | *no data* |  |  |  |  |
| boscalid | fungicide | 200 | 166 |  |  |  |
| *bromacil* | herbicide | *no data* |  |  |  |  |
| bromuconazole | fungicide | 500 | 100 |  |  |  |
| buprofezin | insecticide | 200 | 163.5 |  | 69 |  |
| carbaryl | insecticide | 0.14 | 0.21 | 41.2 | 3.84 |  |
| carbendazim | fungicide | 50 | 756 |  |  |  |
| carbofuran | insecticide | 0.036 | 0.05 |  |  |  |
| chlorantraniliprole | insecticide | 100 | 104.1 | 100 | 100 |  |
| chlorfenvinphos | insecticide | 4.1 | 0.55 |  |  | Sanchez-Bayo and Goka 2014^53^ |
| chlorpyrifos | insecticide | 0.059 | 0.25 | 1.58 | 0.23 |  |
| clomazone | herbicide | 89.5 | 76.33 | 968 | 968 |  |
| clothianidin | insecticide | 0.044 | 0.004 | 0.02 |  |  |
| coumaphos | miticide | 20 | 4.6 |  |  | Sanchez-Bayo and Goka 2014^53^ |
| cyantraniliprole | insecticide | 0.0934 |  |  |  |  |
| cyprodinil | fungicide | 784 | 112.5 |  |  |  |
| *difenacoum* | rodenticide | *no data* |  |  |  |  |
| difenoconazole | fungicide | 100 | 177 |  |  |  |
| diflubenzuron | insecticide | 74.2 | 9.1 | 0.1 | 1.46 |  |
| dimethenamid | herbicide | 94 |  |  |  |  |
| dithiopyr | herbicide | 53 |  |  |  |  |
| diuron | herbicide | 101.7 | 86.75 | 500 | 445.8 |  |
| ethofumesate | herbicide | 50 | 50 |  |  |  |
| *ethoxyquin* | antioxidant | *no data* |  |  |  |  |
| fenbuconazole | fungicide | 5.5 | 5.2 |  |  |  |
| fenpyroximate | miticide | 15.8 | 118.5 |  |  |  |
| fludioxonil | fungicide | 100 | 100 |  |  |  |
| fluopyram | fungicide | 100 | 102.3 |  |  |  |
| fluoxastrobin | fungicide | 200 | 843 | 100 | 209 |  |
| flutriafol | fungicide | 50 | 2 |  |  |  |
| fluxapyroxad | fungicide | 100 | 110.9 |  |  |  |
| hexazinone | herbicide | 100 |  |  |  |  |
| imidacloprid | insecticide | 0.081 | 0.0037 | 0.218 | 0.038 |  |
| indoxacarb | insecticide | 0.08 | 0.232 | 0.25 | 0.07 |  |
| *isocarbophos* | insecticide | *no data* |  |  |  |  |
| *isoprothiolane* | fungicide | *no data* |  |  |  |  |
| isoxaben | herbicide | 100 | 100 |  |  |  |
| *isoxadifen.ethyl* | herbicide | *no data* |  |  |  |  |
| *malaoxon* | insecticide | *no data* |  |  |  |  |
| metalaxyl | fungicide | 200 | 269 |  |  |  |
| metconazole | fungicide | 100 | 85 | 100 | 111.1 |  |
| methomyl | insecticide | 0.16 | 0.28 |  | 3.3 |  |
| *methoprotryne* | herbicide | *no data* |  |  |  |  |
| methoxyfenozide | insecticide | 100 | 2000 |  |  |  |
| metolachlor | herbicide | 110 | 110 |  |  |  |
| myclobutanil | herbicide | 33.9 | 33.9 |  |  |  |
| napropamide | herbicide | 100 | 100 |  |  |  |
| penthiopyrad | fungicide | 500 | 500 |  |  |  |
| picoxystrobin | fungicide | 200 | 200 |  |  |  |
| piperonyl.butoxide | synergist | 294 |  |  |  |  |
| prometon | herbicide | 36 |  |  |  |  |
| prometryn | herbicide | 99 |  |  |  |  |
| propamocarb | fungicide | 100 | 84 |  |  |  |
| propazine | herbicide | 16 |  |  |  |  |
| propiconazole | fungicide | 100 | 100 |  |  |  |
| pyraclostrobin | fungicide | 100 | 110 | 100 | 97.2 |  |
| pyrimethanil | fungicide | 100 | 100 | 400 | 579.7 |  |
| sethoxydim | herbicide | 10 |  |  |  |  |
| *spinosyn.a* | insecticide | *no data* |  |  |  |  |
| spiromesifen | insecticide | 200 | 792.4 |  |  |  |
| spirotetramat | insecticide | 100 | 107.3 |  |  |  |
| tebuconazole | fungicide | 200 | 83.05 |  |  |  |
| tebufenozide | insecticide | 234 | 100 |  |  |  |
| tebufenpyrad | insecticide | 6.7 | 60.3 |  |  |  |
| tebuthiuron | herbicide | 30 |  |  |  |  |
| *tetramethrin* | insecticide | *no data* |  |  |  |  |
| thiabendazole | fungicide | 34 | 4 |  |  |  |
| thiacloprid | insecticide | 38.82 | 17.32 | 100 |  |  |
| thiamethoxam | insecticide | 0.024 | 0.005 | 0.028 | 0.005 |  |
| thiobencarb | herbicide | 100 | 100 |  |  |  |
| thiophanate.methyl | fungicide | 100 | 114.7 |  |  |  |
| triadimenol | fungicide | 200 | 224.8 |  |  |  |
| trifloxystrobin | fungicide | 100 | 110 |  |  |  |

**Table S5.** Active ingredients detected in samples other than pollen from 2019, ordered by percent of samples with detections. Shaded cells indicate active ingredients that were found in over 50% of all samples. Pollen data published in Graham et al., 2021^21^.

|  |  |  |  |  |  | **Honey bee wax** | | | | **Blueberry flowers** | | | | **Whole honey bees** | | | | **Whole bumble bees** | | | |
| --- | --- | --- | --- | --- | --- | --- | --- | --- | --- | --- | --- | --- | --- | --- | --- | --- | --- | --- | --- | --- | --- |
| **Active ingredient** | **Type** | **Registered on blueb.** | **Applied to sampled fields** | **Applied to blueberry during bloom** | **Applied to honey bees hives** | **Mean (ppb)** | **StDev** | **% of samp. with detects** | **Max (ppb)** | **Mean (ppb)** | **StDev** | **% of samp. with detects** | **Max (ppb)** | **Mean (ppb)** | **StDev** | **% of samp. with detects** | **Max (ppb)** | **Mean (ppb)** | **StDev** | **% of samp. with detects** | **Max (ppb)** |
| fenbuconazole | F | x | x | x |  | 11.03 | 10.87 | 83.19 | 50.06 | 79.92 | 94.14 | 75.00 | 444.72 | 58.04 | 97.70 | 72.41 | 426.50 | 36.70 | 35.18 | 61.54 | 116.51 |
| methoxyfenozide | I | x | x | x |  | 44.04 | 194.51 | 99.12 | 1908.19 | 116.65 | 342.39 | 70.00 | 1190.39 | 1513.48 | 2618.39 | 34.48 | 7652.00 | 394.81 | 471.60 | 38.46 | 1163.68 |
| fluopyram | F | x | x | x |  | 25.94 | 58.47 | 80.53 | 291.04 | 146.81 | 329.01 | 55.00 | 1400.00 | 174.82 | 264.07 | 62.07 | 915.19 | 47.05 | 61.94 | 30.77 | 130.78 |
| boscalid | F | x | x | x |  | 24.37 | 69.85 | 75.22 | 607.39 | 166.09 | 158.33 | 42.50 | 373.80 | 1390.07 | 2637.19 | 62.07 | 8794.90 | 204.86 | 416.14 | 46.15 | 1053.57 |
| pyraclostrobin | F | x | x | x |  | 26.35 | 89.83 | 86.73 | 855.18 | 359.93 | 440.43 | 32.50 | 1400.00 | 345.11 | 695.10 | 58.62 | 2208.12 | 71.69 | 139.69 | 38.46 | 321.50 |
| azoxystrobin | F | x | x | x |  | 15.78 | 23.26 | 97.35 | 152.62 | 0.59 | 0.82 | 67.50 | 3.22 | 44.47 | 77.28 | 31.03 | 246.04 | 6.87 | 8.07 | 15.38 | 12.58 |
| propiconazole | F | x | x | x |  | 19.88 | 31.35 | 65.49 | 187.93 | 53.24 | 147.61 | 32.50 | 542.21 | 27.29 | 52.10 | 31.03 | 156.91 | 98.30 | 139.14 | 23.08 | 257.81 |
| metolachlor | H | x | x | x |  | 1.26 | 1.61 | 16.81 | 7.32 | 0.39 | 0.72 | 75.00 | 3.62 | 0.33 | 0.10 | 13.79 | 0.48 | 0.88 | 1.09 | 23.08 | 2.13 |
| carbendazim | F |  |  |  |  | 1562.20 | 4137.83 | 19.47 | 18181.82 |  |  | 0.00 |  | 440.83 | 251.90 | 55.17 | 1008.23 | 485.65 | 225.38 | 53.85 | 701.51 |
| diuron | H | x | x | x |  | 4.06 | 5.27 | 32.74 | 27.17 | 1.49 | 1.85 | 47.50 | 6.56 | 61.77 | 129.49 | 20.69 | 325.87 | 1.97 | 0.46 | 15.38 | 2.30 |
| atrazine | H |  |  |  |  | 3.01 | 4.17 | 19.47 | 17.37 | 0.41 | 0.36 | 92.50 | 1.38 |  |  | 0.00 |  |  |  | 0.00 |  |
| pyrimethanil | F | x | x | x |  | 46.78 | 80.97 | 47.79 | 312.94 | 350.00 | 0.00 | 15.00 | 350.00 | 246.22 | 139.17 | 24.14 | 471.81 | 106.88 | 94.42 | 15.38 | 173.65 |
| coumaphos | M |  |  |  | x | 36.17 | 91.04 | 100.00 | 728.24 |  |  | 0.00 |  |  |  | 0.00 |  |  |  | 0.00 |  |
| cyprodinil | F | x |  |  |  | 10.85 | 17.73 | 73.45 | 138.76 | 1.43 | 2.03 | 22.50 | 6.55 |  |  | 0.00 |  |  |  | 0.00 |  |
| piperonyl butoxide | S |  |  |  |  | 59.48 | 88.28 | 83.19 | 511.84 |  |  | 0.00 |  |  |  | 0.00 |  |  |  | 0.00 |  |
| methoprotryne | H |  |  |  |  | 1.32 | 1.22 | 70.80 | 5.21 |  |  | 0.00 |  |  |  | 0.00 |  |  |  | 0.00 |  |
| metconazole | F | x | x | x |  | 21.83 | 37.69 | 26.55 | 202.87 | 4.58 | 2.39 | 12.50 | 7.02 | 7.56 | 2.73 | 10.34 | 9.85 | 62.36 |  | 7.69 | 62.36 |
| chlorpyrifos | I |  |  |  |  | 3.80 | 6.44 | **35.40** | 37.54 | 0.28 | 0.00 | **15.00** | 0.28 | 1.60 |  | **3.45** | 1.60 |  |  | **0.00** |  |
| diflubenzuron | I |  |  |  |  | 10.74 | 15.93 | 39.82 | 82.19 |  |  |  |  |  |  | 0.00 |  |  |  | 0.00 |  |
| difenoconazole | F |  |  |  |  | 6.46 | 11.02 | 52.21 | 69.93 |  |  | 0.00 |  |  |  | 0.00 |  |  |  | 0.00 |  |
| malaoxon* | I | x | x |  |  |  |  |  |  | 0.62 | 0.77 | 37.50 | 2.51 |  |  | 0.00 |  |  |  | 0.00 |  |
| fludioxonil | F | x |  |  |  | 27.50 | 27.76 | 5.31 | 70.95 |  |  | 0.00 |  | 28.05 | 0.56 | 6.90 | 28.44 | 15.57 | 10.34 | 30.77 | 26.94 |
| trifloxystrobin | F |  |  |  |  | 1.36 | 1.49 | 25.66 | 5.95 | 1.22 | 1.19 | 10.00 | 2.30 |  |  | 0.00 |  |  |  | 0.00 |  |
| imidacloprid | I | x | x |  |  | 1.83 | 1.32 | 6.19 | 3.69 | 0.14 | 0.00 | 25.00 | 0.14 |  |  | 0.00 |  |  |  | 0.00 |  |
| propamocarb | F |  |  |  |  | 1.47 | 1.82 | 27.43 | 6.20 |  |  | 0.00 |  |  |  | 0.00 |  |  |  | 0.00 |  |
| fenpyroximate | A |  |  |  |  | 3.16 | 3.42 | 26.55 | 10.67 |  |  | 0.00 |  |  |  | 0.00 |  |  |  | 0.00 |  |
| tebuconazole | F |  |  |  |  | 15.30 | 26.02 | 26.55 | 97.00 |  |  | 0.00 |  |  |  | 0.00 |  |  |  | 0.00 |  |
| tebufenozide | I |  |  |  |  | 5.62 | 5.81 | 14.16 | 18.50 | 2.29 | 0.50 | 7.50 | 2.76 | 1.60 |  | 3.45 | 1.60 |  |  | 0.00 |  |
| penthiopyrad | F |  |  |  |  | 4.50 | 10.08 | 23.01 | 52.12 |  |  | 0.00 |  |  |  | 0.00 |  |  |  | 0.00 |  |
| dimethenamid | H |  |  |  |  |  |  |  |  | 0.28 | 0.00 | 7.50 | 0.28 |  |  | 0.00 |  | 0.92 |  | 7.69 | 0.92 |
| buprofezin | I |  |  |  |  | 0.98 | 0.79 | 19.47 | 2.89 |  |  | 0.00 |  |  |  | 0.00 |  |  |  | 0.00 |  |
| chlorantraniliprole | I |  |  |  |  | 5.49 | 5.55 | 17.70 | 17.68 |  |  | 0.00 |  |  |  | 0.00 |  |  |  | 0.00 |  |
| bromacil | H |  |  |  |  | 3.16 | 2.68 | 16.81 | 13.17 |  |  | 0.00 |  |  |  | 0.00 |  |  |  | 0.00 |  |
| triadimenol | F |  |  |  |  | 10.74 | 7.17 | 7.96 | 22.51 | 1.79 | 0.51 | 7.50 | 2.29 |  |  | 0.00 |  |  |  | 0.00 |  |
| cyantraniliprole | I | x | x |  |  |  |  |  |  | 1.07 | 0.92 | 10.00 | 2.00 |  |  | 0.00 |  |  |  | 0.00 |  |
| fluxapyroxad | F |  |  |  |  | 2.60 | 2.67 | 9.73 | 8.43 |  |  | 0.00 |  |  |  | 0.00 |  |  |  | 0.00 |  |
| carbaryl | I | x |  |  |  | 1.27 | 0.09 | 2.65 | 1.38 | 0.28 |  | 2.50 | 0.28 | 160.55 |  | 3.45 | 160.55 |  |  | 0.00 |  |
| methomyl | I | x | x | x |  |  |  |  |  | 0.14 | 0.00 | 5.00 | 0.14 |  |  | 0.00 |  |  |  | 0.00 |  |
| metalaxyl | F |  |  |  |  | 26.38 | 64.58 | 6.19 | 172.74 |  |  | 0.00 |  |  |  | 0.00 |  |  |  | 0.00 |  |
| myclobutanil | F |  |  |  |  | 0.69 | 0.33 | 3.54 | 1.03 | 0.14 |  | 2.50 | 0.14 |  |  | 0.00 |  |  |  | 0.00 |  |
| acetamiprid | I | x | x |  |  | 0.20 |  | 0.88 | 0.20 | 0.08 | 0.00 | 5.00 | 0.08 |  |  | 0.00 |  |  |  | 0.00 |  |
| amitraz | M |  |  |  | x | 3.81 | 4.06 | 3.54 | 9.84 |  |  | 0.00 |  |  |  | 0.00 |  |  |  | 0.00 |  |
| spirotetramat | I |  |  |  |  | 0.81 | 0.19 | 3.54 | 1.06 |  |  | 0.00 |  |  |  | 0.00 |  |  |  | 0.00 |  |
| cumyluron | H |  |  |  |  | 0.35 | 0.01 | 1.77 | 0.36 |  |  | 0.00 |  |  |  | 0.00 |  |  |  | 0.00 |  |
| fluopicolide | F |  |  |  |  | 0.89 | 0.11 | 1.77 | 0.96 |  |  | 0.00 |  |  |  | 0.00 |  |  |  | 0.00 |  |
| thiophanate methyl | F |  |  |  |  | 2.06 | 0.92 | 1.77 | 2.71 |  |  | 0.00 |  |  |  | 0.00 |  |  |  | 0.00 |  |
| bifenazate | A | on non-bearing plants |  |  |  | 0.44 |  | 0.88 | 0.44 |  |  | 0.00 |  |  |  | 0.00 |  |  |  | 0.00 |  |
| dimethomorph | F |  |  |  |  | 1.61 |  | 0.88 | 1.61 |  |  | 0.00 |  |  |  | 0.00 |  |  |  | 0.00 |  |
| fenhexamid | F | x |  | x |  | 3.32 |  | 0.88 | 3.32 |  |  | 0.00 |  |  |  | 0.00 |  |  |  | 0.00 |  |
| fluazifop | F | x |  | x |  | 2.42 |  | 0.88 | 2.42 |  |  | 0.00 |  |  |  | 0.00 |  |  |  | 0.00 |  |
| fluazinam | F | x |  | x |  | 2.65 |  | 0.88 | 2.65 |  |  | 0.00 |  |  |  | 0.00 |  |  |  | 0.00 |  |
| fluoxastrobin | F |  |  |  |  | 0.76 |  | 0.88 | 0.76 |  |  | 0.00 |  |  |  | 0.00 |  |  |  | 0.00 |  |
| indoxacarb | I |  |  |  |  | 8.33 |  | 0.88 | 8.33 |  |  | 0.00 |  |  |  | 0.00 |  |  |  | 0.00 |  |
| metrafenone | F |  |  |  |  | 2.92 |  | 0.88 | 2.92 |  |  | 0.00 |  |  |  | 0.00 |  |  |  | 0.00 |  |
| neburon | H |  |  |  |  | 6.58 |  | 0.88 | 6.58 |  |  | 0.00 |  |  |  | 0.00 |  |  |  | 0.00 |  |
| propoxur | I |  |  |  |  | 4.26 |  | 0.88 | 4.26 |  |  | 0.00 |  |  |  | 0.00 |  |  |  | 0.00 |  |
| thiamethoxam | I |  |  |  |  | 1.23 |  | 0.88 | 1.23 |  |  | 0.00 |  |  |  | 0.00 |  |  |  | 0.00 |  |

*Breakdown product of malathion

Pesticide types: I=insecticide, F=fungicide, H=herbicide, M=miticide (in-hive treatment for Varroa), A=acaracide, S=synergist

**Table S6.** Statistical results not reported in the text, for the effect of farm management on pesticide residues for various bee and flower related matrices.

|  | Sample type | Exposure | R^2^m or R_adj_ | R2c | *X*^2^ or F statistic | df | p-value |  |
| --- | --- | --- | --- | --- | --- | --- | --- | --- |
| Farm management effect on number of active ingredients | | | | | | | | |
|  | flowers |  | 0.32 | 0.32 | 15.33 | 1 | **< 0.001** |  |
|  | whole honey bees |  | 0.21 | 0.21 | 5.13 | 1 | **0.02** |  |
|  | whole bumble bees |  | 0.23 | 0.42 | 4710.80 | 1 | **< 0.001** |  |
|  | honey bee pollen 2018 |  | 0.12 | 0.21 | 8.31 | 2 | **0.02** |  |
|  | honey bee pollen 2019 |  | < 0.01 | 0.16 | 0.01 | 1 | 0.90 |  |
|  | bumble bee pollen 2019 |  | 0.02 |  | 0.30 | 1 | 0.58 |  |
|  | honey bee wax |  | 0.01 | 0.17 | 0.50 | 1 | 0.48 |  |
| Farm management effect on concentration of pesticides | | | | | | | | |
|  | flowers |  | 0.80 | 0.80 | 157.66 | 1 | **< 0.001** |  |
|  | whole honey bees |  | 0.43 | 0.43 | 20.73 | 1 | **< 0.001** |  |
|  | whole bumble bees |  | 0.01 | 0.01 | 0.12 | 1 | 0.73 |  |
|  | honey bee pollen 2018 |  | 0.32 | 0.74 | 10.02 | 2 | **0.007** |  |
|  | honey bee pollen 2019 |  | 0.17 | 0.46 | 6.04 | 1 | **0.01** |  |
|  | bumble bee pollen 2019 |  | 0.62 |  | F_1,14_ = 11.60 | | **< 0.001** |  |
|  | wax |  | 0.03 | 0.07 | 2.88 | 1 | 0.09 |  |
| Farm management effect on sample RQ | | | | | | | | |
|  | flowers (honey bee tox.) | contact | 0.45 | 0.75 | 17.2 | 1 | < 0.001 |  |
|  |  | oral | 0.67 | 0.84 | 43.21 | 1 | < 0.001 |  |
|  | flowers (bumble bee tox.) | contact | 0.65 | 0.68 | 65.4 | 1 | < 0.001 |  |
|  |  | oral | 0.73 | 0.84 | 65.29 | 1 | < 0.001 |  |
|  | whole honey bees | contact | 0.32 | 0.81 | 10.7 | 1 | 0.001 |  |
|  |  | oral | 0.18 | 0.77 | 5.13 | 1 | 0.02 |  |
|  | whole bumble bees | contact | 0.10 | 0.98 | 1.2 | 1 | 0.27 |  |
|  |  | oral | < 0.01 | 0.62 | 0.04 | 1 | 0.85 |  |
|  | honey bee pollen 2018 | contact | 0.03 | 0.65 | 0.51 | 2 | 0.78 |  |
|  |  | oral | 0.01 | 0.32 | 0.27 | 2 | 0.87 |  |
|  | honey bee pollen 2019 | contact | 0.01 | 0.52 | 0.27 | 1 | 0.60 |  |
|  |  | oral | 0.01 | 0.26 | 0.37 | 1 | 0.54 |  |
|  | bumble bee pollen 2019 | contact | 0.16 |  | F_1,14_ = 2.51 | | 0.14 |  |
|  |  | oral | 0.03 |  | F_1,14_ = 0.44 | | 0.52 |  |
|  | honey bee wax | contact | < 0.01 | 0.03 | < 0.01 | 1 | 0.98 |  |
|  |  | oral | < 0.01 | 0.02 | 0.05 | 1 | 0.82 |  |

**Table S7.** Correlation (Pearson’s r) of agricultural landcover types and pollen risk quotient at three scales. RQ was calculated with both contact LD_50_s and oral LD_50_s and correlations are displayed separately. Significant correlations are bolded with level of significant indicated with asterisks.

|  | **Landcover** | **Scale (radius)** | **RQ with Contact LD_50_ (Pearson’s r)** | **RQ with Contact LD_50_ (Pearson’s r), w/o Farm 13** | **RQ with Oral LD_50_ (Pearson’s r)** | **RQ with Oral LD_50_ (Pearson’s r),**  **w/o Farm 13** |
| --- | --- | --- | --- | --- | --- | --- |
| Honey bees 2018 | Blueberry | 500m | -0.23 | 0.16 | -0.25 | 0.17 |
|  |  | 1000m | -0.17 | 0.17 | -0.19 | 0.14 |
|  |  | 2000m | -0.10 | 0.49 | -0.17 | 0.39 |
|  | Apple | 500m | 0.04 | 0.19 | -0.02 | 0.03 |
|  |  | 1000m | **0.81**** | 0.32 | **0.78*** | 0.08 |
|  |  | 2000m | **0.89***** | 0.58 | **0.85**** | 0.35 |
|  | Cherry | 500m | 0.14 | 0.52 | 0.00 | 0.16 |
|  |  | 1000m | 0.60 | 0.58 | 0.45 | 0.07 |
|  |  | 2000m | **0.75*** | 0.50 | 0.65 | 0.08 |
|  | Other crops | 500m | 0.07 | 0.70 | 0.00 | 0.64 |
|  |  | 1000m | 0.39 | 0.73 | 0.32 | 0.63 |
|  |  | 2000m | 0.36 | 0.52 | 0.34 | 0.59 |
| Honey bees 2019 | Blueberry | 500m | -0.25 | -0.10 | -0.32 | -0.07 |
|  |  | 1000m | -0.27 | -0.16 | -0.24 | -0.01 |
|  |  | 2000m | -0.14 | 0.01 | 0.05 | 0.52 |
|  | Apple | 500m | 0.04 | -0.02 | 0.01 | -0.17 |
|  |  | 1000m | 0.39 | -0.13 | 0.72 | -0.17 |
|  |  | 2000m | 0.32 | -0.12 | **0.75*** | 0.26 |
|  | Cherry | 500m | **0.83**** | **0.91***** | 0.18 | 0.26 |
|  |  | 1000m | 0.58 | 0.42 | 0.51 | 0.01 |
|  |  | 2000m | 0.48 | 0.25 | 0.60 | 0.02 |
|  | Other crops | 500m | 0.15 | 0.26 | 0.19 | 0.56 |
|  |  | 1000m | 0.43 | 0.43 | 0.48 | 0.65 |
|  |  | 2000m | 0.35 | 0.32 | 0.40 | 0.47 |
| Bumble bees 2019 | Blueberry | 500m | 0.23 | 0.49 | -0.10 | 0.23 |
|  |  | 1000m | 0.15 | 0.37 | -0.08 | 0.22 |
|  |  | 2000m | 0.01 | 0.22 | 0.03 | 0.44 |
|  | Apple | 500m | 0.59 | 0.42 | **0.77*** | 0.28 |
|  |  | 1000m | 0.50 | 0.09 | 0.67 | 0.01 |
|  |  | 2000m | 0.47 | 0.08 | **0.73*** | 0.29 |
|  | Cherry | 500m | 0.45 | 0.19 | 0.50 | 0.04 |
|  |  | 1000m | 0.18 | -0.14 | 0.31 | -0.16 |
|  |  | 2000m | 0.37 | -0.06 | 0.60 | 0.06 |
|  | Other crops | 500m | -0.21 | -0.13 | -0.02 | 0.19 |
|  |  | 1000m | 0.03 | 0.00 | 0.26 | 0.32 |
|  |  | 2000m | 0.02 | -0.05 | 0.23 | 0.21 |

p < .0001 ‘****’; p < .001 ‘***’, p < .01 ‘**’, p < .05 ‘*’

P-values corrected for multiple inference using the Holm’s method


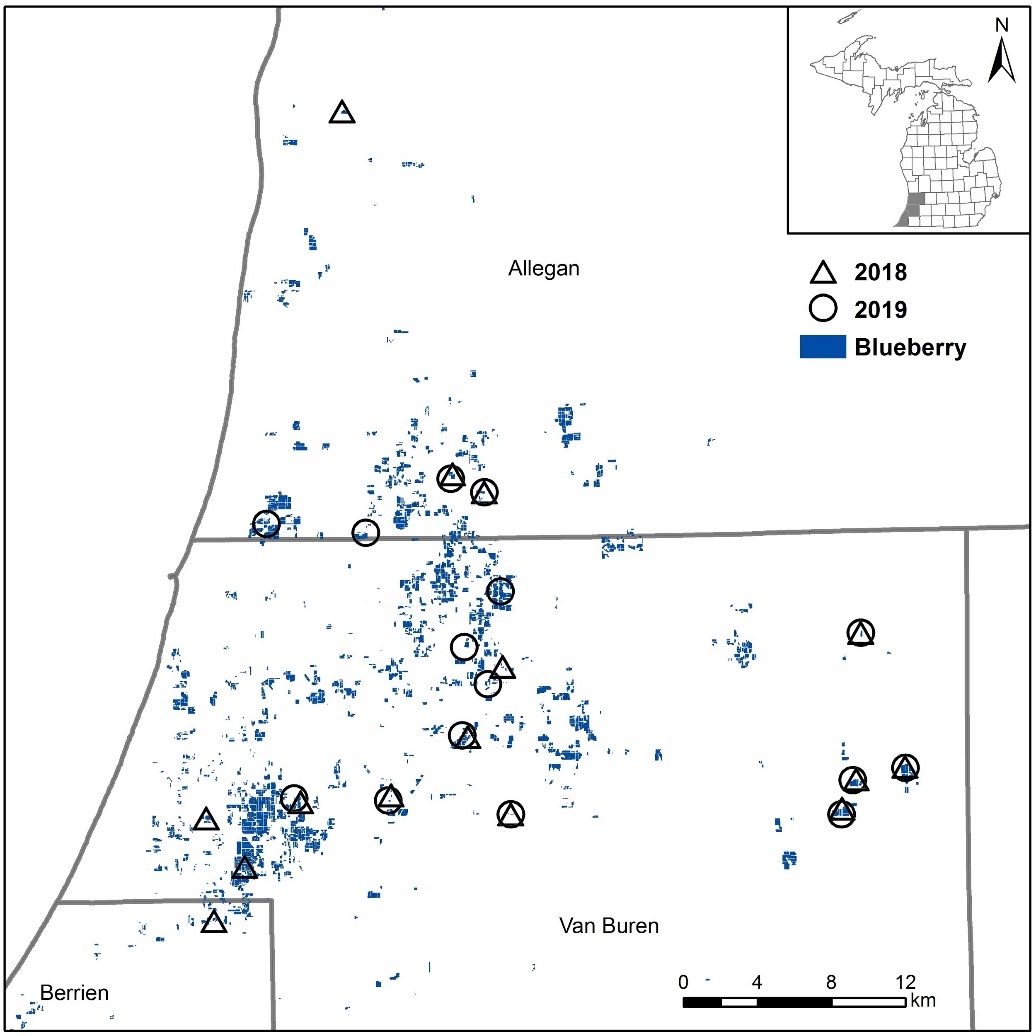


**Figure S1.** Locations of blueberry fields where samples were collected for pesticide residues in 2018 and 2019. Blue patches indicate the location of blueberry fields, determined by analysis of aerial imagery. Map created using ArcGIS® software by Esri. ArcGIS® and ArcMap™ are the intellectual property of Esri and are used under license. Copyright © Esri. All rights reserved. For more information about Esri® software, please visit www.esri.com. Basemap data sources: ArcUSA, U.S. Census, USDA, and Esri.


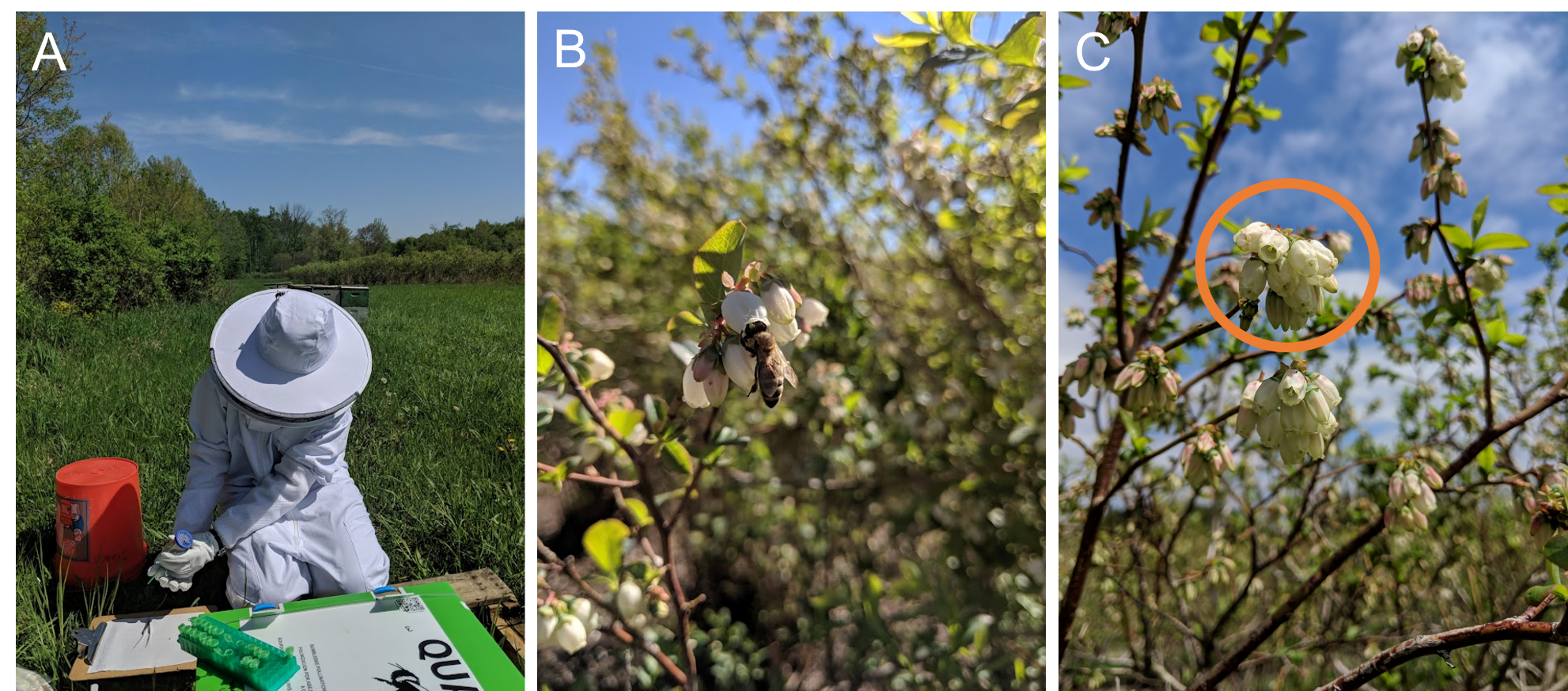


**Figure S2.** Pictural descriptions of some of our sampling methods. (A) We hand collected pollen from the corbiculae of returning bumble bee foragers (managed colonies of *Bombus impatiens*) by blocking the entrance to the hive and then capturing them in honey bee queen marking tubes, and removing pollen from their corbiculae using tweezers. (B) We collected honey bee foragers at blueberry flowers by placing a cyanide kill jar around a nectaring honey bee, as pictured. (C) We collected 10 blueberry flower clusters (designated by the orange circle) by clipping the cluster from the stem using scissors and allowing the flowers to drop into a sampling bag.


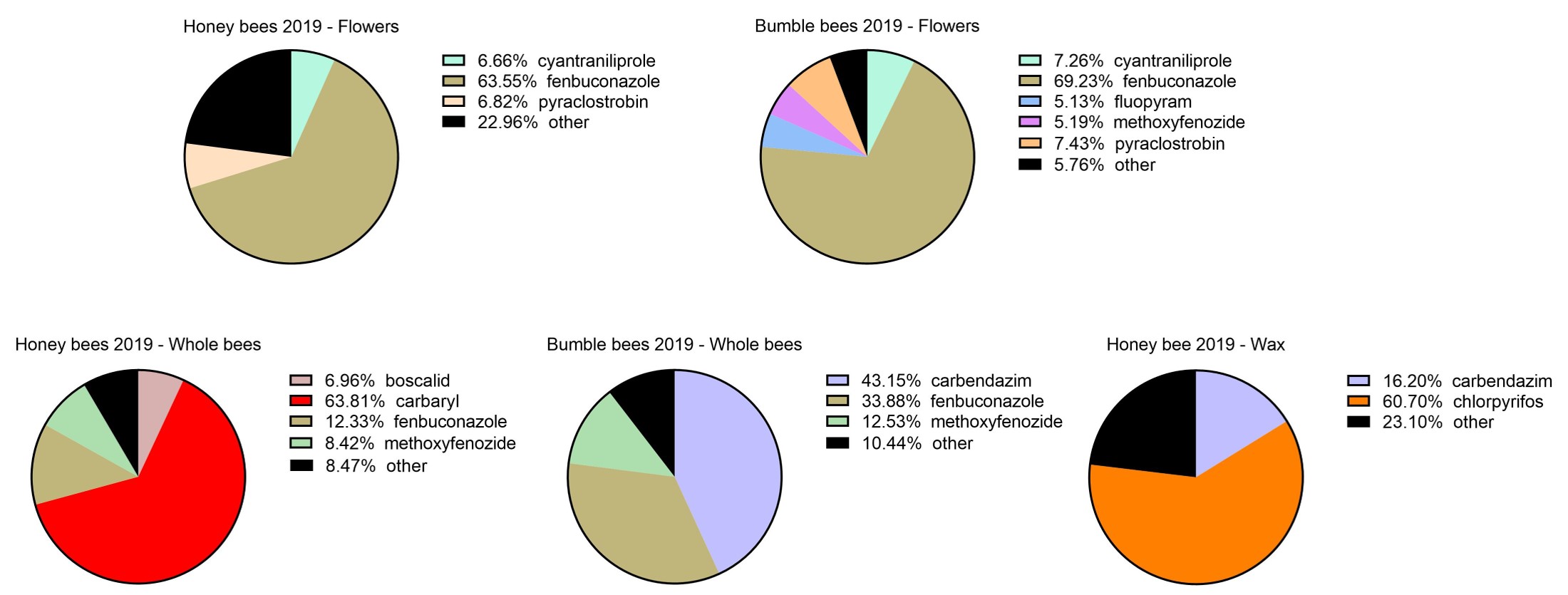


**Fig. S3.** Contribution of individual active ingredients to the risk quotient calculations for each sample type. This was determined using contact LD50 values. Toxicity data for *Apis mellifera* and *Bombus terrestris* were used depending on the sample type.

Graph created in GraphPad Prism 9^33^.


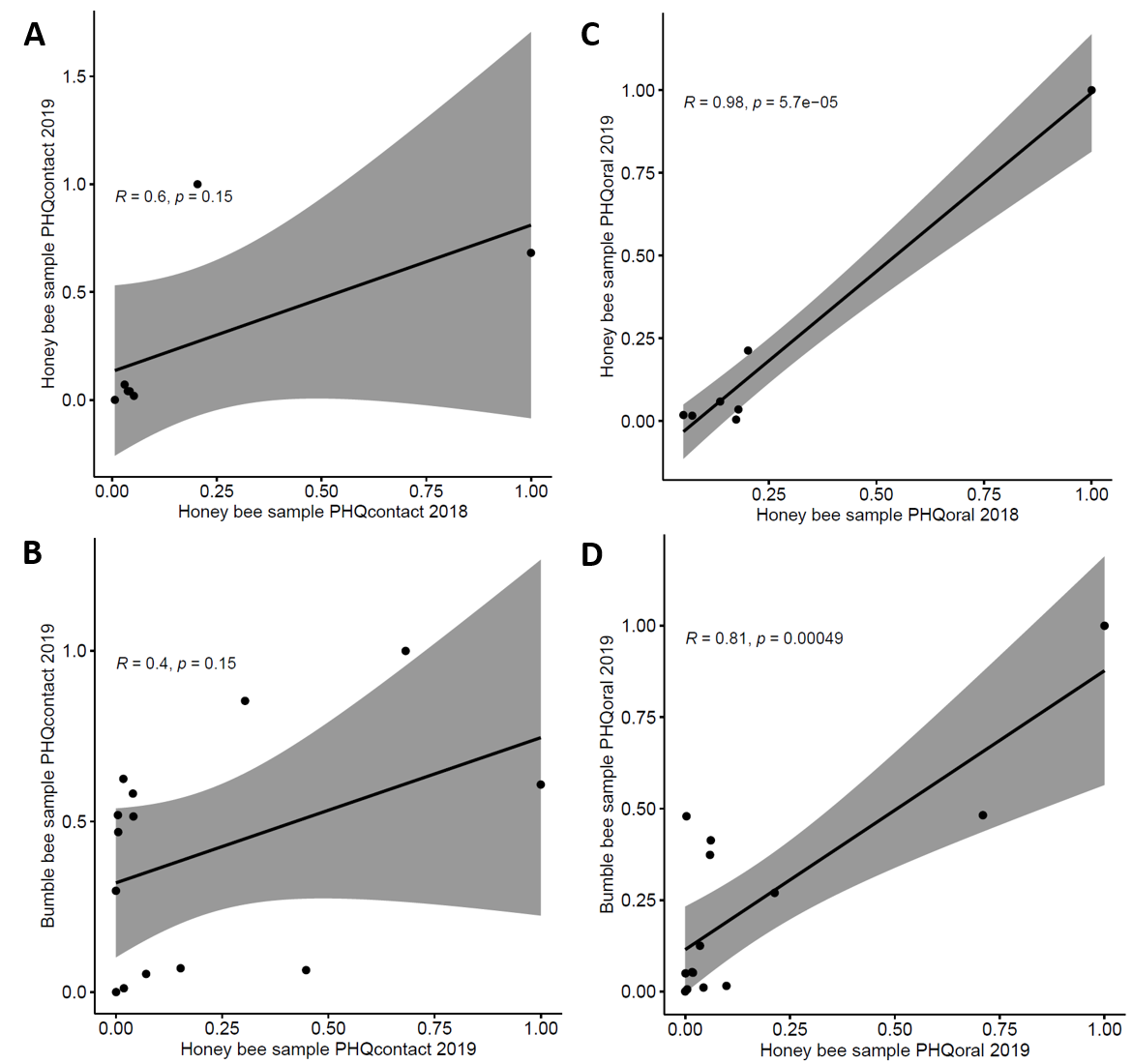


**Figure S4.** Correlation between sample pollen hazard quotients (PHQ, equivalent to risk quotients, RQ) between years (A, C) and between species (B, D). RQs were calculated based on contact (A, B) and oral (C, D) toxicity data. Graph created in R version 4.1.1^32^ with the package ggplot2^101^.
